# MOF-Based Lithium Oxygen Batteries: Status and Prospects from Material Design to System Integration

**DOI:** 10.1021/acscentsci.6c00644

**Published:** 2026-06-11

**Authors:** Shuaiyu Gao, Yuqing Dong, Malin Li, Yuelin Zhao, Genban Sun, Jihong Yu

**Affiliations:** † Beijing Key Laboratory of Energy Conversion and Storage Materials Institution, College of Chemistry, 47836 Beijing Normal University, Beijing 100875, China; ‡ State Key Laboratory of Inorganic Synthesis and Preparative Chemistry, College of Chemistry, International Center of Future Science, 12510 Jilin University, Changchun 130012, China

## Abstract

Lithium oxygen batteries (LOBs) offer exceptional theoretical energy density but face formidable challenges due to the sluggish kinetics of the oxygen reduction and evolution reactions (ORR/OER). This review delineates the pivotal role of metal–organic frameworks (MOFs) in overcoming these hurdles. We systematically dissect how the unique structural programmability, ultrahigh surface area, and atomically dispersed active sites of MOFs and their derivatives are ingeniously exploited to engineer high-performance cathodes, functional separators, and solid-state electrolytes. The discussion is extended to pristine MOFs, MOF-derived carbons, and heteroatom-doped composites, highlighting their efficacy in regulating Li_2_O_2_ growth and reducing overpotentials. Furthermore, strategic hybridization with conductive matrices such as carbon nanotubes and MXenes to enhance electron transfer is elaborated. Finally, we provide critical perspectives on future research directions, encompassing stability enhancement, artificial intelligence-guided discovery, and advanced interfacial engineering, charting a course for MOF-based materials to propel the development of next-generation LOBs.

## Graphical abstract

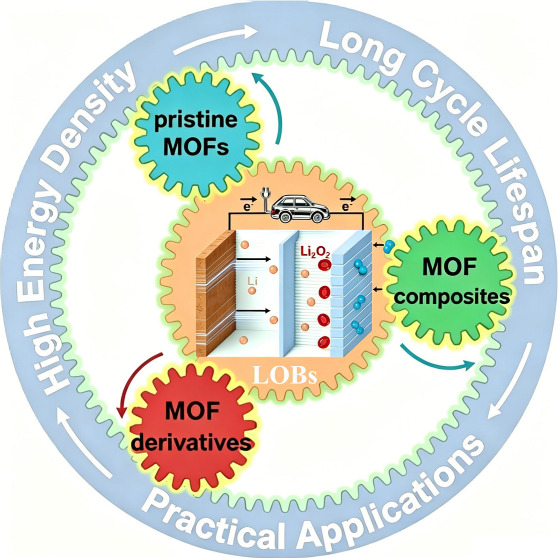

## 1 Introduction

The exponential growth of global economic activities has driven an unprecedented surge in energy consumption, while conventional fossil fuels (including coal, petroleum, and natural gas) are fundamentally constrained by finite geological reserves and rapidly accelerating depletion rates. This critical juncture of energy security and sustainability imperatives demands immediate breakthroughs in alternative energy paradigms and disruptive energy storage innovations.^1^ Consequently, global research efforts have converged on advancing sustainable renewable energy systems and next-generation energy storage architectures to address these existential challenges.^2–4^ Within this technological landscape, electrochemical energy conversion and storage platforms have emerged as a focal point of research due to their inherent scalability, operational efficiency, and minimal environmental footprint.^5–7^ The energy storage landscape has witnessed the emergence of a diverse array of advanced systems, including lithium-ion batteries (LIBs), lithium sulfur batteries, zinc manganese configurations, and supercapacitive architectures.^8,9^ Among these, intercalation-based LIBs have established themselves as the dominant electrochemical energy storage platform over the past two decades, revolutionizing portable electronics and electrified transportation.^10^ Despite the progress accomplished to date, even when fully developed, the maximum energy storage density that LIBs can deliver is too low to meet the demands of key markets, such as transport, in the long term. Reaching beyond the horizon of LIBs is a formidable challenge.^11^

The strategic emergence of lithium oxygen batteries (LOBs) technology offers a paradigm-shifting solution, leveraging open-system oxygen reduction chemistry to achieve an order-of-magnitude enhancement in theoretical energy density compared to conventional LIBs, while maintaining compatibility with existing lithium-based electrochemical infrastructure.^12^ The theoretical energy density of rechargeable LOBs reaches up to 11400 Wh kg^–1^ (calculated based on the mass of metallic lithium), which approaches the energy density of gasoline (12800 Wh kg^–1^).^13,14^ Even when accounting for the mass of the discharge product Li_2_O_2_, the energy density of LOBs remains as high as 3500 Wh kg^–1^, with an actual energy density of 1700 Wh kg^–1^ (approaching the actual energy density of gasoline)^14^ (Figure 1a). Owing to their open oxygen supply configuration, LOBs can directly utilize the oxygen component from ambient air as a reactant, obviating the need for additional oxidant storage, thereby significantly minimizing system complexity and cost.^15,16^ This synergistic combination of high energy density, environmental benignity, and economic viability positions LOBs as highly promising for renewable energy storage and efficient energy conversion. Notwithstanding decades of intensive research and notable advancements, their practical implementation remains hindered by multifaceted challenges: electrolyte decomposition-induced side reactions, lithium dendrite-driven short-circuit hazards, electrode/electrolyte interfacial parasitic reactions, and sluggish reaction kinetics. These issues severely constrain battery cycle life and energy efficiency, necessitating further solutions through innovative material design and mechanistic insights.^15,17–19^

**Figure 1:**
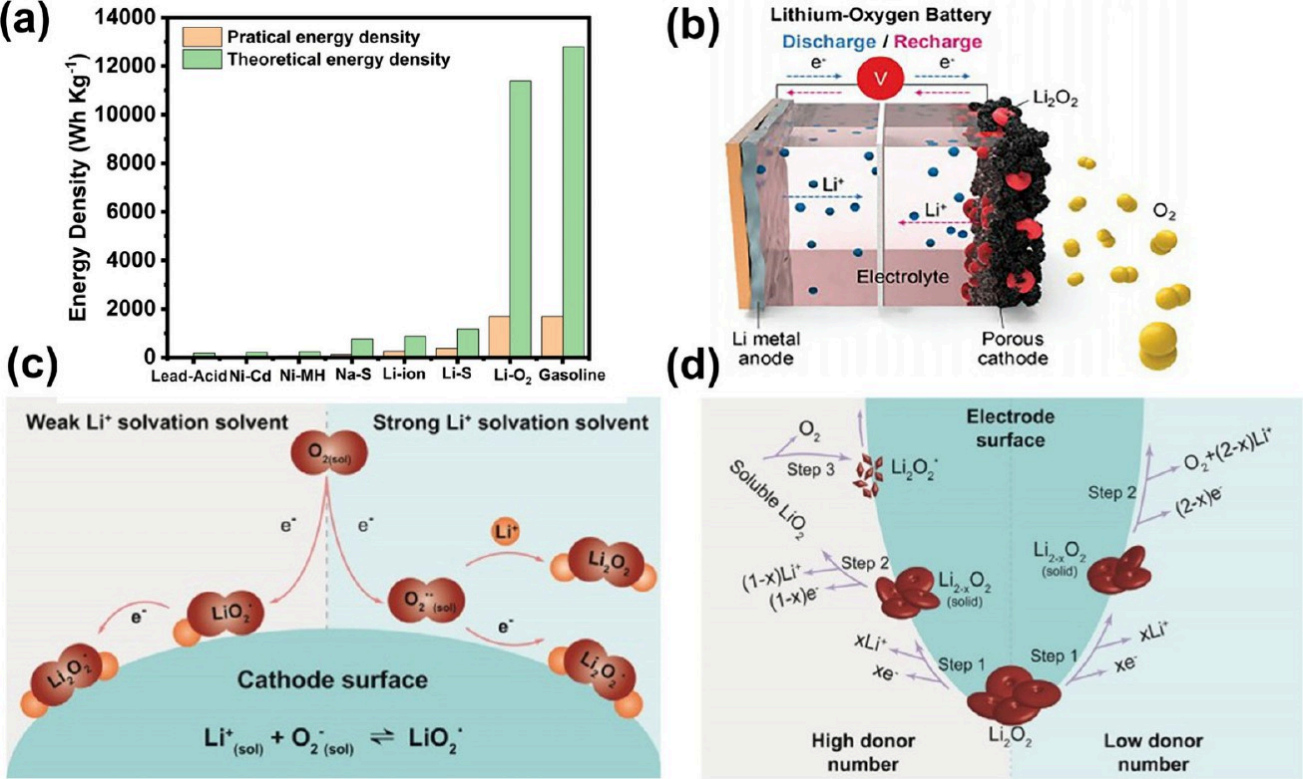
(a) Comparison of the theoretical energy density of different battery systems and gasoline. (b) The configuration of LOBs. Reproduced with permission from ref 15. Copyright 2020 American Chemical Society. (c) Different discharge mechanisms. (d) Two different charge mechanisms. Reproduced with permission from ref 23. Available under a CC-BY 4.0 license. Copyright 2023 Elsevier Guo et al.

The canonical configuration of LOBs comprises three essential components: a lithium metal anode, an ion-conductive electrolyte, and a porous oxygen-breathing cathode^20^ (Figure 1b). Their operational mechanisms center on the oxygen reduction reactions (ORR) during discharge and oxygen evolution reactions (OER) during recharge.^21,22^ During the ORR in the discharge phase, O_2_ undergoes electron transfer to generate metastable lithium superoxide (LiO_2_) intermediates. These species follow distinct nucleation pathways depending on the electrolyte environment: electrochemical reduction dominates to form conformal Li_2_O_2_ films via surface-mediated growth mechanisms, whereas disproportionation reactions prevail to yield particulate Li_2_O_2_ discs through solution-phase growth pathways.^23^ Crucially, high donor number (DN) solvents facilitate LiO_2_ solvation, enabling solution-phase precipitation of toroidal Li_2_O_2_ via homogeneous disproportionation (Figure 1c). Conversely, in low-DN electrolytes, LiO_2_ exhibits enhanced surface stabilization on catalytic substrates. These surface-adsorbed intermediates subsequently participate in either disproportionation or secondary electrochemical reduction processes, ultimately generating continuous Li_2_O_2_ coatings on electrode surfaces (Figure 1c).^15,23^

The OER during the charging phase involves the electrochemical decomposition of Li_2_O_2_ to generate oxygen gas and lithium ions. Current mechanistic understanding proposes two distinct pathways for this oxidative process: direct single-step oxidation and sequential two-step oxidation. In the single-step mechanism, Li_2_O_2_ undergoes direct two-electron oxidation with concomitant O_2_ release.^24^ Conversely, the two-step pathway involves initial single-electron oxidation at reduced overpotentials followed by subsequent electron transfer at elevated charging potentials.^25^ Notably, the potential existence of LiO_2_ as a transient intermediate remains challenging due to the detection limitations of conventional *in situ* spectroscopy for such metastable species. Recent mechanistic investigations employing advanced spectroscopic techniques have revealed that solvent DN plays a critical role in determining reaction pathways.^26^ High-DN solvents facilitate solution-mediated disproportionation reactions that promote crystalline Li_2_O_2_ formation through soluble LiO_2_ intermediates. In contrast, the solvent with a low number of donors is conducive to the formation of Li_2–x_O_2_, which is further oxidized to release oxygen^23^ (Figure 1d). The electrochemical reaction in the LOBs system is highly complex, which is because ORR and OER need to occur synergistically at the unique gas–liquid–solid three-phase interface. Moreover, the intrinsic insulating nature of the discharge product Li_2_O_2_ severely hinders reaction kinetics, giving rise to critical issues such as high overpotential, low round-trip efficiency, limited capacity, and cyclability degradation challenges. These issues are further exacerbated under practical operating conditions, where cycle stability deteriorates significantly. The industrialization of LOBs further confronts multifaceted challenges. On one hand, irreversible byproducts (Li_2_CO_3_) and electrolyte decomposition during charging induce continuous degradation of battery components and passivation of electrocatalytic active sites. On the other hand, these parasitic side reactions not only cause irreversible capacity loss but also pose potential risks to the thermal safety of the battery system.^27,28^

The inherent complexity of these challenges substantially impedes the progression of high-performance LOBs toward practical engineering implementation. To address these technological bottlenecks, coordinated multidisciplinary innovation at fundamental research levels must be pursued through three strategic optimization pathways: 1) Rational design of advanced cathode architectures. Optimal LOBs cathodes require simultaneous fulfillment of critical design criteria: (i) engineering high-density intrinsic catalytic active sites, (ii) establishing three-dimensional (3D) continuous conductive networks, and (iii) constructing hierarchical porous frameworks compatible with Li_2_O_2_ deposition dynamics.^29–31^ The electrochemical energy storage characteristics and reaction reversibility of the cathode material are fundamentally governed by the surface redox pathway, where synergistic modulation of material composition, morphological features, and microstructural parameters dictates the reaction kinetics. High-efficiency catalytic systems can significantly enhance kinetic processes by lowering the activation barriers for Li_2_O_2_ formation/decomposition. This optimization strategy is the reduction of redox reaction overpotentials, thereby synergistically improving critical performance metrics, including energy efficiency, gravimetric energy density, cycling durability, and rate capabilities in LOB systems.^32–34^ 2) Rational engineering of stabilized electrolyte systems. While conventional organic electrolytes have demonstrated partial success in LOBs implementation, their intrinsic chemical instability under extreme operational conditions remains a formidable challenge. This instability primarily manifests through parasitic solvent decomposition and irreversible accumulation of detrimental byproducts (Li_2_CO_3_/LiOH), ultimately compromising battery cyclability. Recent advances employ molecular dynamics simulations to elucidate Li^+^ solvation architectures, enabling the rational design of fluorinated ether-based electrolytes with tailored solvation energetics. Coupled with hybrid solid–liquid interphase engineering, this integrated approach establishes robust electrolyte systems capable of maintaining stable ionic transport pathways while effectively suppressing parasitic reactions, thereby achieving remarkable electrochemical stability in LOBs configurations.^20,21,35^ 3) Stabilizing lithium metal anodes. While lithium metal theoretically undergoes reversible stripping/plating processes during electrochemical cycling according to fundamental electrochemical principles, persistent challenges, including ramified dendrite proliferation, parasitic Li–H_2_O side reactions, and interfacial corrosion, inevitably induce detrimental dead Li formation, rapid capacity degradation, and critical safety concerns. Key strategies to mitigate these issues encompass: constructing mechanically robust solid electrolyte interphase (SEI) films, designing 3D conductive current collectors, and developing lithium alloy anode materials.^36,37^ These approaches collectively constitute a pivotal determinant for achieving simultaneous enhancements in gravimetric energy density, extended cycle longevity, and operational safety in next-generation LOB architectures.

Advancing the performance of LOBs necessitates the precise engineering of their constituent materials. Of particular significance are MOFs, whose crystalline porous structures (constructed through coordination between metal clusters and polyhedral organic linkers) have garnered substantial scientific interest in heterogeneous catalysis, molecular separation technologies, and electrochemical energy storage systems. Their unique structural merits, encompassing topology-tunable frameworks, ultrahigh surface area, and molecular-scale sieving capabilities, position these materials as paradigm-shifting candidates for functional material engineering in LOBs. The spatially ordered arrangement of catalytically active sites coupled with precisely modulated mass/charge transport pathways within MOF matrices enables unprecedented control over electrochemical processes. At the same time, its uniform and controllable ordered pores can construct a fast lithium-ion transport channel, realize ion-selective screening and uniform flux distribution, and physically block the shuttle of byproducts and inhibit the growth of lithium dendrites. The abundant metal sites and functional ligands can directionally regulate the solvation structure of the electrolyte, promote the dissociation of the lithium salt, improve the ionic conductivity and migration number, optimize the interface compatibility, stabilize the electrode–electrolyte interface, and enhance the thermal stability and electrolyte wettability. These merits effectively bridge the gap between nanoscale molecular design and macroscopic battery performance optimization.^38–40^ Compared to elemental metals, metal compounds, and heteroatom-doped carbon materials with spatially confined active sites, native MOFs constructed via direct metal–ligand coordination exhibit three distinctive advantages: (i) enhanced porosity (up to 90% free volume), (ii) abundant accessible active sites (surface density >5 sites nm^–2^), and (iii) accelerated ion diffusion pathways (ionic conductivity >10^–3^ S cm^–1^).^41^ Crucially, MOF catalytic properties can be precisely modulated through strategic coordination engineering of organic linker topology and metal cluster selection. In addition, postsynthetic modification strategies endow MOF derivatives with emergent functionalities beyond those of pristine frameworks. Of particular significance, conductivity-optimized MOF derivatives retain advantageous porous frameworks while achieving remarkable electronic conductivity improvements.^42^ Furthermore, the synergistic coupling of MOF matrices with functional components creates multicomponent systems that simultaneously expand structural diversity and amplify catalytic efficiency. These engineered attributes establish MOF-based functional materials as paradigm-shifting materials for advanced LOB applications.

To date, numerous researchers have employed MOF-based materials in LOBs, with existing studies primarily focusing on their multifunctional roles as critical components such as cathodes, anodes, separators, and electrolytes (Figure 2). However, systematic summaries of the MOF-based materials in LOBs remain scarce. Thus, this review highlights recent advancements in the use of pristine MOFs, MOF derivatives, and MOF composites in LOBs. Subsequently, it provides a detailed discussion on the research progress of MOF-based materials for anodes, cathodes, separators, and electrolytes, as well as the relationship between MOF structures and their performance. Finally, the review identifies the challenges associated with MOF applications in LOBs, offering guidance for the rational design of MOF-based materials with high energy density and superior cycling stability for advanced LOBs.

**Figure 2:**
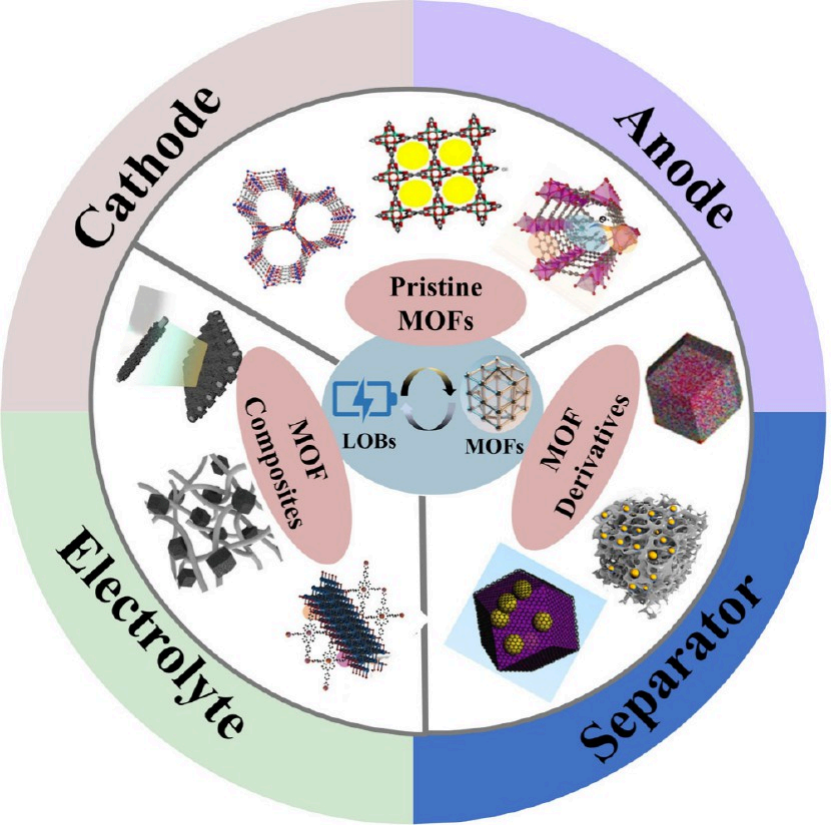
Schematic illustration of MOF-based catalysts applied in LOBs. Pristine MOFs: Co-MOF-74. Reproduced with permission from ref 43. Copyright 2017 Royal Society of Chemistry. Dy-BTC. Reproduced with permission from ref 44. Copyright 2020 Royal Society of Chemistry. Mn-MOF-74-FcA. Reproduced with permission from ref 45. Copyright 2024 Elsevier. MOF Composites: CMT@MXene. Reproduced with permission from ref 46. Copyright 2023 WILEY-VCH. Co_3_O_4_@NiCo_2_O_4/_CC. Reproduced with permission from ref 47. Copyright 2020 Elsevier. MOF-C/CNT. Reproduced with permission from ref 48. Copyright 2019 WILEY-VCH. MOF Derivatives: NiCoP@PNCReproduced with permission from ref 49. Copyright 2024 Elsevier. Co_3_O_4_/C. Reproduced with permission from ref 50. Copyright 2019 American Chemical Society. CoS_2_(CoS)@NC. Reproduced with permission from ref 51. Copyright 2021 American Chemical Society.

## 2 Synthesis of the MOF-Based LOBs Electrode

### 2.1 Overview of MOFs

MOFs have emerged as a novel class of crystalline porous materials formed through the self-assembly of metal ions/clusters as central nodes with organic ligands. First systematically conceptualized and named by American chemist Omar M. Yaghi in 1995, this materials family has evolved into a well-established research domain through three decades of scientific exploration.^52^ As archetypal representatives of metal–organic materials, MOFs demonstrate exceptional potential in cutting-edge applications owing to their unparalleled structural programmability and compositional diversity. These nanoscale porous architecture materials uniquely combine quantum confinement effects inherent to nanoscale architectures with long-range periodic ordering and anisotropic characteristics typical of crystalline substances. Particularly noteworthy is the synergistic integration of precisely engineered porous architectures with tunable chemical functionalities, positioning MOFs as next-generation functional materials that bridge fundamental scientific inquiry with practical engineering applications.^53,54^

### 2.2 Characteristics of MOF-Based Materials

The formation of MOFs originates from the directional self-assembly process between central metal ions/clusters and bridging organic ligands. By orchestrating the strategic selection of metallic nodes and organic linkers, researchers can precisely engineer crystalline materials with tailored pore architectures while achieving customized regulation of exceptional surface areas. This synthetic versatility enables systematic optimization of host–guest interactions at molecular dimensions, unlocking unprecedented opportunities in advanced adsorption and catalysis applications.^52^ MOFs with precisely tunable chemical compositions and crystalline porous architectures exhibit three distinctive characteristics: (1) Crystalline open frameworks. Porosity constitutes an intrinsic hallmark of MOFs, arising from the coordination-directed formation of open frameworks between metallic nodes and organic linkers. Remarkably, the pristine porous frameworks can be judiciously engineered into hierarchical architectures through postsynthetic modifications. This structurally ordered porosity ensures homogeneous distribution of active sites within the matrix, facilitating continuous electrochemical reaction pathways. Furthermore, the encapsulation of functional guests within the porous scaffold effectively mitigates detrimental agglomeration phenomena, thereby engendering enhanced electrochemical performance.^41,55–57^ (2) Tailorable metallic nodes. Metal clusters serve as pivotal determinants governing both the porous topology and functional characteristics of MOFs.^58^ It is worth noting that bimetallic MOFs with synergistic electronic effects can be synthesized by orderly selection of metal nodes, which greatly promotes charge transfer kinetics and optimizes the electron distribution, thereby achieving excellent conductivity and operational stability. Capitalizing on the atomic-level isolation inherent to MOF matrices, researchers have successfully developed MOF-derived single-atom catalysts (SACs) featuring atomically dispersed active centers. This structural precision maximizes the intrinsic catalytic activity of metallic species while ensuring exceptional durability under reactive conditions.^59^ (3) Multifunctional organic linkers. Serving as structural spacers, organic ligands not only isolate metallic nodes to generate monodisperse active sites but also enable precise control over framework functionality. The rich diversity of functional groups (e.g., carboxylates, amines) facilitates heterogeneous nucleation and epitaxial growth on various substrates through solution-phase interactions.^41,60^ For MOF derivatives, different organic ligands are not only sources of carbon, but also sources of other nonmetallic dopants, such as nitrogen, sulfur, and phosphorus.^61^ This dopant-induced electronic redistribution enhances charge storage capacity and reaction kinetics. Moreover, *in situ* reactions between heteroatoms and metal centers yield high-performance compounds (metal sulfides, phosphides, or metal–N_*x*_ moieties) that significantly improve battery metrics.^62,63^ Compared with conventional materials, MOFs’ unique combination of structural programmability and multifunctional integration establishes unparalleled advantages for developing next-generation high-performance battery systems.^64^

### 2.3 Synthesis of MOFs

As previously elucidated, MOFs are architecturally composed of two primary constituents: metal-based nodes and multifunctional organic linkers. These crystalline frameworks are typically synthesized under mild reaction conditions through the coordinated assembly of metallic species with organic bridging ligands. The attainment of well-defined porosity and crystallinity necessitates precise control over thermodynamic and kinetic parameters during nucleation processes. Over the past two decades, substantial methodological advancements have yielded a diverse array of synthesis strategies (including hydrothermal synthesis, solvothermal crystallization, and mechanochemical approaches) that enable the tailored fabrication of MOF architectures with programmable functionalities (Figure 3). This methodological evolution has significantly expanded the structural diversity and application spectrum of MOF-based materials.^65,66^

**Figure 3:**
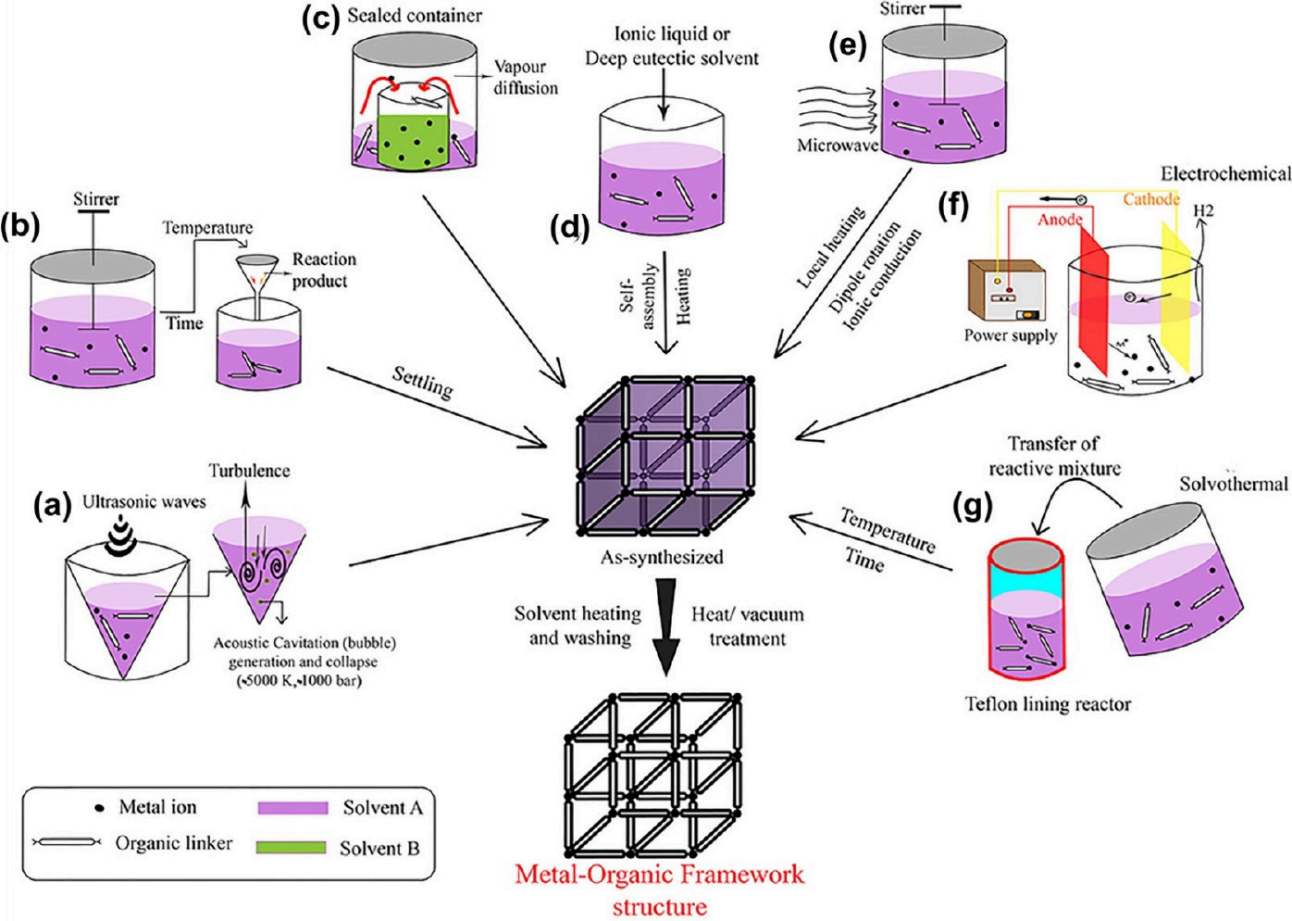
Scheme of different synthesis methods of MOF. Reproduced with permission from ref 90. Copyright 2020 Elsevier. (a) sonochemical synthesis method, (b) conventional solution method, (c) diffusion synthesis method, (d) ionothermal process method, (e) microwave-assisted synthesis method, (f) electrochemical synthesis method, and (g) solvothermal synthesis method.

#### 2.3.1 Conventional Synthesis Methods

Conventional MOF synthesis predominantly relies on electric heating systems without parallel processing capabilities, where thermal parameters critically govern structural evolution. The synthesis protocols are fundamentally classified into solvothermal (SVT) and nonsolvothermal (non-SVT) routes based on temperature–pressure coordination. SVT processes employ sealed reactors operating at autogenous pressures with temperatures exceeding solvent boiling points, whereas non-SVT methods utilize ambient pressure conditions at or below solvent boiling temperatures, offering simplified operational requirements (Figure 3g).^67,68^ Hydrothermal synthesis, a specialized SVT variant using aqueous media, enables precise modulation of framework architectures through controlled pressure gradients, thermal profiles, solvent matrices, and precursor concentrations.^69^ Notably, several benchmark MOFs, including MOF-5, MOF-74, MOF-177, HKUST-1, and ZIF-8, have been successfully fabricated via direct precipitation methodology under standard conditions through judicious combination of precursor components.^70–72^ This approach demonstrates spontaneous crystallization kinetics in selected MOF systems. As such, higher temperatures result in more condensed structures.^73,74^ However, thermal fluctuations may induce structural disorder in crystalline domains, while prolonged reaction durations may trigger framework degradation through ligand dissociation pathways.^75^

#### 2.3.2 Microwave-Assisted Synthesis

The microwave-assisted (MWA) synthesis mechanism originates from the resonant coupling between electromagnetic fields and mobile charge carriers within the reaction medium, including polar solvent molecules, ionic species, or conductive electrons in dispersed solids. MWA synthesis of MOFs is typically conducted at temperatures exceeding 100 °C with remarkably shortened reaction durations (<60 min), as illustrated in Figure 3e.^76^ Advanced parametric control allows systematic optimization through modulation of solvent dielectric properties, irradiation time (seconds to minutes), thermal profiles, microwave power density, and stoichiometric ratios. This approach demonstrates superior efficiency over conventional electric heating, enabling rapid crystallization kinetics that favor the formation of nanocrystalline domains with narrow size distribution.^76,77^ The benefits of MWA MOF synthesis, including crystallization acceleration, production of nanoparticles, and improved purity of products, are related to the rapid boiling of the solution as well as the nucleation rate.^69,76^

#### 2.3.3 Electrochemical Synthesis

Electrochemical synthesis of benchmark MOFs, including HKUST-1, MIL-100­(Al), and NH_2_-MIL-53­(Al), has been achieved through anodic dissolution in a customized electrochemical battery. Joaristi et al. systematically investigated critical process parameters (including solvent dielectric constant, electrolyte composition, thermal conditions, and current density), demonstrating their profound impact on MOF crystallinity and surface topology (Figure 3f).^78^ Cao’s group pioneered a cost-effective electrosynthetic strategy for the *in situ* growth of Ni-MOF architectures on nickel foam substrates. The self-assembled floral nanostructures function as binder-free electrodes, delivering exceptional areal capacitance of 5.1 C cm^–2^ at 2.0 mA cm^–2^ with remarkable cycling stability.^79^ Mueller’s team established a universal electrochemical platform for synthesizing diverse Cu-/Zn-based MOFs. Their parametric study mapped the effects of metallic electrodes (Zn, Cu, Co) combined with various linkers under different electrochemical regimes, establishing scalable protocols for industrial translation.^80^ Schlesinger et al. conducted comparative studies between solvothermal and electrochemical syntheses of HKUST-1 under ambient pressure conditions, revealing method-dependent variations in porosity profiles and catalytic performance metrics.^81^

#### 2.3.4 Mechanochemical Synthesis

Mechanochemical transduction phenomena enable unique solid-state transformations through controlled application of mechanical energy. When subjected to tribochemical collisions, crystalline lattices undergo structural disordering through bond cleavage and surface reconstruction, generating localized hotspots (>1000 K) that induce vibrational excitation sufficient for bond reorganization.^82^ Pioneering work by Pichon et al. demonstrated solvent-free mechanochemical (MCL) synthesis of porous Cu­(INA)_2_ frameworks via room-temperature grinding of copper acetate and precursors. Remarkably, this protocol achieved quantitative yields of phase-pure crystals with well-defined 3D connectivity within 10 min, as verified by synchrotron X-ray powder diffraction analysis.^83^ Their subsequent systematic investigation of MCL-MOF synthesis reveals a crystallinity success rate of 67% across diverse metal–ligand combinations (Ni^2+^/Cu^2+^/Zn^2+^ with carboxylate linkers), identifying 38 crystalline phases, including novel coordination polymers and nanoporous architectures like HKUST-1.^84^ The growing adoption of MCL-MOF synthesis stems from its inherent green chemistry advantages: solvent-free operation, ambient condition compatibility, and near-quantitative atom economy. These eco-benign characteristics align with sustainable materials manufacturing paradigms while enabling precise control over framework defects and surface reactivity.^85^

#### 2.3.5 Other Synthesis Strategies

Beyond the aforementioned approaches, emerging MOFs synthesis methodologies (including sonochemical methods,^86^ ionic liquid-based synthesis media,^87^ microfluidic MOFs synthesis systems,^88^ and xerogel-conversion-based MOFs synthesis^89^) offer distinct advantages under specific conditions. These techniques are well-suited for fabricating electrochemical devices, leveraging their unique attributes to enable precise control over MOFs’ morphology, composition, and interfacial properties.

### 2.4 Screening Methods of MOF Materials

#### 2.4.1 Traditional Screening Methods

The screening paradigm for MOFs has undergone a significant transformation from empirical trial-and-error experimentation to theoretically guided computational simulation. In the early stages of research, scientists predominantly relied on chemical intuition and repetitive experimentation, systematically adjusting metal nodes, organic linkers, and critical reaction parameters, including solvent environment, temperature field, and pH values, to explore novel material systems. While this approach successfully identified landmark structures such as MOF-5 and ZIF-8, its inherent inefficiency substantially hindered systematic optimization of material properties.

1. Establish multimodal in-situ platforms combining synchrotron and cryo-electron microscopy to monitor MOF electrodes from atomic to micrometer scales.

Density functional theory (DFT)-based simulations provide reliable predictions of MOF stability, hierarchical porosity, and gas adsorption behavior, while Monte Carlo sampling and molecular dynamics simulations serve as indispensable tools for investigating diffusion kinetics and complex interfacial interactions. Notably, grand canonical Monte Carlo (GCMC) simulations have emerged as the gold standard for evaluating gas storage capacity in MOFs, and first-principles calculations offer fundamental insights into the electronic structure of catalytically active sites. Nevertheless, these high-accuracy computational methods remain computationally demanding, currently limiting their application to screening of finite structural libraries.

#### 2.4.2 High-Throughput Computational Screening

The development of MOFs has experienced exponential growth, with experimentally synthesized crystal structures experimental MOFs (eMOFs) now exceeding 90000 entries, the majority of which are archived in the Cambridge Structural Database.^64^ Nevertheless, the design space for MOFs is virtually limitless. While this immense structural diversity provides vast opportunities for material optimization in specific applications, it simultaneously poses significant challenges for conventional experimental screening methods. In computational materials science, breakthroughs in high-performance computing and advanced simulation techniques have enabled the systematic generation of MOFs based on chemical design principles. In a landmark 2011 study, Wilmer et al.^91^ pioneered a “top-down” design strategy to construct an extensive database comprising 137953 hypothetical MOFs (hMOFs). This was further advanced in 2017 by Colón’s team,^92^ who utilized edge-transitive topological nets algorithms coupled with Topology-Based Clustering and Comparison code (ToBaCCo) code to develop a structural database encompassing 41 topological types and totaling 13512 hMOFs.

Molecular simulation-based high-throughput computational screening has emerged as the most efficient research paradigm for evaluating and optimizing MOF materials. This methodology typically follows a systematic four-step workflow: (1) construction or selection of target MOF structural databases; (2) preliminary screening based on key structural characteristics such as surface area and pore size distribution; (3) precise prediction of critical performance metrics including adsorption separation efficiency and catalytic activity through molecular simulations; (4) comprehensive evaluation of candidate materials using multidimensional assessment criteria under realistic operating conditions, ultimately identifying optimal MOF candidates.

The judicious selection of databases serves as the cornerstone for high-throughput screening of MOFs, critically determining the predictive performance of machine learning (ML) models. Current MOF databases are primarily categorized into two systems: eMOFs and hMOFs. Experimental databases (e.g., the Computation-Ready, Experimental MOF database (CoRE MOFs) developed by Snurr’s group.^93^) are constructed by systematically organizing reported crystal structures, offering the distinct advantage of rich structural diversity coupled with verified synthesis pathways. In contrast, computational databases (exemplified by Li’s^94^ Multivariate MOF database (MTV MOFs)) are built upon chemical assembly principles, characterized by their structural regularity and design predictability. During the prescreening phase, researchers typically employ specialized computational tools (Zeo++, RASPA,^95^ LAMMPS,^96^ etc.) to systematically analyze key structural parameters, including geometric features, topological configurations, chemical compositions, and adsorption potential distributions. Screening thresholds are then established based on target application scenarios. Commonly adopted evaluation metrics encompass critical physical parameters such as the largest cavity diameter, limiting pore diameter, material density, porosity, pore volume, and specific surface area, enabling efficient screening with reduced computational costs through multidimensional assessment.

Molecular simulation techniques serve as the cornerstone methodology in high-throughput screening of MOFs, enabling accurate prediction of diverse critical material properties. GCMC^97^ simulations are predominantly employed to evaluate equilibrium adsorption characteristics, while MD^98^ simulations excel in elucidating nonequilibrium processes such as dynamic diffusion behaviors. Notably, innovative approaches based on classical DFT^99^ and lattice GCMC^100^ methods have been progressively implemented for MOF property prediction, offering novel technical pathways for high-throughput screening investigations.

#### 2.4.3 Machine Learning

The exponential expansion of MOF varieties has surpassed the capacity of conventional high-throughput screening to comprehensively evaluate application-specific performance across diverse scenarios, while the vast multidimensional data sets generated necessitate advanced big data analytics. Artificial intelligence (AI) and ML have emerged as a cutting-edge research frontier in auxiliary screening due to their inherent alignment with these demands. By extracting implicit statistical patterns from high-dimensional data, ML trains predictive models on screening data sets to accurately determine critical parameters of MOF adsorbents (including adsorption capacity, selectivity, and adsorption isotherms), thereby dramatically accelerating the discovery of high-performance adsorbents.^97^ Advanced algorithms such as Artificial Neural Networks^101^ and Gradient Boosting Machines^102^ are particularly prevalent in this domain.

In the whole process of ML, the selection of descriptors is not a simple data preprocessing step, but the core lifeline that determines the upper limit of the model and defines the learning direction. Its significance is decisive and irreversible. The descriptor is the only bridge between the original information and the model cognition. Krishnapriyan et al.^97^ pioneered the Persistence Image Approach, which integrates topological descriptors with regularized structural features to generate vectorized inputs for ML models, enabling successful prediction of zeolite adsorption performance. While such descriptors provide intuitive representations of fundamental MOF properties with strong experimental correspondence, they yield models with limited generalizability. For chemical characteristics, descriptors including atom types, unsaturated metal sites, metal cluster ratios, electronegativity, and Henry’s constants (*K*_*H*_) are widely employed. Given their broad value distributions, feature engineering (such as logarithmic normalization of *K*_*H*_) is typically required.^103^ More innovatively, researchers are leveraging microscopic potential information like cohesive energy and Voronoi energy distribution functions to enhance model accuracy and transferability.^97^

ML model selection is primarily categorized into supervised and unsupervised learning paradigms. Supervised learning constructs predictive models by performing regression or classification tasks on descriptor data sets. Regression analysis establishes mappings between input variables and output properties, enabling precise performance prediction for test-set MOFs through algorithms including Least Absolute Shrinkage and Selection Operator, Linear Regression, Kernel Ridge Regression, Artificial Neural Networks, and Deep Learning. Classification tasks partition data sets into feature subsets based on output variables, determining descriptor thresholds and performance classification criteria, with prevalent algorithms encompassing Decision Trees, Random Forests, Support Vector Machines, and Bayesian Optimization.^97^ Unsupervised learning explores hidden patterns and structure–property relationships within unlabeled data sets, employing core methodologies such as K-means Clustering, Principal Component Analysis, and Singular Value Decomposition.^97^

In summary, ML-augmented high-throughput screening of MOFs has demonstrated remarkable advancements in recent years. This progress is driven by the exponential growth of MOF architectures and the continuous diversification of their application scenarios. Goal-directed HTCS has emerged as the predominant paradigm for discovering high-performance MOFs and deciphering structure–property relationships. However, the massive multidimensional data sets and substantial computational demands inherent to HTCS exhibit perfect synergy with ML capabilities. Their integration significantly enhances the efficiency and accuracy of MOF performance prediction, provides fundamental insights into material behavior, and informs rational design strategies. Looking forward, the convergence of multimodal data fusion (e.g., correlative microscopy–property analysis) and autonomous robotic experimentation will propel a paradigm shift from “screening existing materials” to “de novo design of novel architectures”, delivering transformative material solutions for energy and environmental technologies. Although current AI/ML efforts in MOF research primarily focus on high-throughput structural screening, their integration with LOB catalyst design represents a promising frontier for future intelligent material development.

### 2.5 Modification Strategy of MOF Materials for LOBs

In the rational design of key components for LOBs, MOFs display remarkable structural tailorability and promising application potential, owing to their unique porous architectures, tunable pore size distributions, and abundant accessible active sites. (Figure 4) However, MOF-based catalysts exhibit three major drawbacks in LOBs: during long-term cycling, stress from Li_2_O_2_ deposition/stripping, electrolyte erosion, and redox attack cause structural collapse and active site agglomeration, leading to loss of catalytic capability; byproducts (such as Li_2_CO_3_, LiOH) continuously cover and poison active sites, blocking the reaction interface and deteriorating reaction kinetics; insufficient interfacial stability triggers electrolyte decomposition, anode and catalyst corrosion, increasing impedance, reducing reversibility, and accelerating cycle life decay. To fully exploit these inherent structural advantages and thereby comprehensively enhance stability, charge-transfer kinetics, and overall electrochemical performance, strategic design and systematic optimization of MOFs and their derivatives across multiple dimensions are highly desirable. Specifically, current research efforts are concentrated on the following four critical aspects: conductivity engineering, catalytic activity augmentation, structural reinforcement, and mass transport optimization. MOF-based catalysts achieve high capacity, low overpotential, and long cycle life through multidimensional optimization: hierarchical pores enable Li_2_O_2_ storage for high capacity; electronic tuning (doping/defects) reduces ORR/OER barriers for low overpotential; interfacial stabilization suppresses side reactions for extended cycle life.

**Figure 4:**
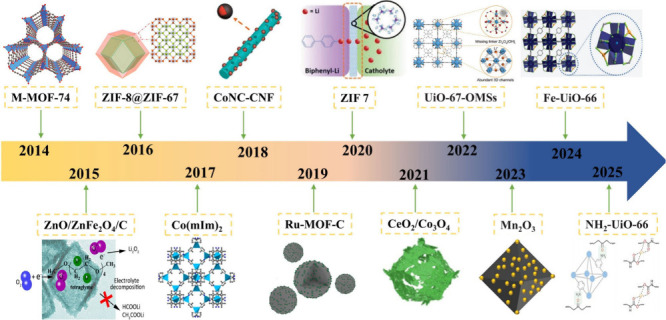
Progress of MOF-based materials in LOBs from 2014 to 2025. 2014: M-MOF-74. Reproduced with permission from ref 104. Copyright 2014 Wiley-VCH. 2015: ZnO/ZnFe_2_O_4_/C. Reproduced with permission from ref 105. Copyright 2015 American Chemical Society. 2016: ZIF-8@ZIF-67. Reproduced with permission from ref 106. Copyright 2016 American Chemical Society. 2017: Co­(mIM)_2_. Reproduced with permission from ref 107. Copyright 2017 American Chemical Society. 2018: CoNC–CNF. Reproduced with permission from ref 108. Copyright 2018 Wiley-VCH. 2019: Ru-MOF-C. Reproduced with permission from ref 109. Copyright 2019 American Chemical Society. 2020: ZIF 7. Reproduced with permission from ref 110. Copyright 2020 Wiley-VCH. 2021: CeO_2_/Co_3_O_4._ Reproduced with permission from ref 111_._ Copyright 2021 Elsevier. 2022: UiO-67-OMSs. Reproduced with permission from ref 112. Copyright 2022 Wiley-VCH. 2023: Mn_2_O_3_. Reproduced with permission from ref 113. Copyright 2023 Wiley-VCH. 2024: Fe-UiO-66. Reproduced with permission from ref 114. Copyright 2024 Wiley-VCH. 2025: NH_2_–UiO-66. Reproduced with permission from ref 115. Copyright 2025 Wiley-VCH.

#### 2.5.1 Conductivity Engineering

MOFs leverage ultrahigh specific surface area and rich porosity to provide abundant reaction space and numerous active sites for electrode processes. This structural advantage facilitates efficient oxygen adsorption/diffusion coupled with storage/transport of reaction products, establishing an optimized electrochemical microenvironment. Nevertheless, MOFs exhibit inherent limitations in electrical conductivity due to the predominantly covalent/ionic character of coordination bonds between organic linkers and metal ions, which restricts electron delocalization and causes inefficient bulk electron transport. This fundamental limitation impedes their direct application as the electrode of LOBs. The intrinsically poor electrical conductivity of MOFs is a key factor limiting their electrochemical performance. Currently, the most effective modification strategies fall into three categories: compositing with highly conductive matrices to construct a three-dimensional conductive network without damaging the original pores and active sites; high-temperature pyrolysis to convert MOFs into porous carbon-supported metals/compounds, enhancing conductivity and structural stability; and constructing intrinsically conductive MOFs that leverage π-π conjugation and metal redox activity for efficient electron transfer. The core mechanism of these approaches lies in building continuous electron pathways, optimizing electronic structures, and reducing charge transfer resistance, thereby enabling fast electron delivery to catalytic sites and ultimately improving the rate capability and cycling stability of batteries. Su et al.^116^ employed a one-pot solvothermal method to coat cobalt-triazolate MOFs, denoted as Co-MTA, onto carbon nanotubes (CNTs), constructing a Co-MTA-C nanocomposite with a 3D conductive network. Experimental characterization confirmed that CNTs induce electron transfer from Co-MTA to the carbon matrix, resulting in interfacial charge redistribution. Within the composite, the Co–N bond length shortened from 2.17 Å in the pristine MOF to 2.14 Å, significantly enhancing the electrostatic interaction between metal centers and ligands. Density of states analysis reveals the metallic character of the composite material, with a notable increase in states near the Fermi level, which facilitates electron transfer processes. Concurrently, the d-band splitting increases, broadening the orbital overlap energy region with oxygen-containing species and thereby optimizing the adsorption and conversion energy barriers for reaction intermediates. The high-valent cobalt active sites within the material directly react with LiO_*x*_ intermediates, inducing the formation and growth of nanosheet-like Li_2_O_2_ products and accelerating reaction kinetics. Furthermore, the 3D CNT network significantly enhances the electronic conductivity of electrode materials, as evidenced by a low contact resistance of 18.2 Ω for Co-MTA-C, and synergistically combines with open channels to effectively promote mass transport. As a novel two-dimensional (2D) material, MXenes have attracted significant interest owing to their excellent electrical conductivity. Zheng et al.^117^ employed a facile hydrothermal method to confine the growth of a cobalt-based MOF within the interlayers of layered Ti_3_C_2_MXene, successfully constructing a Co-MOF/Ti_3_C_2_ heterostructure. Total density of states (TDOS) analysis revealed a significant increase in the electronic states near the Fermi level for this heterostructure, thereby enhancing electron transfer capability. Projected density of states (PDOS) analysis at the cobalt sites further demonstrated enhanced catalytic activity through spin-state modulation, synergistically optimizing the adsorption energy of intermediates. The confinement effect imposed by the MXene interlayer spacing reduced the MOF size to approximately 20 nm, fully exposing open cobalt active sites and enhancing oxygen adsorption capacity. Concurrently, the formation of high-valent Co^2+^ species was observed, evidenced by a positive absorption edge shift of 0.1 eV in the X-ray absorption near edge structure (XANES) spectroscopy and a shortened Co–O bond length of 1.53 Å compared with the pristine MOF with a Co–O bond length of 1.56 Å in extended X-ray absorption fine structure (EXAFS) spectroscopy, which substantially strengthened the conversion of LiO_*x*_ intermediates (Figure 5a). Moreover, the high conductivity of MXene significantly lowered the charge transfer resistance to 67.04 Ω, markedly lower than the 92.11 Ω observed for the pure MOF. Consequently, the Co-MOF/Ti_3_C_2_ composite delivered an ultrahigh specific capacity of 24028 mAh g^–1^ at 100 mA g^–1^, a low overpotential of 0.99 V, and a stable cycling life of 116 cycles at 500 mA g^–1^.

**Figure 5:**
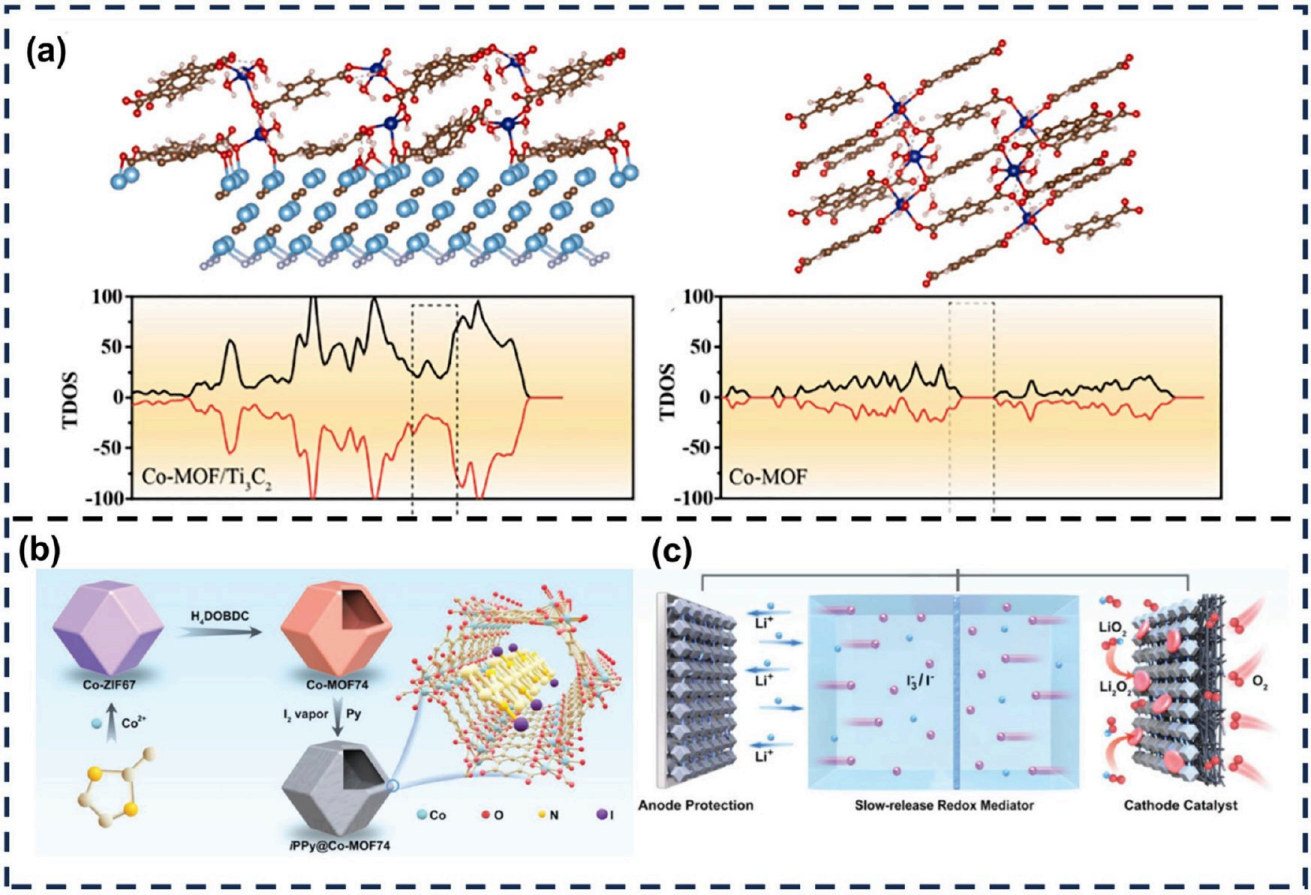
Optimized structures of (a) Co-MOF/Ti_3_C_2_ and Co-MOF. TDOS and PDOS of Co-MOF/Ti_3_C_2_ and Co-MOF. Reproduced with permission from ref 117. Copyright 2025 Elsevier. (b-c) Fabrication of Co-MOF74-H and iPPM via self-templated transformation and illustration of the “Trinity” design of LOB with iPPM. Reproduced with permission from ref 118. Copyright 2023 Wiley-VCH.

Conductive polymers, represented by polyaniline, polypyrrole, and polythiophene, exhibit good electrical conductivity, high electrochemical activity, and excellent flexibility. Coating MOFs with such conductive polymers forms a continuous conductive network on the MOF surface, effectively enhancing electron transport efficiency across the material and thereby compensating for the inherent poor conductivity of MOFs. For instance, He et al.^118^ employed a self-templated conversion strategy to fabricate a hollow cobalt-based MOF, Co-MOF74-H (Figure 5b–c). Subsequently, iodinated polypyrrole, denoted iPPy, was loaded into its pores via iodine vapor polymerization, yielding the composite material iPPM. This iPPM composite functions through a triple synergistic mechanism: the hollow MOF structure provides high specific surface area and open channels, effectively facilitating oxygen diffusion and Li_2_O_2_ storage; the I^–^/I_3_^–^ redox mediators (RMs) derived from the doped iPPy act as a slow-release redox shuttle, gradually diffusing into the electrolyte during charge and discharge cycles to reduce the polarization of LOBs; furthermore, serving as an anode coating, iPPM enhances the lithium-ion transference number and suppresses dendrite growth through a synergistic protective mechanism, while simultaneously blocking the shuttle effect of the oxidized RM species, thereby significantly mitigating anode corrosion.

#### 2.5.2 Catalytic Activity Augmentation

During the charge and discharge cycling, LOBs demand superior electrocatalytic activity to reduce charge and discharge overpotentials and enhance round-trip efficiency. Therefore, enhancing the catalytic efficiency of materials toward the key redox reactions in LOBs is critical. Through the rational design of the metal centers, ligand microenvironments, and active site distribution within MOFs, the adsorption and activation capabilities for reaction intermediates can be strengthened. This enhancement accelerates the reaction kinetics and ultimately improves the energy conversion efficiency of the batteries. Heteroatom doping effectively modulates the electronic structure and active site properties of MOFs. Lv et al.^119^ employed an ion-exchange strategy to introduce atomically dispersed ruthenium into a nickel-based hexaaminotriphenylene framework, Ni-HATP, successfully fabricating the bimetallic conductive material NiRu-HATP. Within this material, the d-band center of the Ru–N_4_ sites shifts significantly upward compared to that of Ni–N_4_ sites, strengthening interactions with oxygen-containing species. Efficient hybridization occurs between the d_*xz*_/d_*yz*_ orbitals of Ru and the σ*/π* orbitals of O_2_, forming low-occupancy antibonding orbitals. This hybridization dramatically enhances the O_2_ adsorption energy to – 1.47 eV, substantially higher than the 0.28 eV observed for Ni-HATP. Concurrently, the Ru–O bond length shortens to 1.80 Å, indicative of chemisorption, and more electrons are transferred to O_2_, resulting in an elongated O–O bond of 1.30 Å for NiRu-HATP compared to that for Ni-HATP (1.27 Å), effectively activating the oxygen molecule (Figure 6a). Furthermore, the strong adsorption of the key intermediate LiO_2_ by the Ru–N_4_ sites increases its local concentration (Figure 6b and c), inducing the formation of a uniform thin-film-like Li_2_O_2_ product between MOF nanowires instead of insulating large toroidal particles. This atomic-level ruthenium anchoring strategy precisely tunes the d-band center of the MOF, optimizing the oxygen reaction pathway and Li_2_O_2_ deposition behavior, thereby simultaneously enhancing the reaction kinetics, energy efficiency, and cycling stability of LOBs.

**Figure 6:**
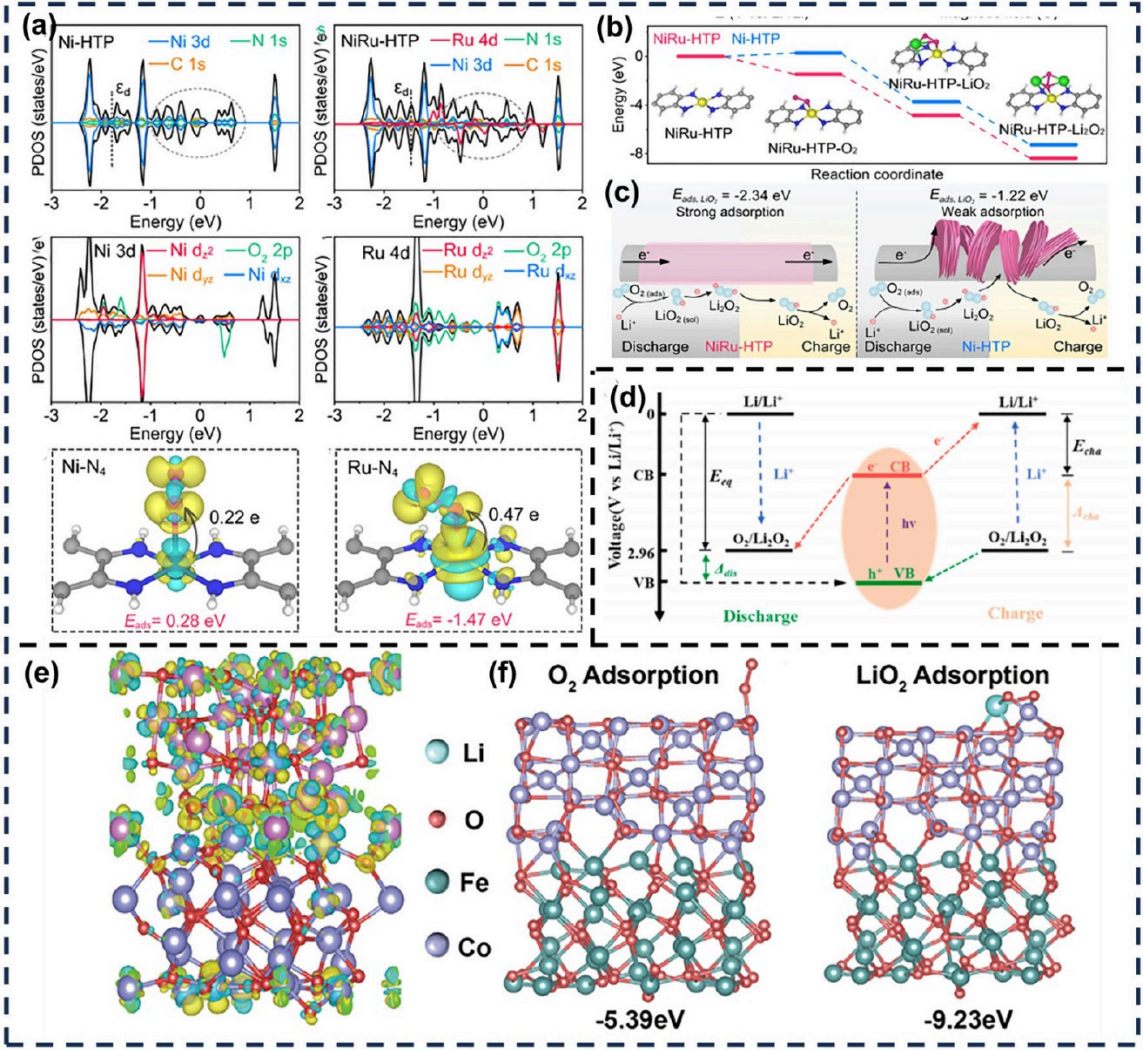
(a) Electronic structures of Ni-HTP and NiRu-HTP. (b) Free energy diagrams of oxygen reduction with optimized geometrical structures. (c) Proposed reaction mechanisms. *E*_ads, LiO2_ stands for the adsorption energy of LiO_2_. Reproduced with permission from ref 119. Copyright 2022 American Chemical Society. (d) Energy diagram and the reaction mechanism of the photoassisted LOBs. Reproduced with permission from ref 120. Copyright 2025 Elsevier. (e) Charge-density difference at the Co_3_O_4_/Fe_2_O_3_ interface. (f) Optimized adsorption of O_2_ and LiO_2_ on the Co_3_O_4_@Fe_2_O_3_. Reproduced with permission from ref 121. Copyright 2023 Wiley-VCH.

Defect engineering offers a novel pathway to modulate the catalytic activity of MOFs in LOBs. Methods such as high-energy particle irradiation or chemical etching can introduce vacancies and dislocations into the MOF crystal structure. These defects not only increase the number of active sites on the material surface but also disrupt the originally ordered electron cloud distribution, altering the electronic states and thereby promoting the adsorption and activation of reaction intermediates. Zhu et al.^120^ proposed a surfactant-assisted self-assembly strategy to successfully fabricate ultrathin 2D NH_2_-MIL-125 nanosheets with titanium vacancies (Figure 6d). Experimental studies revealed that the titanium vacancies form shallow defect states near the valence band, acting as hole traps that effectively suppress charge carrier recombination. Concurrently, the work function of the composite increased by 0.36 eV, facilitating vertical directional charge migration. Furthermore, the O_2_ adsorption energy significantly enhanced to – 0.92 eV, substantially higher than the – 0.04 eV of the pristine material, optimizing the oxygen activation pathway. This material achieved triple breakthroughs in photoassisted LOBs: the charge and discharge overpotential under illumination decreases to 0.60 V, significantly lower than the 1.20 V under dark conditions, while the discharge voltage reaches 2.99 V, approaching the theoretical value; the sheet-like Li_2_O_2_ product enhances conductivity, synergistically coupling with photogenerated electron–hole pairs to accelerate oxygen reduction and oxygen evolution reaction kinetics, respectively; cycling stability exceeds 160 cycles at 0.10 mA cm^–2^, far surpassing that of 3D bulk materials. In summary, this work efficiently utilizes light energy to reduce reaction barriers by shortening carrier migration paths via the 2D structure and tailoring the energy level structure through defect engineering, providing a new paradigm for developing high-efficiency solar-electric-chemical energy conversion batteries. The bimetallic synergistic strategy has proven effective in modifying MOF-based materials for LOBs. Zhao et al.^121^ developed an interfacial engineering strategy to construct Fe_2_O_3_ nanoparticles *in situ* on the surface of porous rod-like Co_3_O_4_, fabricating a Co_3_O_4_/Fe_2_O_3_ nanoheterostructured catalyst. Theoretical calculations reveal significant electron transfer and strong coupling effects at the heterointerface, which effectively modulate the electronic structure of Co_3_O_4_, lowering the O_2_ adsorption energy and LiO_2_ intermediate adsorption energy to – 5.39 and – 9.23 eV, respectively (Figure 6e and f). This electronic restructuring significantly reduces the oxygen reaction energy barrier, leading to decreased ORR and OER overpotentials of 0.17 and 0.30 V. In LOBs, the catalyst demonstrates outstanding overall performance, including a high discharge specific capacity (14847.8 mAh g^–1^), a low polarization voltage (1.08 V), excellent rate capability (retaining 5559.9 mAh g^–1^ at 1000 mA g^–1^), and stable cycling over 280 cycles. These superior properties originate from the accelerated Li_2_O_2_ formation and decomposition kinetics at the heterointerface, the 38% increased oxygen vacancy concentration, and the abundant active sites provided by the high-surface-area mesoporous structure, thereby collectively promoting reactant diffusion and charge transport.

#### 2.5.3 Structural Reinforcement

MOFs have garnered significant attention for LOBs due to their ultrahigh specific surface area, tunable porosity, structural designability, and inherent catalytic activity. However, these structural characteristics also present challenges: the high porosity renders the framework susceptible to collapse under the stress induced by Li_2_O_2_ volume changes; organic linkers are prone to decomposition in the strongly oxidizing and reductive environment, while metal centers exhibit insufficient stability and poor conductivity; furthermore, prolonged interaction with the electrolyte weakens coordination bonds. Consequently, targeted strategies are required to enhance their stability. The implementation of core–shell or hollow structures represents an effective approach to address these issues, with the primary advantages lying in preserving framework integrity and improving damage resistance. Specifically, the outer stable material (e.g., carbon layer or oxide shell) in a core–shell structure forms a physical barrier, effectively shielding the organic linkers in the MOF core from attack by strongly oxidizing and reductive species in the electrolyte, thereby mitigating the rupture of coordination bonds caused by linker oxidative degradation or hydrolysis. Simultaneously, the outer shell buffers the volume change pressure during Li_2_O_2_ formation and decomposition, preventing the core MOF from collapsing due to intense compression and consequently maintaining the integrity of the porous structure. Using ZIF-67 nanocubes as precursors, Qiu et al.^122^ employed synthesized two distinct hollow cobalt-based catalysts through different etching strategies: hollow Ni-ZIF was obtained via nickel nitrate etching, leveraging the Kirkendall effect, while hollow TA-ZIF was produced through ligand substitution with tannic acid. After subsequent ruthenium loading and calcination, the resulting Ni-ZIF@Ru-280 and TA-ZIF@Ru-280 catalysts retained their hollow cubic architectures. Notably, TA-ZIF@Ru-280 exhibited a robust hollow structure with a high specific surface area, abundant active sites, and an electronic structure enriched with oxygen vacancies, as confirmed by X-ray photoelectron spectroscopy (XPS) and electron paramagnetic resonance analyses. This defect engineering enhances oxygen species adsorption and reaction kinetics. Crucially, postcycling characterization revealed that TA-ZIF@Ru-280 maintained its structural integrity, whereas Ni-ZIF@Ru-280 underwent significant collapse, underscoring the superior structural stability of the TA-ZIF-derived material. This stability, combined with the catalytic efficacy of Ru nanoparticles, facilitates efficient and reversible Li_2_O_2_ formation and decomposition while suppressing side reactions. Zhu et al.^123^ developed a CoFe-LDH@CNTs composite by employing ZIF-67 dodecahedra as sacrificial templates, converting them into hollow CoFe-LDH nanocages assembled from ultrathin sheets via a one-step hydrothermal process while simultaneously anchoring them onto CNTs. This architecture integrates the hollow nanocages, which provide abundant active sites and ample internal space for accommodating Li_2_O_2_ discharge products, with an interconnected CNT network that prevents LDH agglomeration and significantly enhances overall electrical conductivity. Crucially, the heterostructure formed between CoFe-LDH and CNTs modulates the electronic configuration, as evidenced by XPS-confirmed coexistence of Co^2+^/Co^3+^ and Raman-indicated increased defect density. DFT calculations further reveal an optimized d-band center shift of Co active sites from – 2.25 to – 1.71 eV, positioning it closer to the Fermi level (Figure 7a). This electronic modulation strengthens adsorption toward O_2_ and Li_2_O_2_ while weakening LiO_2_ adsorption, thereby accelerating reaction kinetics and lowering energy barriers. Benefiting from this robust 3D hierarchical structure and optimized electronic states, the CoFe-LDH@CNTs cathode delivers a high initial discharge capacity of 32.8 Ah g^–1^ at 500 mA g^–1^ and maintains stable operation for 176 cycles under a limited capacity of 500 mAh g^–1^, significantly outperforming pristine CoFe-LDH and CNTs. The enhanced cycle life directly stems from the improved structural integrity and optimized electronic structure that promote efficient and reversible Li_2_O_2_ formation and decomposition.

**Figure 7:**
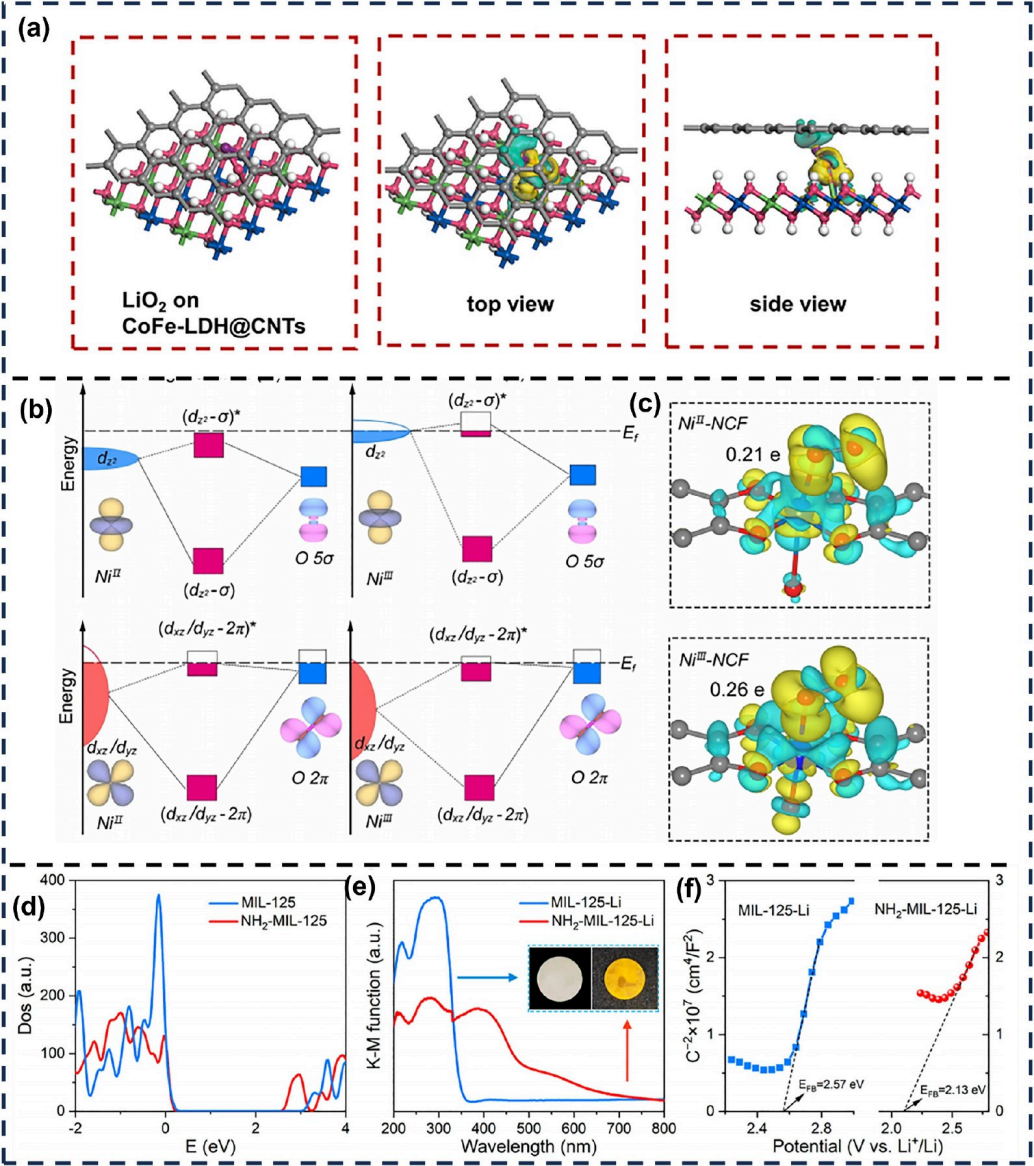
(a) The charge density difference plots of LiO_2_ on CoFe-LDH@CNTs. Reproduced with permission from ref 123. Copyright 2025 Elsevier. (b) Illustration of orbital interactions between adsorbed O_2_ (5σ and 2π) and Ni sites d_*z*_^2^ and d_*xz*_/d_*yz*_ in NiIII-NCF and NiII-NCF. (c) Differential charge densities of O_2_ adsorbed NiIII-NCF and NiII-NCF. Reproduced with permission from ref 125. Copyright 2024 Wiley-VCH. (d) TDOS and projected density of states of MIL-125 and NH_2_-MIL-125. (e) UV–vis spectra of MIL-125-Li and NH_2_-MIL-125-Li. (f) Mott–Schottky plots of MIL-125-Li and NH_2_-MIL-125-Li. Reproduced with permission from ref 126. Copyright 2023 American Chemical Society.

#### 2.5.4 Mass Transport Optimization

MOFs exhibit significant limitations in mass transport during electrochemical process of LOBs: their pores are susceptible to blockage by reaction products such as Li_2_O_2_, impeding reactant transport; the relatively uniform pore size distribution in some materials struggles to accommodate the dynamic morphological changes of products, often leading to localized mass transport inhomogeneities; additionally, suboptimal interfacial compatibility with electrolytes and poor wettability create substantial barriers to liquid-phase mass transport, constraining overall performance. The pore structure of MOFs governs the transport, catalysis, and stability of LOBs. Micropores offer high surface area but are easily blocked by Li_2_O_2_; mesopores balance catalysis and mass transport, alleviating product accumulation; macropores enhance permeation and relieve stress but lack active sites; hierarchical pores combine all advantages, providing high active site density, fast transport, and sufficient storage space, representing the optimal structural design. Addressing these challenges, pore engineering is an important strategy to improve the mass transfer of MOF. Wen et al.^124^ developed a porphyrin-based MOF (FeNi-TCPP MOF) via a dual-metal synergy strategy. Within this framework, Fe^3+^/Ni^2+^ coordinates with carboxyl groups of the TCPP linker, forming (Fe_2_Ni)­O­(COO)_6_ cluster nodes. Theoretical calculations reveal that Ni doping significantly elevates the energy level of the Fe 3d orbitals, enhances Ni–Fe orbital hybridization, and optimizes the electronic state distribution near the Fermi level, thereby promoting ligand-to-metal cluster charge transfer and driving efficient exciton dissociation into free charge carriers. Charge density distribution further confirms that Ni sites act as electron enrichment centers, lowering the activation energy barrier for O_2_. The well-defined channels constructed by these bimetal clusters facilitate accelerated O_2_/Li^+^ mass transport. Synergizing with photoinduced interfacial superoxide radicals, this enables uniform film-like deposition of the discharge product Li_2_O_2_ within pores (∼2 nm), effectively avoiding pore blockage by crystalline growth. This work simultaneously addresses critical challenges of exciton recombination, sluggish reaction kinetics, and product passivation through the synergistic optimization of electronic structure modulation and mass transport channels in the bimetallic MOF. The rapid interfacial conversion mechanism of the Li_2_O_2_ film establishes a new paradigm for high-efficiency photoassisted batteries.

In addition, the construction of a hierarchical pore structure can also effectively regulate mass transfer. Low-dimensional MOF architectures significantly enhance mass transport efficiency in LOBs. 2D layered MOFs typically feature interlayer spacings ranging from several to tens of nanometers, serving as rapid transport channels for ions and oxygen, thereby shortening the diffusion paths for Li^+^ and O_2_. Concurrently, the abundant active sites exposed on the nanosheet surfaces directly interface with the electrolyte, reducing mass transport resistance. In addition, the interlayer voids accommodate discharge product (L_2_O_2_) deposition, preventing pore blockage. Employing an oxidation modulation strategy, Lv et al.^125^ partially converted Ni^2+^ to Ni^3+^ in a nickel-catecholate framework (Ni^2+^-NCF), constructing a 2D conductive MOF nanowire array with bimetallic valence states (Ni^III^–NCF). Theoretical calculations reveal that Ni^3+^ sites significantly elevate the d-band center, enhance Ni–O covalency (manifested as increased overlap between Ni 3d and O 2p orbitals), and optimize the electronic state distribution. Charge difference analysis confirms a substantially stronger O_2_ adsorption energy on Ni^3+^ sites (−0.20 eV) compared to Ni^2+^ sites (−0.09 eV), facilitating electron injection from Ni d-orbitals into the O_2_ antibonding orbitals. Free energy diagrams further demonstrate an increased discharge voltage of 2.10 V for NiIII-NCF versus only 1.95 V for NiII-NCF (Figure 7b and c). This 2D nanowire array forms a hierarchical pore structure, where the well-defined channels (∼2 nm) synergistically promote O_2_ diffusion and Li^+^ transport, while the interwire gaps provide confined space for controlled discharge product growth. The strong adsorption of LiO_2_ intermediates on Ni^3+^ sites guides the uniform growth of Li_2_O_2_ as ∼ 50 nm-thick nanosheets within the nanowire gaps, effectively circumventing pore clogging. Moreover, spin-state regulation enhances electronic conductivity and structural stability, enabling stable cycling over 200 cycles with negligible capacity decay at 1000 mAh g^–1^. This work simultaneously addresses sluggish reaction kinetics and product passivation challenges through spin-state engineering that optimizes metal–oxygen covalency and mass transport channels, establishing a new paradigm of electronic structure modulation for high-stability LOBs.

Pore functionalization of MOFs offers an effective approach to enhance mass transport efficiency in LOBs. Incorporating polar functional groups (e.g., hydroxyl, amino) strengthens the adsorption and conduction capabilities for Li^+^ ions in the electrolyte, lowering the ionic migration barrier and thereby accelerating Li^+^ transport. Concurrently, functionalization modulates the surface charge distribution within the pores, mitigating excessive adsorption and accumulation of the discharge product Li_2_O_2_ and maintaining pore accessibility. This not only improves active site utilization and optimizes reaction kinetics but also significantly reduces overpotential induced by mass transport hysteresis. Employing a ligand functionalization strategy, Wang et al.^126^ introduced amino groups (−NH_2_) into the organic linkers of a Ti-based MOF (MIL-125), successfully fabricating the NH_2_-MIL-125-Li mixed conductor. Theoretical calculations demonstrate that amino modification markedly optimizes the electronic structure: the band gap narrows from 3.12 eV in the pristine MOF to 2.52 eV, broadening the visible-light absorption range and effectively suppressing the recombination of photogenerated electron–hole pairs. Crucially, the amino groups act as “bridges” that strengthen the interaction between Li^+^ ions and metal-oxo clusters, reducing the Li^+^ migration barrier to 0.15 eV (Figure 7d–f). MD dynamics simulations further reveal that the functionalized pores anchor TFSI^–^ anions via strong electrostatic adsorption, establishing a single-ion conductive environment while providing low-resistance diffusion pathways for Li^+^ ions. This results in a high Li^+^ transference number of 0.72 and a room-temperature ionic conductivity of 1.52 × 10^–4^ S cm^–1^. Moreover, photogenerated electrons and holes directly participate in the ORR/OER, reducing the charge–discharge voltage gap to 0.13 V and elevating the energy efficiency to 94.2%.

## 3 Pristine MOFs

Pristine MOFs offer an innovative paradigm for reconstructing the three-phase reaction interface in LOBs, leveraging their unique periodic porous architectures, precisely tunable active sites, and exceptional chemical stability. Pristine MOFs, owing to their highly tunable structures, well-ordered pore channels, and atomically dispersed active sites, are well-suited for mechanistic studies, interfacial regulation, and investigation of product growth behavior. As self-supported heterogeneous catalysts, MOFs direct the oriented growth of LiO_2_/Li_2_O_2_ via pore-size confinement effects while significantly lowering reaction energy barriers through open metal sites. Their highly ordered pore networks establish fast pathways for oxygen diffusion and ion transport, critical for mitigating mass transport limitations. Notably, recent investigations reveal that synergistic interactions between redox-active metal nodes (e.g., Fe^3+^, Co^2+^) and organic ligands enable dynamic regulation of reaction intermediates, overcoming the bottleneck of surface passivation in conventional carbon-based electrodes. The well-defined porous structure of MOFs precisely modulates lithium-ion flux via size-sieving effects and nanoconfinement, facilitating selective and accelerated Li^+^ migration. This dual functionality (structural confinement and catalytic activation) positions MOFs as transformative materials for addressing the sluggish kinetics and interfacial challenges inherent to LOB systems. This chapter systematically elucidates groundbreaking advances in pristine MOFs for cathode reaction catalysis, functional separator engineering, and electrolyte optimization in LOBs (Table 1), establishing a theoretical framework and technical pathways for developing advanced battery systems with simultaneously enhanced energy conversion efficiency and prolonged cycling stability.

**Table 1:** Summary of Pristine MOFs for LOBs

MOFs	Sample	Application	Discharge capacities (mAh g^–1^)	Cycle number	refs
Mn-MOF-74	Mn-MOF-74	Cathode	9420 (50 mA g^–1^)	30 (250 mA g^–1^)	^104^
Ni-MOF	Ni-MOF	Cathode	9000 (0.12 mA cm^–2^)	170 (250 mA cm^–2^)	^127^
Co-MOF-74	Co-MOF-74	Cathode	11350 (110 mA g^–1^)	8 (250 mA g^–1^)	^43^
NiMn-MOF	NiMn_0.05_-MOF	Cathode	28464 (100 mA g^–1^)	524 (100 mA g^–1^)	^128^
Ni-MOF	CoNi-MOF	Cathode	15784.2 (500 mA g^–1^)	127 (1000 mA g^–1^)	^129^
MnCo-MOF-74	MnCo-MOF-74	Cathode	11150 (200 mA g^–1^)	44 (200 mA g^–1^)	^130^
Mg-MOF-74	Tz-Mg-MOF-74	Cathode	7700 (50 mA g^–1^)	28 (200 mA g^–1^)	^131^
Co-MOF	Co-MOF-BA_0.1_	Cathode	14011 (100 mA g^–1^)	215 (1000 mA g^–1^)	^132^
2D Mn-MOF	Mn-MOF nanosheets	Cathode	9464 (100 mA g^–1^)	200 (100 mA g^–1^)	^133^
Mn-MOF-74	Mn-MOF-74- FcA	Cathode	15780 (100 mA g^–1^)	276 (200 mA g^–1^)	^45^
UIO-66	UIO-66-NH_2_	Cathode	12661 (100 mA g^–1^)	169 (200 mA g^–1^)	^134^
Mn-MOF	Mn-MOFs	Cathode	5579 (0.2 mA cm^–2^)	140 (500 mA cm^–2^)	^135^
HKUST-1	MOF-Based Separator	Separator	5000 (1000 mA g^–1^)	100 (1000 mA g^–1^)	^136^
UIO-66	UIO-66-(C00Li)_2_	Separator	–	410 (0.5 A g^–1^)	^137^
UIO-66	UIO-66-SO_3_Li	Separator	–	180 (0.5 mA cm^–2^)	^138^
HKUST-1	HKUST-1/GF	Separator	10314 (100 mA g^–1^)	180 (200 mA g^–1^)	^139^
UIO-67	UIO-67-Li	Electrolyte	6891 (100 mA g^–1^)	115 (500 mA g^–1^)	^112^
ZIF-62	LZGM	Electrolyte	13552 (100 mA g^–1^)	400 (100 mA g^–1^)	^140^
ZIF-67	LiIL-MOF	Electrolyte	3850 (100 mA g^–1^)	70 (100 mA g^–1^)	^141^

### 3.1 Cathode

The core advantages of MOF materials are their ultrahigh specific surface area and porosity, metal nodes and organic ligands that can be precisely controlled at the atomic level, and highly ordered pore structures. It is these characteristics that lay a solid foundation for its application in the field of LOBs and show great potential. In their seminal 2014 study, Wu et al.^104^ pioneered the systematic exploration of MOFs as multifunctional cathodes in LOBs (Figure 8a). This groundbreaking work employed five archetypal MOF architectures (Mn-MOF-74, Mg-MOF-74, Co-MOF-74, HKUST-1, and MOF-5) distinguished by their hierarchical pore topologies and metal-node electronic configurations. Electrochemical measurements demonstrate that the MOF/Super P composite cathode exhibits a remarkable enhancement in specific capacity compared to the pure carbon electrode. Notably, the Mn-MOF-74 composite material sets a record for specific discharge capacity of 9420 mAh g^–1^ under 1 atm O_2_, representing a 4-fold improvement over the control electrode (Figure 8b). Additionally, computational analyses reveal that the oxygen concentration within the pores of the high-performance catalyst Mn-MOF-74 is 18-fold higher than that of pure oxygen at 1 atm. This nanoconfined oxygen densification (arising from the compact packing of O_2_ molecules within MOF pores) substantially increases the available oxygen content in LOB systems without compromising electrode volume or mass, directly contributing to the exceptional discharge capacity of LOB. To verify whether the increase in capacity is attributable to side reactions, the authors characterized the gas products during the battery charging process with the aid of differential electrochemical mass spectrometry (DEMS) measurements. The DEMS results indicated that the charging product is nearly pure oxygen, suggesting that the MOF does not catalyze side reactions in the battery, including the decomposition of the electrolyte. Subsequently, Hu et al.^127^ innovatively synthesized a nickel-based MOF through solvothermal crystallization, featuring a hierarchical pore architecture with interconnected micropores (5–8 Å) and mesopores (∼50 nm). This bimodal porosity system synergistically enhances O_2_/Li^+^ dual-phase transport kinetics while providing exceptional surface accessibility for electrolyte infiltration (Figure 8c). Electrochemical characterization reveals an extraordinary specific capacity of 9000 mAh g^–1^ at 0.12 mA cm^–2^ (Figure 8d), surpassing conventional carbon cathodes by 320%. Remarkably, the electrode maintains minimal polarization (*ΔE* < 0.25 V) over 170 cycles, demonstrating unprecedented cycling stability in aprotic LOBs. Through X-ray Diffraction (XRD) measurements (Figure 8e), it is evident that the characteristic peaks of Ni-MOFs remain unaltered after the initial discharge, signifying their stability under discharging conditions. However, certain alterations are also observed in the XRD patterns. For the discharged cathode, aside from the diffraction peaks of Ni-MOF, C, and Ni foam, the XRD pattern solely indicated the formation of Li_2_O_2_, with no other discharge byproducts such as LiOH detected. Moreover, the Li_2_O_2_ peak disappears after charging, signifying that the product decomposes completely during the charging process. This observation provides strong evidence for the structural reversibility of the material, underscoring its potential for enabling fully reversible LOBs chemistry.

**Figure 8:**
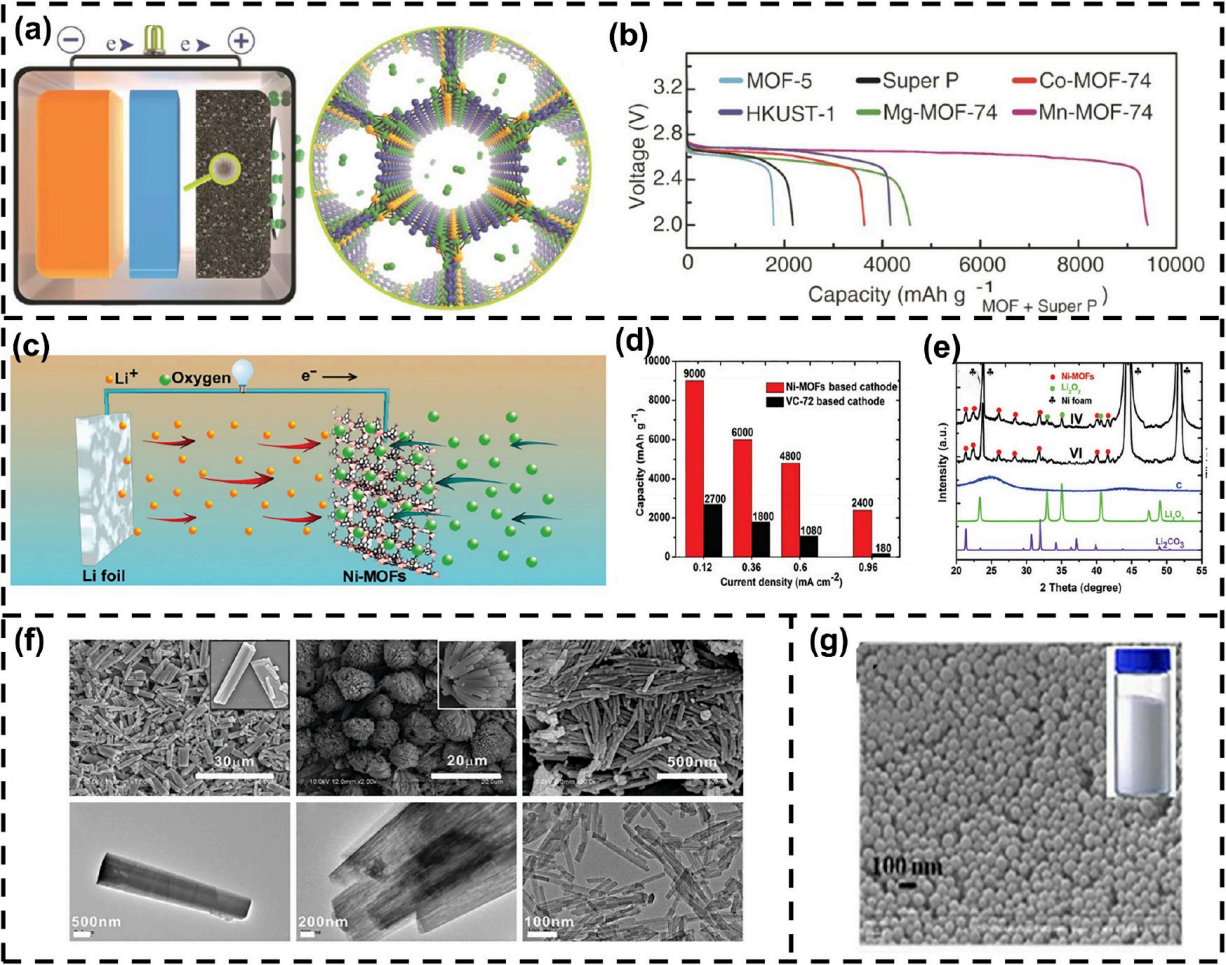
(a) Schematic illustration of an LOBs cell using MOF-Super P composite as the O_2_ electrode. Oxygen molecules’ relative sizes are reduced for clarity. (b) Discharge profiles of the LOB using MOF-Super P composites or Super P only under O_2_ atmosphere with a current of 50 mA g^–1^ at room temperature Reproduced with permission from ref 104. Copyright 2014 Wiley-Vch. (c) Schematic diagram of Ni-MOFs-based LOB. (d) Comparison of the discharge capacity of Ni-MOFs-based LOBs and the control group at different current densities. (e) XRD patterns of Ni-MOFs-based cathode at different discharge/charge states Reproduced with permission from ref 127. Copyright 2015 Royal Society of Chemistry. (f) SEM and TEM images of Co-MOF-74 Reproduced with permission from ref 43. Copyright 2017 Royal Society of Chemistry. (g) SEM images and particle size distribution of Dy-BTC Reproduced with permission from ref 44. Copyright 2020 Copyright Royal Society of Chemistry.

Since the particle size and morphology of cathode catalysts exert a significant influence on reaction kinetics, nanoengineering and morphology modification represent important strategies to improve the performance of MOF-based cathode for LOBs. Reducing crystal dimensions not only preserves the intrinsic advantages of MOFs but also shortens the diffusion pathways for Li^+^ ions while enhancing the accessibility of active sites for O_2_ molecule adsorption and surface reactions. These structural optimizations collectively endow MOFs with the desired electrochemical properties, enabling efficient charge–discharge kinetics and improved energy utilization.^142^ Yan et al.^43^ demonstrated the rational synthesis of Co-MOF-74 architectures with precisely modulated nanoscale dimensions through strategic solvent engineering (Figure 8f). By systematically modulating the N, N-dimethylformamide/H_2_O solvent ratio and introducing salicylic acid as a crystallographic modulator, the researchers successfully reduced the MOF crystallites to nanoscale dimensions, averaging 20 nm in diameter. This dimensional optimization, coupled with the formation of well-defined nanofibrous architectures, synergistically enhances the accessibility of catalytically active sites while establishing shortened ion diffusion pathways. The resultant nanoscale Co-MOF-74 derivatives exhibit exceptional electrochemical performance, delivering a remarkable specific discharge capacity of 11350 mAh g^–1^ at 100 mA g^–1^. This study highlights the effectiveness of morphological engineering in MOF-based systems, where the controlled fabrication of nanorod architectures emerges as a promising structural engineering strategy for establishing expedited charge transport networks and enhancing the activity of oxygen-related reactants. Beyond conventional transition metal-based MOFs, emerging nanosized lanthanide MOF architectures exploiting their distinctive orbital electronic configurations have recently emerged as promising oxygen cathodes for LOB systems.^142^ Pioneering work by Liu et al.^44^ demonstrated the strategic engineering of constructed from Dy^3+^ ions and 1,3,5-benzenetricarboxylic acid (Dy-BTC) MOF nanocrystals with precisely controlled crystallite dimensions averaging 60 nm (Figure 8g). The engineered Dy-BTC nanospheres exhibit a synergistic combination of unsaturated metal coordination sites, intrinsic microporosity, enhanced specific surface area, and hierarchical porosity architectures. This unique structural ensemble facilitates substantial oxygen molecule enrichment at the cathode-electrolyte interface while optimizing interfacial contact between reactive species and catalytically active centers, ultimately enabling exceptional LOBs performance. Remarkably, the Dy-BTC nanoarchitecture delivers a reversible specific discharge capacity of 7618 mAh g^–1^ at 50 mA g^–1^. More importantly, it maintains 1000 mAh g^–1^ with zero capacity decay after 76 cycles even under an elevated current density of 200 mA g^–1^, demonstrating unprecedented electrochemical stability in LOB systems.

As conventional MOF catalysts often suffer from weak orbital coupling with oxygen species due to unfavorable metal site alignment, selective substitution of metallic elements enables precise modulation of MOF electronic structures to enhance catalytic functionality. Specifically, the rational design paradigm of modulating 3D electronic configurations in MOF-based catalysts through secondary transition metal incorporation has emerged as a transformative strategy for enhancing electrocatalytic activity toward oxygen electrode reactions in LOBs. Wang et al.^128^ pioneered a bimetallic coupling strategy to construct Ni/Mn heterometallic–organic frameworks (Ni/Mn-MOFs). Combining experimental results with DFT calculations (Figure 9a), it is revealed that Mn doping induces 3d electron rearrangement at Ni sites, optimizing the occupation of Ni e_3_ orbitals and enhancing the hybridization between Ni-3d and O-2p orbitals. Meanwhile, the electronic coupling between Ni and Mn strengthens the covalency of Ni–O bonds, thereby significantly improving the adsorption capacity for LiO_2_ intermediates (Figure 9b). By contrast, the LiO_2_ intermediate cannot be strongly fixed by Ni sites of Ni-MOF. Therefore, NiMn 0.05-MOF displays much stronger chemical adsorption toward LiO_2_ intermediates with the enhanced absorption energy (Eads) of – 1.563 eV, outperforming the adsorption of LiO_2_ on Ni-MOFs (−1.039 eV) (Figure 9c). The introduction of Mn modulates the electronic structure of Ni, reducing the energy barriers for oxygen electrode reactions: the overpotentials for OER and ORR are decreased to 0.09 and 0.37 V, respectively. Additionally, a surface work function reduction to 8.08 eV enhances electron transport efficiency, contributing to the overall improvement in catalytic performance (Figure 9d). Electrochemical evaluations demonstrate that LOBs equipped with NiMn_0.05_-MOF electrocatalysts achieve benchmark-setting performance metrics, exhibiting an ultrahigh specific capacity of 28464 mAh g^–1^ and exceptional cyclability sustaining 524 cycles at 100 mA g^–1^. This work addresses the bottlenecks of poor electrical conductivity and inadequate catalytic activity in traditional MOFs by optimizing orbital interactions at metal sites, thereby providing new insights for the design of high-efficiency LOBs electrode materials. Zhao et al.^129^ developed a precisely engineered nanoflower-type Ni-MOF architecture through innovative morphological control and strategic heterometallic doping with Co ions, achieving a remarkable 165% expansion in pore dimensions (Figure 9e). This multiscale structural engineering induces two critical modifications: (1) The introduced Co active sites strategically optimize the adsorption strength toward LiO_2_ intermediates, enabling dual-pathway (solution-mediated and surface-assisted) formation of Li_2_O_2_ discharge products; (2) The expanded pore architecture establishes 3D ion highways that facilitate rapid Li^+^/O_2_ diffusion kinetics. These synergistic effects culminate in exceptional electrochemical performance, delivering a groundbreaking discharge capacity of 15784.2 mAh g^–1^. Moreover, the cycling performances of the Ni-MOF and CoNi-MOF electrodes were tested by varying the current density, and the CoNi-MOF electrode can maintain 127cycles at a current density of 1000 mA g^–1^ whereas 93 cycles for the Ni-MOF (Figure 9f). The hierarchically porous framework effectively mitigates electrolyte penetration barriers and ion transport bottlenecks, while the Co dopants significantly enhance OER kinetics through accelerated Li_2_O_2_ decomposition pathways.

**Figure 9:**
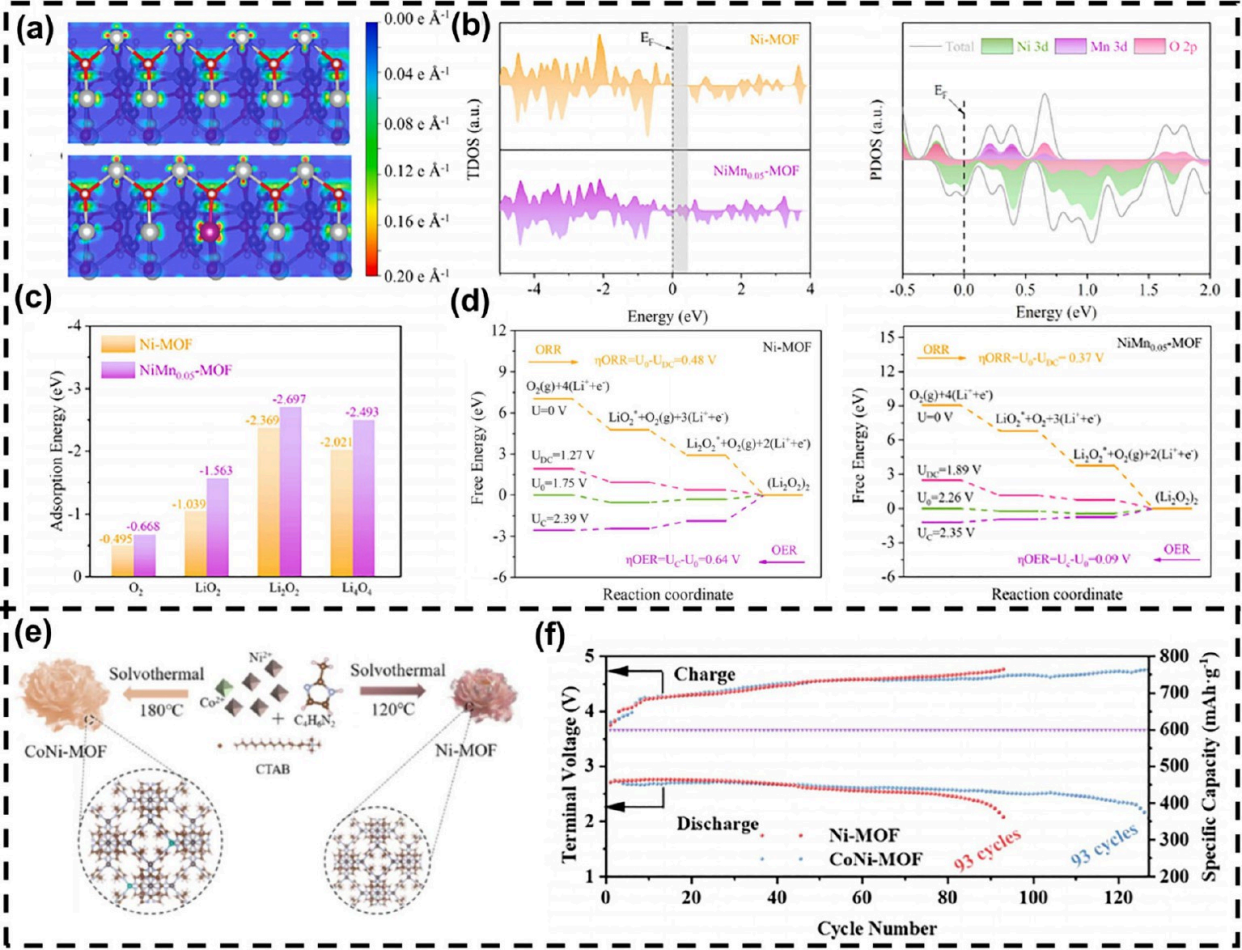
(a–d) Theoretical calculation of Ni-MOF and NiMn_0.05_-MOF. Reproduced with permission from ref 128. Copyright 2023 Elsevier. (e) Schematic synthesis of the Ni-MOF and CoNi-MOF. (f) Cycling performances of the Ni-MOF and CoNi-MOF under a specific capacity limit of 600 mAh g^–1^ at a current of 1000 mA g^–1^. Reproduced with permission from ref 129 Copyright 2024 Royal Society of Chemistry.

Beyond metal nodes engineering, organic ligands modification serves as another critical branch of MOF microstructural regulation, offering an effective strategy for boosting the electrochemical performance of LOBs. Precise modulation of organic ligands enables effective tailoring of the pore structure, chemical composition, coordination environment, and defect chemistry of MOFs, thereby synergistically optimizing the mass transport kinetics and electrocatalytic activity in LOBs.^142^ Wang et al.^143^ demonstrated an innovative ligand-engineering strategy to construct a hierarchically porous electrocatalyst by partially substituting 1,3,5-benzenetricarboxylate (BTC) ligands with isophthalic acid in microporous Cu-BTC frameworks, yielding mesoporous MCu-BTC with coordinatively unsaturated Cu sites (Figure 10a). Subsequent cobalt incorporation via impregnation generates Co/MCu-BTC hybrids, where the copper nodes formed mesopores through reduced carboxylate coordination, while cobalt species synergistically enhanced bifunctional catalytic activity. When deployed in LOBs, the engineered mesopores facilitated efficient electrolyte infiltration and mass transport, coupled with expanded storage capacity for insoluble Li_2_O_2_ discharge products, achieving a remarkable discharge capacity of approximately 7000 mAh·g^–1^. The unsaturated Cu centers markedly improved electronic conductivity and reactant affinity, effectively reducing overpotentials for both oxygen reduction and evolution reactions, with cobalt further refining reaction kinetics through optimized charge transfer pathways. Electrochemical evaluations revealed exceptional cyclability, as Co/MCu-BTC maintains stable operation over 55 cycles with a narrow voltage gap of 1.46 V, significantly outperforming conventional microporous Cu-BTC analogs. Computational simulations corroborate that mesopores alleviate pore blockage from discharge products while unsaturated metal sites promote intermediate adsorption and charge redistribution. This work establishes a paradigm for tailoring MOF architectures through pore engineering and coordination modulation, offering a transformative design blueprint for high-energy-density, durable LOBs. Li et al. demonstrated the viability of engineering MOF-based cathodes with redox-active organic motifs for advanced LOBs through molecular-scale precision engineering.^131^ The research team implemented a ligand functionalization strategy that covalently immobilizes tetrazine (Tz) RMs within the nanoporous architecture of MOFs, creating a stable Tz-Mg-MOF-74 cathode architecture that synergistically integrates molecular redox activity with framework stability (Figure 10b). This innovative design addresses the critical challenge of mediator dissolution in organic electrolytes through molecular confinement enabled by π-orbital interactions between the tetrazine molecules and the MOF’s densely packed organic linkers. Mechanistic investigations reveal that the hierarchical pore structure accelerates reaction kinetics via three complementary mechanisms: enhanced interfacial electron transfer through dynamic π-electron coupling of redox-active Tz groups, optimized mass transport through mesoscopic channels, and stabilization of reaction intermediates via Lewis acid–base interactions at magnesium nodes. Research indicates that, in comparison to nonfunctionalized MOFs and other reported MOF-based cathode materials, the designed Tz-Mg-MOF-74 with a high density of redox-active Tz sites exhibits superior discharge capacity and a lower overpotential. This approach effectively integrates the electrochemical activities of MOFs and redox molecules, offering an ideal platform for targeted high-performance LOB cathodes and opening up a novel avenue for the design of materials related to energy storage and conversion. Subsequently, Wang et al.^132^ employed a molecular cleavage strategy, optimizing the local coordination structure of Co-MOF by introducing the linker-deficient benzoic acid (BA) (Figure 10c). Partial substitution of terephthalic acid ligands with BA ligands in the constructed Co-MOF-BA induces lattice distortion, generating defect states that significantly promote electron transfer between the valence and conduction bands of Co-MOF-BA. This process facilitates electron exchange between the coordination-unsaturated Co sites (CUCSs) of Co-MOF-BA and adsorbed oxygen species. Additionally, the asymmetric coordination of CUCSs with oxygen-containing ligands triggers energy reconfiguration and electron localization at CUCSs, enhancing energy-level alignment with adsorbed oxygen and reducing the filling degree of antibonding states in Co–O bonds, thus accelerating the kinetics of oxygen electrode reactions on Co-MOF-BA. Comparative analysis between Co-MOF-BA_0.1_ and Co-MOF-BA_0.2_ reveals that Co-MOF-BA_0.1_ exhibits optimal oxygen adsorption, enabling a solvation-mediated growth model for the discharge product Li_2_O_2_. The resulting nanosheet morphology of Li_2_O_2_ facilitates electron and mass transport, alleviating active site blockage and further improving the reversibility of oxygen electrode reactions. As a result, the LOB using the Co-MOF-BA_0.1_ electrode demonstrated outstanding electrochemical performance with low overpotential and high cycle stability (Figure 10d). Lewis basic moieties have emerged as critical modulators for tailoring the electronic characteristics of Lewis acidic centers in electrocatalysts, thereby enhancing the electrochemical performance of LOBs. Despite their widespread application of Lewis bases, the mechanistic underpinnings of their role in mediating electrocatalytic processes within LOBs remain insufficiently understood. Yuan et al.^134^ addressed this knowledge gap through the strategic construction of a zirconium-based MOF (termed UIO-66-NH_2_) incorporating precisely engineered nucleophilic centers. This seminal work elucidates the pivotal role of Lewis basic sites: (1) The electron-donating characteristics of amino groups facilitate activation of oxygen species and intermediates, significantly accelerating oxygen redox kinetics; (2) During discharge, lithium-ion coordination at basic sites induces electronic structure modulation of the MOF framework, attenuating LiO_2_ adsorption energies to stabilize soluble intermediates that enable solution-mediated growth of toroidal Li_2_O_2_ architectures. The optimized UIO-66-NH_2_ cathode demonstrates exceptional electrochemical metrics, achieving a discharge capacity of 12661 mAh g^–1^ with a low overpotential of 0.87 V, coupled with extended cyclability over 169 cycles. This fundamental understanding not only deciphers the direct participation of nucleophilic centers in oxygen electrochemistry but also establishes a design paradigm for developing bifunctional acid–base catalytic systems in next-generation metal–air battery technologies.

**Figure 10:**
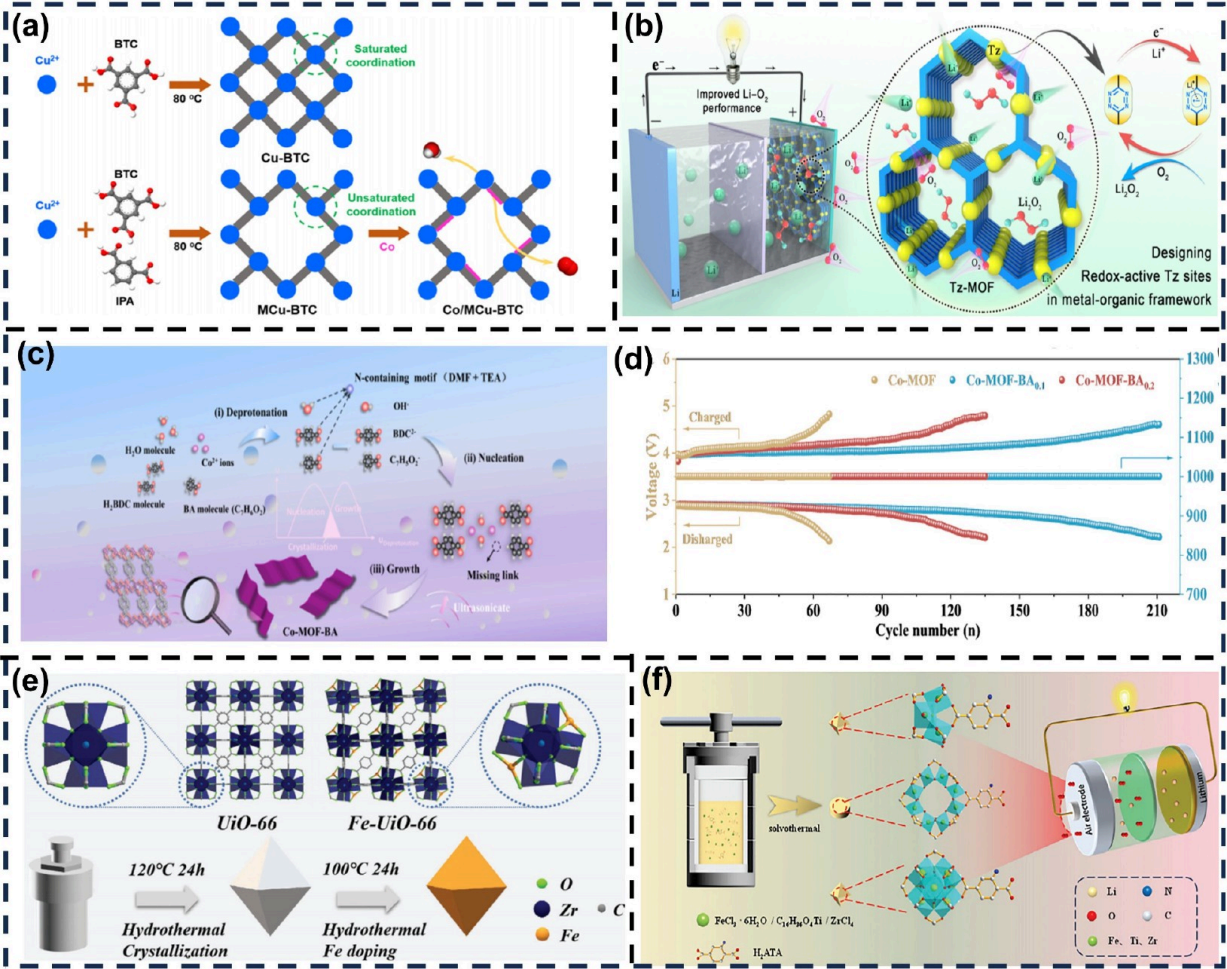
(a) Scheme of a ligand-defect approach to engineer mesopores and unsaturated coordination in a primarily microporous Cu-BTC. Reproduced with permission from ref 143. Copyright 2021. American Chemical Society. (b) Demonstration of the advantages of an LOB equipped with a MOF-based cathode via the functionalization of redox-active Tz. Reproduced with permission from ref 131. Available under a CC-BY 4.0 license. Copyright 2021 Chinese Chemical Society Li et al. (c) Schematic illustration of the synthesis process of Co-MOF-BA. (d) Cyclic stability of Co-MOF, Co-MOF-BA_0.1,_ and Co-MOF-BA_0.2_ electrodes. Reproduced with permission from ref 132. Copyright 2023 Elsevier. (e) Illustration of the preparation process and the proposed structure for Fe-UiO-66. Reproduced with permission from ref 114. Copyright 2024 Wiley-VCH. (f) Synthesis process and structural diagram of Fe/Ti/Zr MOF. Reproduced with permission from ref 145. Copyright 2024 Wiley-VCH.

The intrinsically low electronic conductivity (σ<10^–9^ S cm^–1^) characteristic of conventional MOFs presents a fundamental limitation in electron transport kinetics, severely constraining their electrochemical performance. Recent breakthroughs in materials engineering have yielded a revolutionary class of conductive MOFs with remarkable charge transport properties. The direct implementation of these advanced conductive MOFs in LOB systems promises to overcome historical conductivity barriers while unlocking their latent catalytic potential through enhanced electron delocalization pathways. A groundbreaking study by Majidi et al.^144^ engineered Cu-THQ, a conductive MOF architecture, which was exfoliated into ultrathin 2D nanosheets for deployment as catalytic cathodes in LOBs. This nanostructured configuration facilitates the growth of nanocrystalline Li_2_O_2_ products with amorphous domains, establishing an innovative growth platform that enables continuous expansion of discharge products while maintaining rapid discharge kinetics at elevated current densities. Remarkably, the amorphous Li_2_O_2_ phase exhibits exceptional synergistic compatibility with RMs in the electrolyte matrix. The electronically conductive nature of this unique discharge product allows RMs to engage in direct interfacial charge transfer at Li_2_O_2_ surfaces rather than being restricted to catalyst edge sites, followed by efficient mass transport through the electrolyte phase. DFT unveils that this distinctive Li_2_O_2_ configuration originates from pronounced electronic interaction between Cu centers and O_2_. This fundamental understanding demonstrates that precisely engineering the coordination architecture of metallic nodes in MOFs can effectively tailor the morphological evolution and crystallographic characteristics of discharge products, consequently governing the overall electrochemical performance metrics. Yuan et al.^133^ employed a facile ultrasonication method to synthesize ultrathin 2D MOFs, specifically 2D Mn-MOF, Co-MOF, and Ni-MOF. These materials are composed of metal ions (Mn^2+^, Co^2+^, Ni^2+^) coordinated with terephthalic acid ligands, forming a layered structure with inherently open active sites. The material properties influence electronic conductivity primarily through their semiconducting nature: DFT calculations and experimental measurements reveal that 2D Co-MOF exhibits the highest intrinsic electronic conductivity due to its smallest band gap, with conductivity increasing at elevated temperatures. However, at room temperature, all three 2D MOFs show comparable conductivity values, indicating that conductivity alone is not the sole determinant of battery performance. Electronic conductivity directly affects the efficiency of electron transfer during battery reactions; good conductivity facilitates faster kinetics for both the ORR and OER, thereby enhancing rate capability and reversibility.

Recent years have witnessed groundbreaking advancements in the application of photoactive MOFs for photoelectrochemical LOBs. Through meticulous structural engineering, these crystalline porous materials demonstrate exceptional charge carrier separation efficiency and catalytic conversion capabilities. Yang et al.^114^ developed a bimetallic Fe-UiO-66 architecture via hydrothermal synthesis (Figure 10e), establishing it as a high-performance photoelectrode for light-assisted LOBs. The strategic incorporation of iron centers not only broadens the visible-light absorption spectrum of UiO-66 but also creates active sites that synergistically facilitate both oxygen reduction and evolution reactions. During the discharge phase under illumination, photogenerated electrons activate oxygen molecules to generate superoxide radicals, thereby accelerating oxygen reduction kinetics and promoting the formation of membrane-like Li_2_O_2_ products. The evolution pathway of intermediates fundamentally depends on the interaction strength between electrolyte solvents and LiO_2_ species. Through advanced *in situ* electron paramagnetic resonance spectroscopy analysis, the authors elucidated the cascade electron transfer mechanism where electrons sequentially migrate from Fe^III^ centers to Zr–O clusters before ultimately transferring to oxygen species, with predominant oxygen reduction proceeding through the superoxide radical pathway. Complementary DFT simulations reveal preferential oxygen adsorption at Fe sites within the Fe-UiO-66 framework, where subsequent lithium-ion coordination facilitates the transformation from LiO_2_ to Li_2_O_2_. Experimental validation demonstrates that Fe-UiO-66-based photoelectrodes achieve remarkable performance metrics, including a significantly reduced overpotential of 0.14 V under illumination and outstanding cycling stability over 500 cycles. This system exhibits enhanced reaction kinetics, minimized polarization, and superior reversibility compared to conventional counterparts. In a pivotal advancement within the same year, Tao et al.^145^ pioneered the rational design of MOFs incorporating iron, titanium, or zirconium metallic clusters as photoelectrodes for light-assisted LOBs (Figure 10f). The Fe-MOF architecture demonstrates remarkable dual excitation pathways under illumination coupled with exceptional charge separation efficiency, synergistically enhancing O_2_/Li_2_O_2_ activation dynamics while boosting catalytic performance for both oxygen reduction and evolution reactions. Distinct from conventional inorganic semiconductor crystals, this nanoporous framework exhibits an enlarged specific surface area and superior oxygen adsorption capacity. Consequently, LOBs employing Fe-MOF cathodes demonstrate exceptional performance metrics, including enhanced specific capacity, an ultralow overpotential of 0.22 V at 0.05 mA cm^–2^, and sustained cycling stability over 195 cycles under continuous illumination.

### 3.2 Separator

Beyond their conventional deployment as cathode materials, the intrinsically ordered porous architecture of MOFs renders them particularly suitable for advanced separator modifications in next-generation battery systems. Through precise control over synthesis protocols, researchers can engineer MOF-based separator coatings with structural integrity and defect-minimized interfaces. The tunable pore dimensions characteristic of MOFs enable selective ion sieving capabilities that effectively mitigate critical failure mechanisms, including lithium dendrite proliferation and shuttle phenomena between electrodes. This strategic implementation of MOF-engineered separators establishes a materials-centric approach to overcoming long-standing challenges in battery durability and electrochemical stability.^142^

2. Employ AI-driven closed-loop frameworks with multi-objective optimization to accelerate breakthroughs of MOFs in energy storage and beyond.

RMs demonstrate exceptional capability in orchestrating the formation and decomposition kinetics of Li_2_O_2_ discharge products while effectively mitigating charge overpotentials. Nevertheless, the operational longevity of RM-enhanced systems is fundamentally compromised by the inherent shuttle, the uncontrolled migration of oxidized RM species through separator membranes to lithium metal anodes. This detrimental process triggers parasitic side reactions that not only deplete active mediators but also induce catastrophic anode degradation manifested through metallic corrosion, structural pulverization, and dendritic proliferation. The resultant dual depletion of both redox-active species and lithium inventory creates a self-amplifying degradation loop that progressively diminishes catalytic efficacy and undermines overall battery cyclability. Pioneering work by Qiao et al.^136^ engineered a hierarchical HKUST-1-integrated composite separator comprising PVDF-HFP and Celgard substrates through advanced *in situ* crystallization protocols. This architecturally refined membrane exhibits dual functionality: facilitating selective Li^+^ conduction while leveraging MOF-based precise molecular sieving capabilities to block RMs migration toward the anode, thereby effectively suppressing lithium dendrite formation and parasitic redox shuttle effects (Figure 11a). Compared to conventional Celgard and glass fiber separators, the MOF-optimized membrane demonstrates exceptional electrolyte uptake capacity and interfacial stability. Batteries incorporating this advanced separator achieve remarkable electrochemical performance metrics, including a discharge capacity of 5000 mAh g^–1^ with constrained polarization at 1000 mA g^–1^, along with extended cyclability over 100 cycles under capacity-limited conditions. This technological breakthrough establishes a materials engineering paradigm for developing high-energy flexible pouch-type LOBs. Wang et al.^137^ proposed a MOF-gel separator to inhibit the shuttle of RMs (Figure 11b and c). Unlike conventional MOF nanoparticle-modified membranes, the MOF-gel separator demonstrates exceptional microstructural homogeneity and defect-free interfaces, achieved through advanced gelation protocols. When employing ruthenium acetylacetonate (Ru­(acac)_3_) as the RM, the MOF-gel/Celgard composite architecture exhibits remarkable shuttle suppression efficiency, effectively confining mediator species within the cathode compartment while preventing anode-side parasitic reactions. This breakthrough enables unprecedented electrocatalytic longevity with extended cyclability exceeding 410 cycles. Under demanding operational conditions (2000 mAh g^–1^ reversible capacity at 1.0 A g^–1^), conventional Celgard-based batteries exhibit rapid performance degradation: charge potentials escalate beyond 4.5 V within merely 5 cycles, accompanied by significant voltage hysteresis. In stark contrast, MOF-gel integrated battery maintains stable electrochemical profiles with terminal voltages below 4.5 V even after 20 cycles. This phenomenon directly correlates with the superior molecular filtration efficiency (98.7% RM retention) and interfacial stabilization capability of MOF-gel. The fundamental insights from this work provide critical design guidelines for next-generation separator technologies and advanced mediator development in metal–air battery systems.

**Figure 11:**
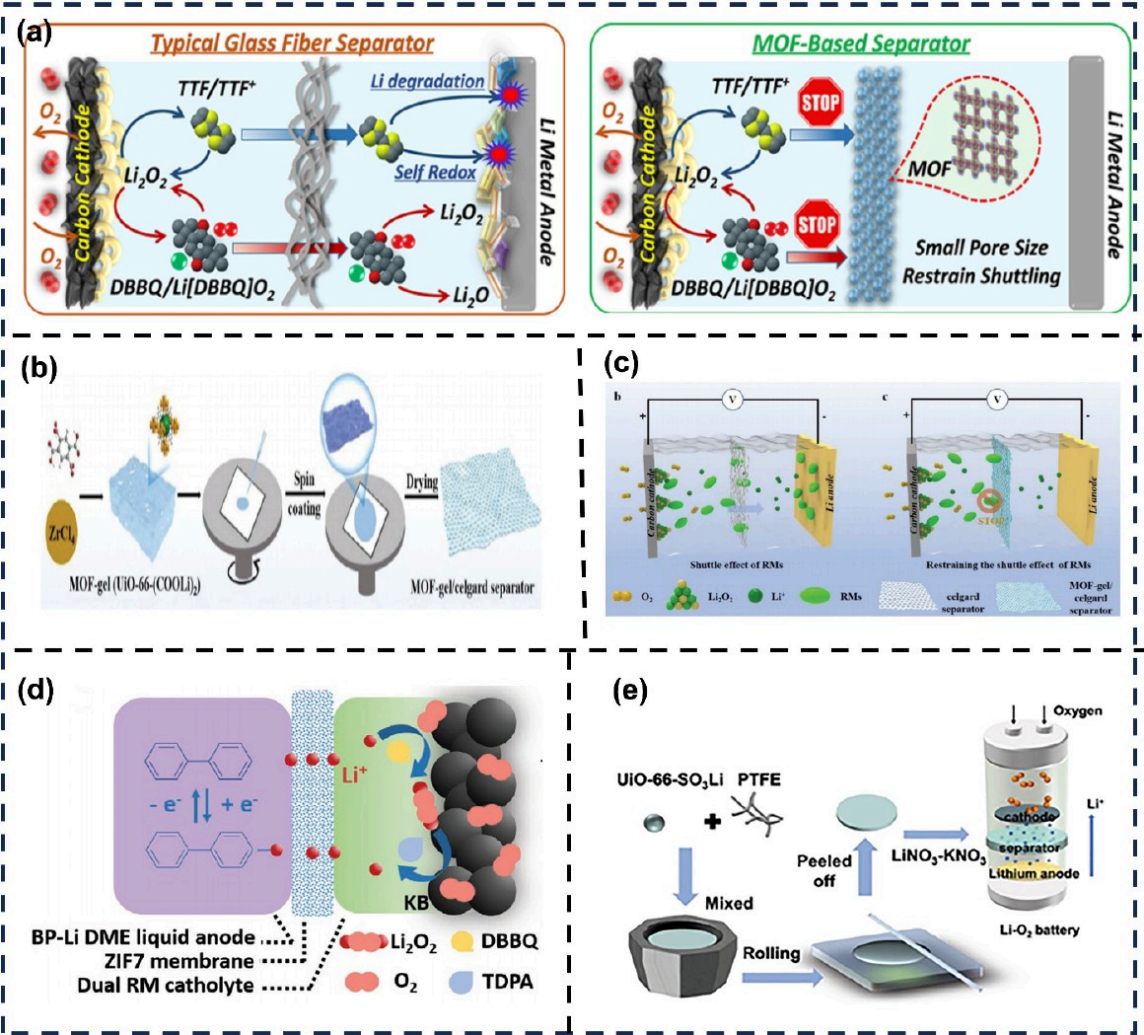
(a) Schematic images of a typical glass fiber and MOF-based separator. Reproduced with permission from ref 136. Copyright 2018 American Chemical Society. (b) Scheme of the synthesis of MOF-gel and the subsequent film fabrication process through the spin-coating method. (c) A Celgard separator and an MOF-gel/Celgard separator acting as an ionic sieve to inhibit the shuttling of RMs. Reproduced with permission from ref 137. Copyright 2024 Wiley-VCH. (d) The schematic illustration of the organic O_2_ battery with Bp-Li liquid anode, dual RM-assisted KB cathode, and ZIF7 membrane as a separator. Reproduced with permission from ref 110. Copyright 2020 Wiley-VCH. (e) Schematic of the synthesis of the MOF membrane and the elevated temperature LOB. Reproduced with permission from ref 138. Available under a CC-BY 4.0 license. Copyright 2023 Wiley-VCH Qiu et al.

In addition to mitigating the shuttle effect of RMs, MOF-modified separators have also been employed to construct novel LOBs with a liquid anode. Deng et al.^110^ fabricated a ZIF-7 separator via a coating method, integrating it with a lithium-based organic liquid anode (Bp-Li) featuring high ionic conductivity, low operating potential, and high safety, along with an oxygen cathode, to assemble organic LOBs (Figure 11d). The ZIF-7 separator not only physically separates the liquid anode but also restricts the crossover of RMs to the anode side, effectively suppressing cross-talk effects. The 3D porous structure of the ZIF-7 separator enables rapid ion transport, while a SEI-like layer on its surface promotes the formation of a more stable interface with the Bp-Li anode.^146^

The development of molten salt electrolyte-based LOBs operating at elevated temperatures has garnered significant attention due to their inherent capability to achieve accelerated reaction kinetics. Compared with room-temperature LOBs, molten-salt-based LOBs impose more stringent requirements on the high-temperature stability, ionic conductivity, and molecular sieving capability of separators. Qiu et al.^138^ pioneered a groundbreaking separator design utilizing lithiated zirconium-based MOFs with subnanometer pore apertures (6-Å UiO-66-SO_3_Li), specifically engineered for high-temperature LOBs operation (Figure 11e). Through innovative doctor-blade processing, the researchers achieved defect-free membrane fabrication with zero material waste. The optimized MOF-based separator enables stable LOB operation at 160 °C, achieving an areal capacity of 5.1 mAh cm^–2^ with remarkably low overpotential (40 mV at 0.1 mA cm^–2^), while demonstrating exceptional electrochemical stability through 180 cycles with 99.9% Coulombic efficiency at 0.5 mA cm^–2^. Crucially, the lithiated MOF architecture establishes dual functionality: creating efficient Li^+^ conduction pathways through sulfonic acid group coordination while physically constraining discharge product migration via molecular sieving effects. This materials engineering breakthrough addresses critical challenges in high-temperature battery systems, thereby establishing a transformative pathway toward scalable LOBs implementations.

### 3.3 Electrolyte

Conventional organic liquid electrolytes for LOBs typically encounter volatilization, leakage, insufficient stability, and poor compatibility, posing critical challenges to the practical implementation of LOBs. In addition, side reactions arising from irreversible consumption of electrolytes and inevitable collapse of SEI lead to diminished Coulombic efficiency, while “dead Li” originating from the formation and root-dissolution of lithium dendrites during repeated plating and stripping causes degraded cycling performance. The use of nonflammable, highly stable, and robust solid-state electrolytes (SSEs) is anticipated to fundamentally resolve these issues, positioning solid-state LOBs as a focal point of research in recent years. MOFs have emerged as promising candidate materials for SSEs owing to their ordered porous structure, abundant active sites, and tunable chemical composition, thereby enabling enhanced ionic conductivity and compatibility with electrodes. Wang et al.^112^ successfully prepared a MOF-based SSE by tailoring the unsaturated metal sites and molecular pore sizes of MOFs, constructing multiple low-impedance interfaces between SSEs and electrodes to fabricate highly safe and stable solid-state LOBs. The study developed a novel air-stable SSE material, UiO-67-Li, based on the UiO-67 MOF, featuring high ionic conductivity. Owing to its directional ion-transport channels and abundant ion-conduction sites, the UiO-67-Li SSE exhibits an ionic conductivity as high as 6.4 × 10^–4^ S cm^–1^, a Li^+^ transference number of 0.65, and excellent stability toward air components and the anode, effectively addressing the issues of poor stability and difficult interface construction in conventional SSEs (Figure 12a and b). Additionally, by *in situ* growing this electrolyte on a porous conductive graphene aerogel substrate, a solid-state air cathode (UiO-67-Li@rGO) with continuous electron, ion, and molecular transport pathways was obtained. Furthermore, the *in situ* growth strategy for integrating MOF-based SSEs with electrode materials enables the formation of wetted “electrolyte/electrode” low-impedance elastic contact interfaces, significantly enhancing Li^+^ transport. Based on these designs, the solid-state LOB demonstrates 115 cycles of reversible and stable operation. This work paves a new path for developing high-performance SSE materials and constructing safe and long-life solid-state lithium metal batteries. Subsequently, Miao et al.^147^ employed a directed modification strategy to fabricate cationic MOF (CMOF) and anionic MOF (AMOF) SSEs by incorporating functional groups into neutral UiO-66 (Figure 12c). Specifically, CMOF is constructed by introducing positively charged – N^+^(CH_3_)_3_ groups into the ligands, forming a positively charged framework, while AMOF features negatively charged – SO^3–^ groups to create a negatively charged structure. Both materials exhibit ordered pore structures and high crystallinity, providing directional pathways for Li^+^ transport. CMOF demonstrates excellent Li^+^ conductivity, effectively accelerating ion migration while suppressing anion movement to reduce battery polarization (Figure 12d and e). Additionally, it shows outstanding chemical stability, high tolerance toward water, O^2–^, and Li_2_O_2_, as well as flame-retardant properties, addressing the safety issues of flammability and decomposability in traditional liquid electrolytes. The solid-state LOB using CMOF as the electrolyte achieves a cycle life of up to 790 h with a low overpotential of 1.09 V, significantly outperforming batteries with conventional electrolytes. As an SSE, AMOF also enhances Li^+^ conductivity, verifying the universality of the directed modification strategy.

**Figure 12:**
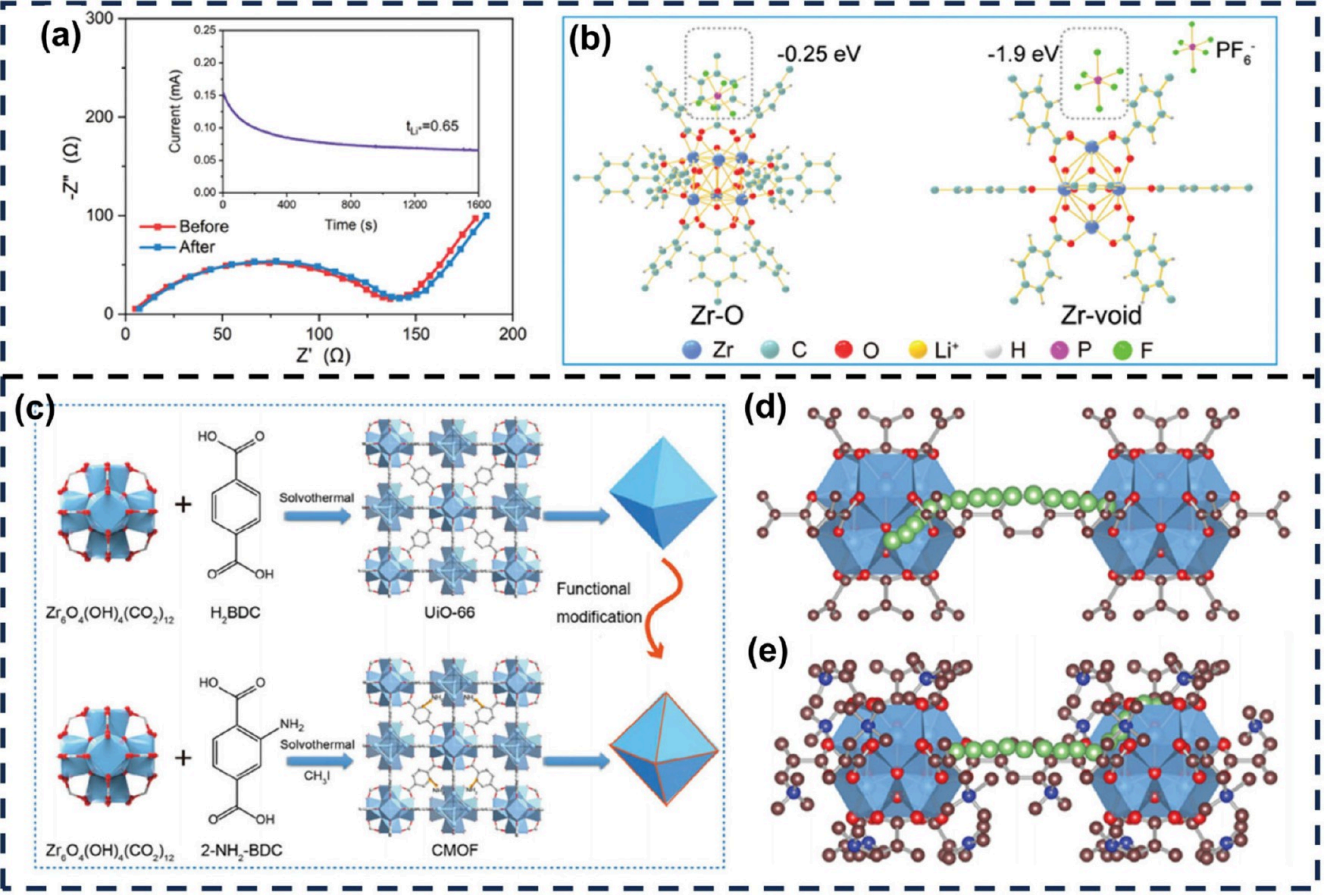
(a) Electrochemical impedance spectroscopy (EIS) before and after polarization of the Li|UiO-67-Li SSEs|Li cell, and the inset is the current–time curve at 10 mV of polarization. (b) DFT calculations of PF_6_^–^ adsorption configurations on different Zr sites. Reproduced with permission from ref 112. Copyright 2022 Wiley-VCH. (c) Schematic illustration of the synthesis of UiO-66 and CMOF. (d-e) Li^+^ transfer paths of UiO-66 and CMOF. Reproduced with permission from ref 147. Copyright 2024 Wiley-VCH.

Polymer-based SSEs have emerged as a transformative alternative to inorganic counterparts, offering superior mechanical compliance, scalable processability, and interfacial engineering capabilities that enable ultralow interfacial resistance. However, the ionic conductivity and Li^+^ transference number of polymer-based SSEs remain unsatisfactory, mainly owing to sluggish Li^+^ transport and undesired anion migration caused by the high crystallinity of polymer chains and insufficient dissociation of lithium salts. The porous channels and abundant active sites of MOFs enable favorable interactions with both polymer chains and lithium salt anions, thus effectively enhancing the performance of polymer-based SSEs. Xu et al.^148^ developed a 3D continuous interconnected MOF-based polymer electrolyte via an electrospinning strategy to construct a polyacrylonitrile (PAN) fiber network, followed by *in situ* growth of MOF808 crystals. The PAN fibers serve as a supporting skeleton, with uniformly distributed MOF808 crystals forming a 3D network on the fiber surface, which is further composited with poly­(ethylene oxide)–lithium bis (trifluoromethanesulfonyl) imide (PEO–LiTFSI). This hybrid material integrates the porous structure of MOF808 and the flexibility of the polymer matrix: the large-pore cage structure and unsaturated metal sites of MOF808 efficiently adsorb anions, promoting Li^+^ dissociation and directional transport, while the PAN fiber network enhances mechanical strength and structural stability. Infrared Raman spectroscopy and synchrotron small-angle X-ray scattering reveal that ion-cluster transport at the MOF/polymer interface increases local Li^+^ flux concentration, boosting Li^+^ transference number to 0.42 and ensuring uniform Li^+^ flux distribution for dendrite-free and homogeneous Li deposition. Nanoindentation tests demonstrate that the high modulus and elastic recovery of the MOF-based polymer solid electrolyte facilitate the formation of a robust, dendrite-resistant interface. As a result, the symmetric battery system exhibits a minimal overpotential of 35 mV and stable cycling exceeding 1800 h, enabling low-overpotential lithium deposition. Polyvinylidene fluoride (PVDF)-based polymer electrolytes have been widely utilized in LOBs, exhibiting remarkable performance. Nevertheless, side reactions induced by residual solvents in this system severely compromise the operational stability of LOBs. Compared with other filler materials, MOFs possess unique and inherent advantages in confining residual solvents owing to their distinctive structures. Wang et al.^115^ designed a novel composite polymer electrolyte (MOF@PVDF) by introducing amino-modified zirconium-carboxylate NH_2_–UIO-66 MOF with abundant defects as a filler. Using PVDF as the matrix, the open metal sites and functional groups of the MOF establish strong interactions with residual solvent molecules, reconstructing the Li^+^ solvation structure to reduce solvent decomposition and anchor anions. MOF@PVDF integrates the regular porosity and high specific surface area of MOF with the flexibility of PVDF, achieving an ionic conductivity of 3.6 × 10^–4^ S cm^–1^ (double that of pure PVDF) along with a reduced activation energy of 0.20 eV and significantly enhanced mechanical strength (fracture strength up to 2.9 MPa). In LOBs, MOF@PVDF suppresses lithium dendrite growth by homogenizing Li^+^ flux, enabling Li/Li symmetric batteries to cycle stably at 0.2 mA cm^–2^ for over 800 h. Meanwhile, the strong solvent-anchoring effect of the MOF reduces organic SEI components, forming a stable inorganic-rich interface that allows LOBs to achieve a long cycle life of 1100 h at 200 mA g^–1^ with an expanded electrochemical window up to 4.6 V. This strategy addresses the side reactions and interfacial instability caused by residual solvents in traditional PVDF electrolytes by tailoring the solvation environment, providing a new electrolyte design concept that integrates high ionic conductivity, uniform lithium deposition, and interfacial compatibility for high-energy-density and safe solid-state LOBs.

### 3.4 Summary

In summary, the pristine MOFs exhibit unique multifunctional characteristics in LOB systems, providing innovative material solutions for enhancing battery performance. As cathode materials, their ordered porous structures and tunable active sites can effectively promote the kinetics of ORR/OER, optimize the nucleation and decomposition processes of discharge products, and significantly improve battery cycling stability. As separator modification materials, the molecular sieve effect of MOFs enables selective Li^+^ transport while blocking the shuttling of harmful substances, and their uniform pore structures help regulate Li^+^ flux distribution to inhibit dendrite growth. In electrolyte systems, the regular nanopores of MOFs serve as ideal ion transport channels, while their surface chemical properties can precisely regulate electrolyte components to suppress side reactions. Beyond enhancing ORR/OER catalytic activity, MOF-based materials suppress side reactions in LOBs through the following aspects: ordered pore channels physically block unstable components from diffusing to the anode; metal sites and functional groups regulate the solvation structure of Li^+^, inhibiting electrolyte decomposition and the accumulation of Li_2_CO_3_/LiOH; electronic structure modulation optimizes intermediate adsorption, guiding reversible Li_2_O_2_ formation/decomposition, thereby significantly improving reversibility and cycling stability. Current research challenges mainly focus on environmental stability, interface compatibility, and large-scale fabrication of materials. Future developments should focus on the design of new stable MOFs, optimization of material–electrolyte interface engineering, and integration of multiscale simulations with *in situ* characterization techniques. Through synergistic innovation in materials chemistry and electrochemistry, MOFs are expected to drive LOBs toward the practical goals of high energy density and long cycle life, providing crucial support for the development of next-generation energy storage systems. Breakthroughs in this field will rely on an in-depth understanding of material structure–performance relationships, as well as continuous optimization of preparation processes and device integration technologies.

## 4 MOF Derivatives

Undoubtedly, pristine MOFs demonstrate compelling merits in advancing LOBs conversion. However, their practical implementation faces intrinsic limitations, including compromised mechanical robustness, insufficient electrical conductivity, and suboptimal structural stability. In contrast, MOF derivatives have garnered increasing attention in LOB applications by preserving the inherent porosity of parent frameworks while enabling precise modulation of active components.^149^ MOF derivatives can significantly enhance the electrical conductivity and structural stability of catalysts, while featuring a high density of active sites, making them highly suitable for constructing electrodes with high catalytic activity, long cycle life, and excellent stability. Through rational postsynthetic modifications, MOFs can be transformed into diverse functional materials encompassing metallic compounds, nanoporous carbons, and their hybrid architectures. The integrated carbon matrices facilitate efficient electron transport pathways, while strategically engineered hollow configurations effectively mitigate volume fluctuation during cycling. These synergistic advantages of abundant active sites, enhanced conductivity, and hierarchical nanostructures collectively position MOF derivatives as superior electrocatalysts for LOBs. This section systematically examines state-of-the-art MOF-derived materials, encompassing carbon-based architectures, metallic species, metal oxides, sulfides, phosphides, selenides, and other emerging variants (Table 2).

**Table 2:** Summary of MOF Derivatives for LOBs

MOFs	Sample	Discharge capacities (mAh g^–1^)	Cycle number	refs
Co-MOF	3DP-NC-Co	1124 (0.05 mA cm^–2^)	160 h (0.1 mA cm^–2^)	^150^
MIL-100(Fe)	F-PNG	7150 (0.1 mA cm^–2^)	30 (0.1 mA cm^–2^)	^151^
Fe-MOF	N-Fe-MOF	5300 (50 mA g^–1^)	50 (400 mA g^–1^)	^152^
[H_2_im]_4_[Mo_8_O_26_(Him)_2_]	α-MoC_*1–x*_	21212 (200 mA g^–1^)	100 (200 mA g^–1^)	^153^
ZIF-8	NPC/rGO	12000 (200 mA g^–1^)	125 (200 mA g^–1^)	^154^
ZIF-8	H-NC	18762 (500 mA g^–1^)	212 (500 mA g^–1^)	^155^
ZIF-67	CuO-CuCO_2_O_4_	6844 (100 mA g^–1^)	111 (400 mA g^–1^)	^156^
ZIF-67	Co@NC-900/AB	3110 (0.1 mA cm^–2^)	80 (0.05 mA cm^–2^)	^157^
Ru-MOF	Ru-MOF-C	–	800 (500 mA g^–1^)	^109^
Fe/Co-ZIF-8	Fe/Co–N–C	8670 (100 mA g^–1^)	180 (100 mA g^–1^)	^158^
Ru-MOF	Ru_0.3_SAs-NC	13424 (0.02 mA/cm^2^)	60 (0.02 mA/cm^2^)	^159^
Co/Zn-MOF	Co-SAs/N–C	20105 (100 mA g^–1^)	260 (400 mA g^–1^)	^160^
MIL-101(Cr)	Cr_2_O_3_@OPC	4800 (0.1 mA cm^–2^)	50 (0.1 mA cm^–2^)	^161^
MIL-100(Fe)	γ-Fe_2_O_3_/carbon	5970 (0.1 mA cm^–2^)	30 (0.1 mA cm^–2^)	^162^
MET-2	Mn_2_O_3_ nanocages	21070.6 (500 mA g^–1^)	180 (500 mA g^–1^)	^113^
ZIF-67	Co_3_O_4_ nanocage	15500 (0.1 A g^–1^)	132 (0.1 A g^–1^)	^163^
ZIF-8@ZIF-67	GPC-Co_3_O_4_	1575 (125 mA g^–1^)	50 (125 mA g^–1^)	^106^
Co-MOF	carbon/Co_3_O_4_	9850 (100 mA g^–1^)	320 (500 mA g^–1^)	^164^
Ni–Co MOF	NCO@GNS	7201 (100 mA g^–1^)	200 (500 mA g^–1^)	^165^
ZIF-67	CeO_2_/Co_3_O_4_	9443 (100 mA g^–1^)	140 (100 mA g^–1^)	^111^
ZIF-8@ZIF-67	Co_3_O_4_@NiCo_2_O_4_	11672.8 (600 mA g^–1^)	280 (600 mA g^–1^)	^166^
ZIF-9	Co-CoO@NC	5582 (0.05 mA cm^–2^)	42 (0.1 mA cm^–2^)	^167^
ZIF-67	Co_9_S_8_@CPNs	7000 (50 mA g^–1^)	110 (100 mA g^–1^)	^168^
ZIF-67	CoS_2_@NC	3529.8 (0.1 mA cm^–2^)	100 (0.1 mA cm^–2^)	^51^
ZIF-67	C/MoS_2_–CoMo_2_S_4_	17551 (500 mA g^–1^)	330 (500 mA g^–1^)	^169^
Co-MOF	ZnCo_2_S_4_	9505 (100 mA g^–1^)	90 (100 mA g^–1^)	^170^
Ni-MOF	Ni_3_S_2_	7478 (100 mA g^–1^)	120 (300 mA g^–1^)	^171^
ZIF-67	CoP@PNCF-700	9630.5 (100 mA g^–1^)	187 (100 mA g^–1^)	^172^
ZIF-67	Fe-CoP@DPA	18208.5 (100 mA g^–1^)	282 (200 mA g^–1^)	^173^
ZIF-67	NiCoP@PNCF	13070.2 (200 mA g^–1^)	242 (200 mA g^–1^)	^174^

### 4.1 Cathode

While conventional MOFs exhibit intrinsically low electronic conductivity, necessitating conductive additives for practical deployment in electrocatalysis and energy storage systems, thermally derived MOF architectures retain the morphological fidelity and hierarchical porosity of their precursors, synergistically enhancing Li^+^ and O_2_ diffusion kinetics in LOBs. The highly conductive carbon matrices derived from pyrolysis establish efficient electron transport networks, while uniformly dispersed metallic catalytic sites substantially accelerate ORR/OER kinetics through optimized active site exposure and reduced activation barriers. This integrated structural design bridges the gap between ionic/electronic transport efficiency and catalytic activity, positioning MOF derivatives as multifunctional platforms for advanced energy conversion technologies. For MOF materials, the pyrolysis temperature directly determines their electrical conductivity, defect concentration, and active site structure, with a moderate temperature achieving an optimal balance among these three factors. An oxidizing atmosphere favors the formation of metal oxides, an inert atmosphere readily yields carbon-coated structures, and a reducing atmosphere produces metal/carbon composites. Furthermore, the metal ratio and ligand structure of the precursor jointly determine the heterojunction strength, pore architecture, and the number of active sites.

3. Develop multiscale structural engineering and topology-guided assembly to construct gradient-pore 3D conductive networks for high-performance batteries.

#### 4.1.1 MOF-Derived Carbon-Based Materials

Thermal treatment of MOFs under inert atmospheres (e.g., Ar, N_2_) at elevated temperatures induces cleavage of coordination bonds and carbonization of organic linkers, typically yielding metal-doped carbon matrices. Given the inherent low conductivity of pristine MOFs (a critical limitation for LOBs cathode applications), their controlled conversion into carbonaceous derivatives emerges as a strategic approach to substantially enhance electronic conductivity. This transformation simultaneously preserves, albeit partially, the hierarchical porosity and catalytically active components inherited from the precursor frameworks. Such dual optimization of conductive networks and active site accessibility establishes MOF-derived carbons as multifunctional electrocatalytic platforms, addressing both charge transfer limitations and reaction kinetics challenges in advanced battery systems.^175^ Choi et al.^176^ reported a strategy for the high-quality synthesis and stabilization of subnanometric particles (SNPs) within multishell hollow MOFs (Figure 13a). The approach involves first constructing multilayer MOFs by alternating the stacking of water-labile and water-stable MOF layers. Leveraging controlled hydrogen-bonding affinity, isolated water molecules are selectively sieved through the hydrophobic nanocages of the water-stable MOF and sequentially transferred into the water-labile MOF layers. Transport of water molecules through the water-stable MOF layers via this controlled hydrogen-bonding interaction represents a critical step for realizing SNPs within alternating water-labile and water-stable layer structures. This process converts the multilayer MOFs into SNP-embedded multishell hollow MOFs. Additionally, the multishell architecture stabilizes SNPs through π backbonding, enabling high electrical conductivity via a hopping mechanism, while the hollow interstitial spaces minimize transport resistance. As demonstrated by SNPs embedded in multishell hollow MOFs with up to five shells, these features endow LOBs with superior electrochemical performance, including high volumetric capacity and low overpotential. By contrast, nitrogen-doped carbon (NC) matrices possess intrinsically higher electronic conductivity. Organic nitrogen-containing ligands such as 2-methylimidazole and ferric hexacyanoferrate are commonly used to fabricate NC structures. As previously discussed, MOFs constructed from these organic ligands have undergone systematic pyrolysis, with their derived NC composites investigated as cathode candidates for LOBs. Lyu et al.^150^ demonstrated a pioneering integration of 3D printing with MOF-derived engineering to fabricate freestanding hierarchically porous carbon frameworks (3DP-NC-Co) for advanced LOBs applications. Utilizing cobalt-based MOF precursors blended with Pluronic F127 in a shear-thinning rheological ink, the extrusion-based additive manufacturing process precisely constructs 3D networked architectures. Subsequent pyrolysis under a nitrogen atmosphere transforms the printed scaffolds into NC matrices embedded with uniformly dispersed cobalt nanoparticles. The resulting architecture exhibits a multiscale pore system, featuring macropores (0.8–8 μm) from the 3D-printed framework and intrinsic meso/micropores within carbon lamellae, synergistically addressing Li_2_O_2_ accommodation and ionic/molecular transport challenges (Figure 13b). The framework itself offers high electronic conductivity (2.2 × 10^3^ S m^–1^) and mechanical stability, eliminating the need for additional conductive matrices and reducing the proportion of inactive materials, thus achieving a practical specific energy of 798 Wh kg^–1^ (based on total battery mass). The embedded Co nanoparticles and NC synergistically enhance ORR and OER reactions, reducing charge and discharge overpotentials and promoting reversible decomposition of Li_2_O_2_. At 0.05 mA cm^–2^, the discharge capacity reaches 1124 mAh g^–1^ (based on electrode mass), with cycle stability surpassing that of traditional carbon paper-supported electrodes. By precisely tailoring pore structures and active site distributions, this 3D-printed MOF-derived porous framework addresses the challenges of Li_2_O_2_ deposition/decomposition in LOBs, simultaneously eliminating the weight penalty of conventional conductive matrices to significantly enhance practical energy density and cycling efficiency. This strategy provides a novel design concept for high-energy-density LOBs and demonstrates the potential of 3D printing in fabricating complex electrode architectures.

**Figure 13:**
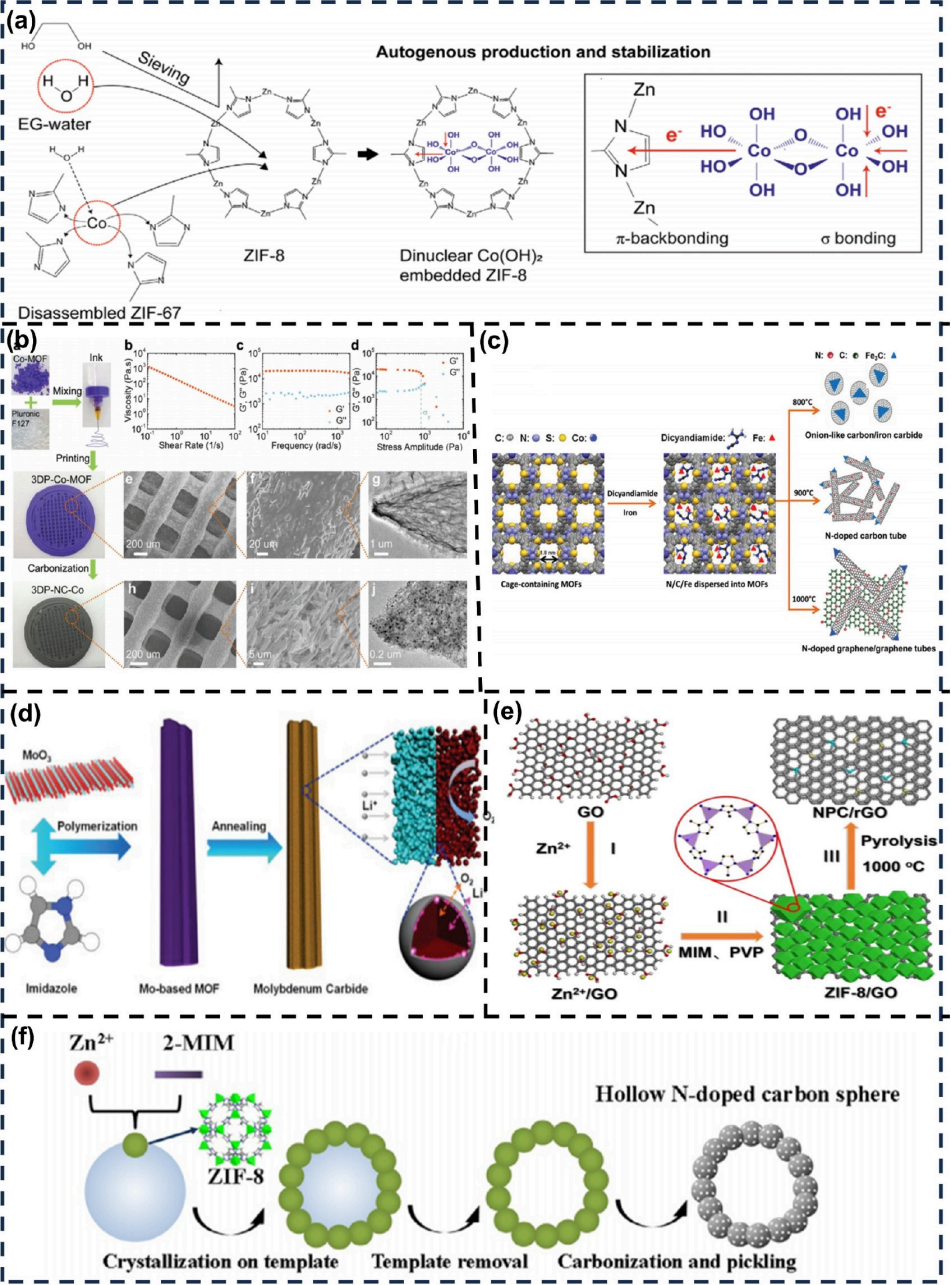
(a) Illustration of the autogenous production and stabilization of Co­(OH)_2_ SNPs in a micropore. Reproduced with permission from ref 176. Available under a CC-BY 4.0 license. Copyright 2020 Wiley-VCH Choi et al. (b) Preparation and characterization of 3D-printed Co-MOF-derived framework. Reproduced with permission from ref 150. Copyright 2019 Wiley-VCH. (c) Schematic illustration of the formation of the carbon nanostructures found in the N–Fe-MOF catalysts synthesized at different heating temperatures. Reproduced with permission from ref 152. Copyright 2014 Wiley-VCH. (d) Schematic illustration of the synthesis process of nitrogen-doped porous molybdenum carbide nanoarchitectures. Reproduced with permission from ref 153. Copyright 2018 Royal Society of Chemistry. (e) Schematic illustration for the preparation process of NPC/rGO composite. Reproduced with permission from ref 154. Copyright 2017 Royal Society of Chemistry. (f) Schematic illustration of the synthesis of H-NC. Reproduced with permission from ref 155. Copyright 2021 Wiley-VCH.

In addition, MOF-derived metal carbides and their carbon-based composites display enhanced catalytic performance in LOBs. Li et al.^152^ developed a nitrogen-doped graphene/graphene tube nanocomposite (N–Fe-MOF) using cage-type MOF templates combined with a high-temperature pyrolysis strategy. Employing a cobalt-based cage MOF as the template, Dicyandiamide and an iron precursor were mixed and filled into the MOF pores, followed by pyrolysis at 800–1100 °C under nitrogen and subsequent acid washing to remove unstable components, yielding a composite with Fe_3_C cores and a nitrogen-doped graphene/graphene tube matrix (Figure 13c). The resulting catalyst consists of Fe/Fe_3_C nanoparticles embedded in nitrogen-doped graphene. The optimal N–Fe-MOF, pyrolyzed at 1000 °C, demonstrates superior ORR activity across different pH conditions, with improved cathode performance in LOBs attributed to the catalytic activity of Fe_3_C. Compared to derivatives prepared at different temperatures and control samples (including carbon black, Pt/C, and MOF-free N–Fe), the optimized N–Fe-MOF exhibits an onset potential of approximately 3.1 V in a practical nonaqueous electrolyte (0.1 mol L^–1^ LiPF_6_ in tetraethylene glycol dimethyl ether), closely approaching the theoretical value. The MOF template method precisely tailors the material’s hierarchical pore structure and nitrogen-doping configuration, combining Fe_3_C-catalyzed graphene tube growth to achieve high active-site density and rapid mass transfer, thereby significantly enhancing the energy density and cycling performance of LOBs. Furthermore, nitrogen-doped carbides typically exhibit high activity toward the ORR. Yu et al.^153^ pioneered a molybdenum carbide-based cathode architecture for LOBs through a MOF-derived carbonization strategy. By pyrolyzing molybdenum-based MOF precursors under controlled conditions, the team synthesized nitrogen-doped porous molybdenum carbides (denoted as α-MoC_1‑*X*_ and β-Mo_2_C) featuring self-assembled micron-scale rods composed of 15–20 nm nanocrystallites (Figure 13d). These hierarchical structures incorporate axially aligned mesoporous channels and macroporous tunnels, creating a bimodal pore architecture that simultaneously enhances electrochemically active site density and Li_2_O_2_ nucleation efficiency. The nitrogen doping further reduces nucleation overpotential while optimizing interfacial charge transfer. Electrochemical evaluations reveal the cubic α-MoC_1‑*X*_ phase outperforms hexagonal β-Mo_2_C, delivering an exceptional discharge capacity of 20212 mAh g^–1^ at 200 mA g^–1^ with a plateau voltage of 2.62 V versus Li^+^/Li. Remarkably, it maintains a capacity of 1,000 mAh g^–1^ over 100 cycles with 70% round-trip efficiency, coupled with a reduced charge voltage of 4.24 V, significantly lower than that of β-Mo_2_C counterparts. DFT calculations attribute this superiority to the cubic crystalline phase of α-MoC_1‑*X*_, which exhibits elevated surface Mo d-orbital electron density and oxygen adsorption energy due to its closer proximity of band centers to the Fermi level. This electronic configuration facilitates optimized oxygen adsorption/desorption kinetics and reduced activation barriers. This methodology demonstrates how phase-engineered molybdenum carbides, synergistically integrated with nitrogen doping and multimodal porosity, address critical challenges in LOBs. The work establishes a blueprint for rational design of transition metal carbide cathodes through crystal phase modulation and defect engineering.

A facile MOF-mediated synthesis strategy has been reported for constructing metal-free carbonaceous porous architectures. Zhong et al.^154^ engineered nitrogen-enriched porous carbon/reduced graphene oxide (NPC/rGO) hybrids through controlled pyrolysis of ZIF-8/GO composite precursors (Figure 13e). The resulting architecture features ZIF-8-derived N-doped carbon nanoparticles uniformly anchored on rGO nanosheets, forming an interconnected 3D conductive network with exceptional surface area (712.65 m^2^ g^–1^) and hierarchically porous architecture comprising mesopores (∼3.8 nm) and macropores. Remarkable nitrogen doping (6.23 wt.%) predominantly as pyridinic and pyrrolic configurations is achieved, creating abundant catalytically active sites. As cathode catalysts for LOBs, NPC/rGO demonstrates multifunctional advantages: The hierarchical porosity facilitates efficient O_2_ diffusion and Li_2_O_2_ accommodation, while the rGO matrix enhances electronic conductivity and prevents carbon nanoparticle aggregation. Synergistic interplay between conductive graphene networks and nitrogen dopants substantially improves ORR/OER kinetics, as evidenced by EIS revealing reduced charge transfer resistance and cyclic voltammetry showing enhanced current responses. The unique 3D porous framework enables optimized electrolyte infiltration, accelerated Li^+^ migration, and efficient gas-phase reactant accessibility, collectively addressing critical challenges in LOB systems. This MOF-derived design paradigm highlights the potential of defect-engineered carbon architectures for developing high-performance metal-free electrocatalysts through synergistic integration of hierarchical porosity, heteroatom doping, and conductive network optimization. Aiming to address the capacity and cycling performance limitations of conventional carbon materials in LOBs due to structural constraints, He et al.^155^ developed a MOF-derived hollow NC sphere material (denoted as H-NC). Using polystyrene microspheres as templates, the team synthesized ZIF-8 core–shell structures, which were subsequently pyrolyzed at high temperature and treated with acid washing to remove metal components, yielding hollow NC spheres (Figure 13f). H-NC features a high specific surface area (884 m^2^ g^–1^) and hierarchical pore structure, where the hollow interior provides unobstructed channels for O_2_ and Li^+^ diffusion and ample space for Li_2_O_2_ deposition, while nitrogen doping enhances the catalytic activity toward ORR and OER reactions. Experimental results show that batteries employing H-NC cathodes achieve an initial discharge capacity of 18762 mAh g^–1^ at 500 mA g^–1^ and maintain stable capacity over 212 cycles, significantly outperforming conventional NC materials. This superior performance stems from the hollow structure, mitigating product accumulation and the NC promoting reaction kinetics, thus offering a novel design paradigm for high-performance LOB cathodes.

#### 4.1.2 MOF-Derived Metals

Uniform dispersion of metallic active sites and their strong interactions with the support significantly enhance the catalytic activity toward ORR and OER, reducing overpotentials and accelerating reaction kinetics. MOF-derived metal-based materials have demonstrated broad application potential in LOBs. The Co and Ni-based active sites exhibit superior catalytic activity toward the OER, effectively reducing the charging overpotential. The Fe and Mn-based sites show advantages in the ORR, promoting efficient oxygen reduction and Li_2_O_2_ formation during discharge. Moreover, bimetallic or trimetallic systems can leverage electronic synergy to simultaneously optimize both ORR and OER activities, achieving bifunctional catalysis. Yu et al.^157^ prepared a bifunctional electrocatalyst featuring cobalt nanoparticles embedded in an NC polyhedral network (Co@NC) via pyrolysis of ZIF-67, where cobalt nanoparticles are uniformly distributed within a nitrogen-doped graphitized carbon matrix. Pyrolysis at 900 °C optimizes the material’s specific surface area (259.7 m^2^ g^–1^), graphitization degree, and nitrogen-doping configuration, synergistically enhancing electrical conductivity and catalytic activity. When applied in LOBs, the Co@NC-900/acetylene black electrode exhibits a high discharge capacity of 3110 mAh g^–1^ and excellent cycling stability over 80 cycles. This superior performance stems from the porous structure facilitating oxygen and electrolyte diffusion, graphitized carbon accelerating electron transport, and cobalt–nitrogen sites (Co–N_*x*_) collaboratively boosting ORR and OER kinetics. Compared with noble metal catalysts, this material overcomes the bottlenecks of high cost and insufficient activity in traditional catalysts through its low cost and abundant reserves, while structural design effectively mitigates volume expansion and byproduct accumulation, thereby providing a novel design concept for high-performance non-noble-metal-based LOBs. Beyond transition metal-based MOFs, noble metal-derived architectures have demonstrated remarkable potential in LOB systems. Meng et al.^109^ pioneered a ruthenium-embedded carbon composite catalyst (denoted as Ru-MOF–C-C) through controlled pyrolysis of Ru-MOF precursors. The synthesis involves solvothermal self-assembly of RuCl_3_ and H_3_BTC in acetic acid and water mixed solution to form crystalline Ru-MOF, followed by argon-assisted pyrolysis at 700 °C. This process yields spatially distributed ruthenium nanoparticles (∼5 nm) uniformly embedded within a conductive carbon matrix, creating a 3D composite architecture with surface-to-bulk active site continuity. Remarkably, this design enables a self-regenerative surface mechanism during cycling: While surface Ru nanoparticles may detach due to carbon oxidation, subsurface metallic species progressively migrate to replenish active sites, ensuring sustained catalytic activity. Electrochemical evaluations reveal exceptional performance, with Ru-MOF-C-based LOBs achieving ultralow discharge and charge overpotentials (0.2 and 0.7 V) and maintaining stable operation for 800 cycles at 500 mA g^–1^ with a limited capacity of 800 mAh g^–1^. The system demonstrates highly reversible Li_2_O_2_ formation and decomposition with minimal parasitic reactions, attributed to the synergistic combination of intrinsic catalytic activity of Ru and the protective confinement effect of carbon matrix. This work establishes a paradigm for designing durable noble metal catalysts through spatially engineered metal–carbon architectures that balance activity retention with structural stability.

In addition, bimetallic MOFs have been widely studied as precursors due to the synergistic effect of different metal sources.^177,178^ Shin et al.^179^ developed a hierarchically porous Co@C(700–1000) composite via pyrolysis of a Co/Zn bimetallic MOF precursor (Figure 14a). Using cobalt clusters as the catalyst precursor and zinc clusters as the sacrificial template, the team employed a two-step pyrolysis process: first generating ZnO at 700 °C under nitrogen, followed by evaporating ZnO at 1000 °C to form mesopores (24 nm) and macropores (>200 nm), yielding a composite with ∼ 20 nm cobalt nanoparticles uniformly dispersed in a porous carbon matrix. The material features a high specific surface area (2046 m^2^ g^–1^) and hierarchical pore structure, significantly facilitating Li^+^ and O_2_ diffusion and providing abundant active sites, while the catalytic activity of cobalt nanoparticles accelerates oxygen reduction/evolution reactions and lowers overpotentials. Experimental results demonstrate that LOBs based on Co@C (700–1000) exhibit a high specific capacity of 4 mAh cm^–2^ and stable cycling performance over 67 cycles with a limited capacity of 0.5 mAh cm^–2^, outperforming conventional carbon-based materials. Gharibi et al.^158^ demonstrated a time-dependent synthesis strategy to engineer bimetallic Fe/Co–N–C catalysts (denoted as Fe/Co–N–C (At once) and Fe/Co–N–C (10 h)) with tailored electrochemical properties. The methodology employs controlled injection protocols for introducing iron/cobalt salts and 2-methylimidazole into methanol, creating Fe/Co-ZIF-8 precursors with distinct coordination kinetics. Subsequent pyrolysis at 1000 °C under argon yields nitrogen-doped graphitic carbon matrices embedded with uniformly dispersed Fe/Co nanoparticles. Structural analyses reveal that Fe/Co–N–C (At once) possesses enhanced porosity (Brunauer–Emmett–Teller surface area >800 m^2^ g^–1^), enabling superior discharge capacity in LOBs through optimized O_2_ and Li^+^ diffusion. Conversely, Fe/Co–N–C (10 h) exhibits advanced graphitization carbon matrices and increased surface-exposed metallic sites, leading to a 32% improvement in electrochemically active surface area and exceptional stability with a bifunctional oxygen redox activity gap Δ*E* = 0.86 V in alkaline media. The synergistic interplay between hierarchical porosity and metal–carbon electronic interactions address critical limitations of conventional cathodes (rapid capacity fade and high overpotentials) by simultaneously enhancing Li_2_O_2_ deposition kinetics and charge transfer efficiency.

**Figure 14:**
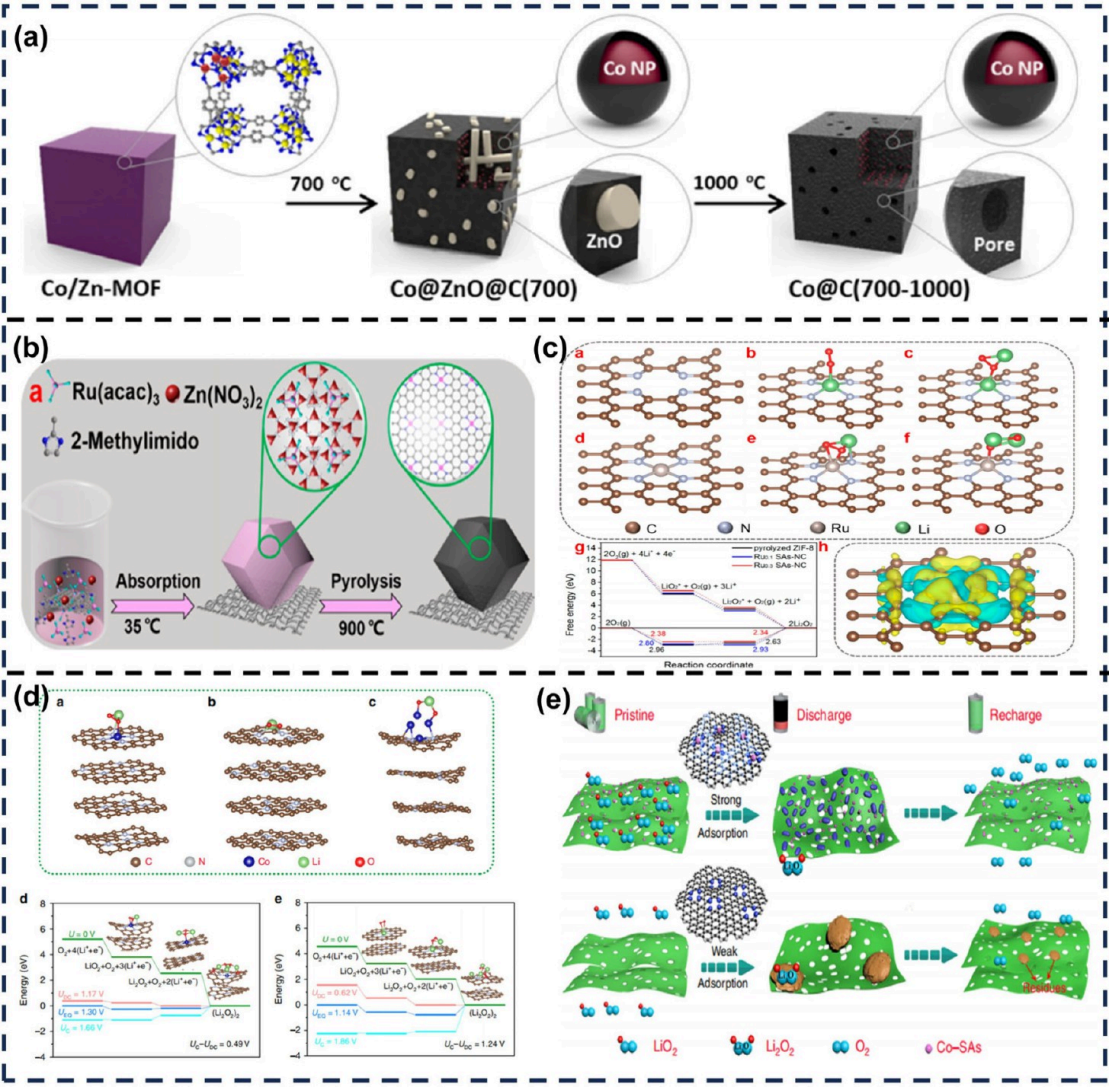
(a) Synthesis of Co@C(700–1000). Reproduced with permission from ref 179. Copyright 2022 Wiley-VCH. (b) Scheme of the formation of Ru SAs-NC, and (c) the DFT calculations of the catalysts. Reproduced with permission from ref 159. Copyright 2020 American Chemical Society. (d, e) DFT calculations and proposed mechanisms for Co-SAs/NC. Reproduced with permission from ref 160. Available under a CC-BY 4.0 license. Copyright 2020 Nature Publishing Group Wang et al.

4. Industrializing MOF-based catalysts requires long-term stability, low cost, and scalable fabrication, prioritizing robust design and economical processes.

SACs represent the ultimate frontier in metal particle size reduction, achieving maximal atomic utilization efficiency through monatomic configuration. These catalysts exhibit unparalleled catalytic activity and selectivity owing to their distinctive electronic configurations and quantum-level energy states, while serving as model systems for mechanistic studies due to their well-defined single active centers. MOFs emerge as exceptional host matrices for stabilizing SACs, leveraging their exceptional specific surface area, robust metal–ligand coordination bonds, tailorable porosity, and structural modularity. The carbonaceous matrices derived from MOFs via ligand pyrolysis endow SACs with superior electrical conductivity, positioning them as transformative electrocatalysts for next-generation LOB systems. This MOF-mediated SAC engineering paradigm bridges atomic-scale precision with macroscopic functionality, offering new opportunities to overcome fundamental limitations in oxygen electrocatalysis while maintaining structural durability under harsh battery operating conditions. Hu et al.^159^ developed ruthenium single-atom anchored nitrogen-doped porous carbon electrocatalysts (Ru SAs-NC) with tunable Ru loadings via spatial confinement and ion-exchange strategies using MOFs as precursors, achieving significant performance improvements in LOBs as cathode materials (Figure 14b). Using ZIF-8 as a template, the team adsorbed ruthenium precursors and performed high-temperature carbonization at 900 °C to replace zinc nodes with ruthenium ions, yielding a structure where Ru single atoms are uniformly dispersed in a NC skeleton with Ru–N_4_ coordination configurations as active centers. These Ru–N_4_ sites optimize the adsorption energies of LiO_2_ and Li_2_O_2_ (Figure 14c), boosting ORR and OER reaction kinetics to achieve ultralow charge–discharge overpotentials (0.55 V) and a high discharge capacity of 13424 mAh g^–1^. *In-situ* DEMS confirms an electron transfer efficiency close to the theoretical value (e^–^/O_2_ = 2.14). Additionally, the Ru SAs-NC electrode maintains stable operation for 60 cycles under a limited capacity of 1000 mAh g^–1^ with excellent impedance recovery, attributed to the uniform growth and complete decomposition of Li_2_O_2_. This work demonstrates that atomic-level active site design and interfacial electronic structure optimization can significantly enhance the energy efficiency and cycling durability of LOBs, providing new insights for applying SACs in metal–air batteries. Wang et al.^160^ devised an innovative green gas migration-trapping strategy to synthesize isolated cobalt single-atom sites embedded within NC matrices derived from ultrathin zinc-hexamine complexes (denoted as Co-SAs/NC), serving as bifunctional oxygen electrocatalysts. Benefiting from the synergistic combination of 2D MOF-derived architectures and uniformly dispersed atomic metal sites, the catalyst facilitates nanoconfined Li_2_O_2_ formation and decomposition during ORR and OER processes. This structural precision enables full exposure of active sites, yielding substantially accelerated redox kinetics with minimized overpotential. DFT simulations further reveal that Co–N_4_ moieties function as catalytic epicenters, significantly strengthening LiO_2_ adsorption energy and fundamentally regulating the size distribution and morphological evolution of Li_2_O_2_ products (Figure 14d and e). The Co-SAs/NC-based cathodes demonstrate remarkably reduced charge and discharge polarization coupled with exceptional cycling stability. Notably, the preferential formation of LiOH discharge products (exhibiting enhanced chemical and electrochemical stability compared to conventional Li_2_O_2_) further contributes to system durability. This work establishes a molecular-level design paradigm for SACs through MOF-mediated spatial confinement strategies, bridging atomic-scale active site engineering with macroscopic battery performance optimization. The large-scale implementation of LOBs with LiOH chemistry faces formidable challenges due to severe internal shuttling of water additives that disrupt the desired four-electron electrochemical pathway. Addressing this critical issue, Zhang et al.^180^ developed an innovative MOF-derived water-trapping catalyst featuring atomically dispersed Co–N_4_ centers anchored on 3D interconnected graphene networks (Co-SA-rGO). This architecturally engineered catalyst demonstrates dual functionality: the Co–N_4_ sites exhibit enhanced activity toward proton-coupled electron transfer, enabling direct 4e^–^ involved LiOH formation, while the hierarchical mesoporous architecture with substantial surface area effectively confines approximately 12 wt % H_2_O molecules through chemisorption. The 3D conductive network further ensures efficient oxygen diffusion pathways and Li^+^ transportation channels. Remarkably, the Co-SA-rGO-based LOBs achieve an elevated discharge plateau of 2.83 V with an exceptional capacity of 12760.8 mAh g^–1^. Moreover, the engineered cathode demonstrates remarkable environmental stability, maintaining consistent discharge plateaus over 220 cycles even under ambient atmospheric conditions, representing a significant advancement in practical metal–air battery development.

#### 4.1.3 MOF-Derived Metal Oxide

Beyond the aforementioned strategies, MOFs demonstrate exceptional versatility as sacrificial templates for synthesizing metal oxide architectures through thermal treatment under oxidative gas atmospheres such as air or O_2_. The superiority of these MOF-derived metal oxides over elemental metals stems from their inherent catalytic functionalities and enhanced stability under operational conditions. Notably, the distinct advantage of MOF precursors lies in their capacity to engineer derivatives with precisely tailorable architectures and tunable surface chemistries through controlled pyrolysis processes. This structural precision enables systematic optimization of active sites and mass transport pathways, which proves critical for advancing electrocatalytic systems in sustainable energy applications. Gan et al.^161^ strategically designed an octahedral hierarchical chromic oxide-octahedral porous carbon (Cr_2_O_3_@OPC) composite (Figure 15a) through controlled pyrolysis of MIL-101 (Cr) precursors, retaining the octahedral morphology of its parent framework. The hierarchical architecture comprises uniformly dispersed Cr_2_O_3_ nanoparticles embedded within a mesoporous carbon matrix, where the partially graphitized carbon skeleton ensures superior electronic conductivity. Synergistic effects arise from redox-active Cr^3+^/Cr^6+^ species on nanoparticle surfaces that catalyze both Li_2_O_2_ formation and decomposition kinetics, coupled with the mesoporous network’s dual functionality in accommodating discharge products and facilitating efficient mass transport of oxygen and electrolyte. This engineered configuration delivers exceptional electrochemical performance, achieving a high discharge capacity of 4800 mAh g^–1^ at 0.1 mA cm^–2^ with a stabilized charge plateau at 3.9 V. Moreover, the composite demonstrates remarkable cyclability with 50 stable cycles at a limited capacity of 800 mAh g^–1^, outperforming the 11-cycle lifespan of carbon-based electrodes. This enhanced durability originates from the porous carbon matrix’s dual role in spatially confining Cr_2_O_3_ nanoparticles while structurally mitigating active site degradation and parasitic side reactions. Building upon the structural merits of iron-based MIL-100 MOFs, Chen et al.^162^ engineered a hierarchically mesoporous γ-Fe_2_O_3_/carbon nanocomposite through precision-controlled pyrolysis. This architecturally optimized material features uniformly dispersed γ-Fe_2_O_3_ nanoparticles embedded within a partially graphitized porous carbon matrix, where the hierarchical mesoporous architecture simultaneously provides abundant catalytically active sites and facilitates efficient mass transport of gaseous and electrolyte species. The synergistic integration of conductive carbon frameworks with redox-active iron oxide domains significantly enhances both oxygen reduction and evolution reaction kinetics. Electrochemical evaluations confirm exceptional performance metrics, with the composite cathode achieving an impressive discharge capacity of 5970 mAh g^–1^ at 0.1 mA cm^–2^ alongside well-defined discharge and charge plateaus at 2.7 and 3.75 V, respectively. Notably, the system demonstrates substantially improved cyclability, maintaining over 30 stable cycles under capacity-limited conditions at 600 mAh g^–1^. The performance leap is attributed to the carbon matrix’s dual role in ensuring electronic percolation while mitigating nanoparticle aggregation during repeated cycling.

**Figure 15:**
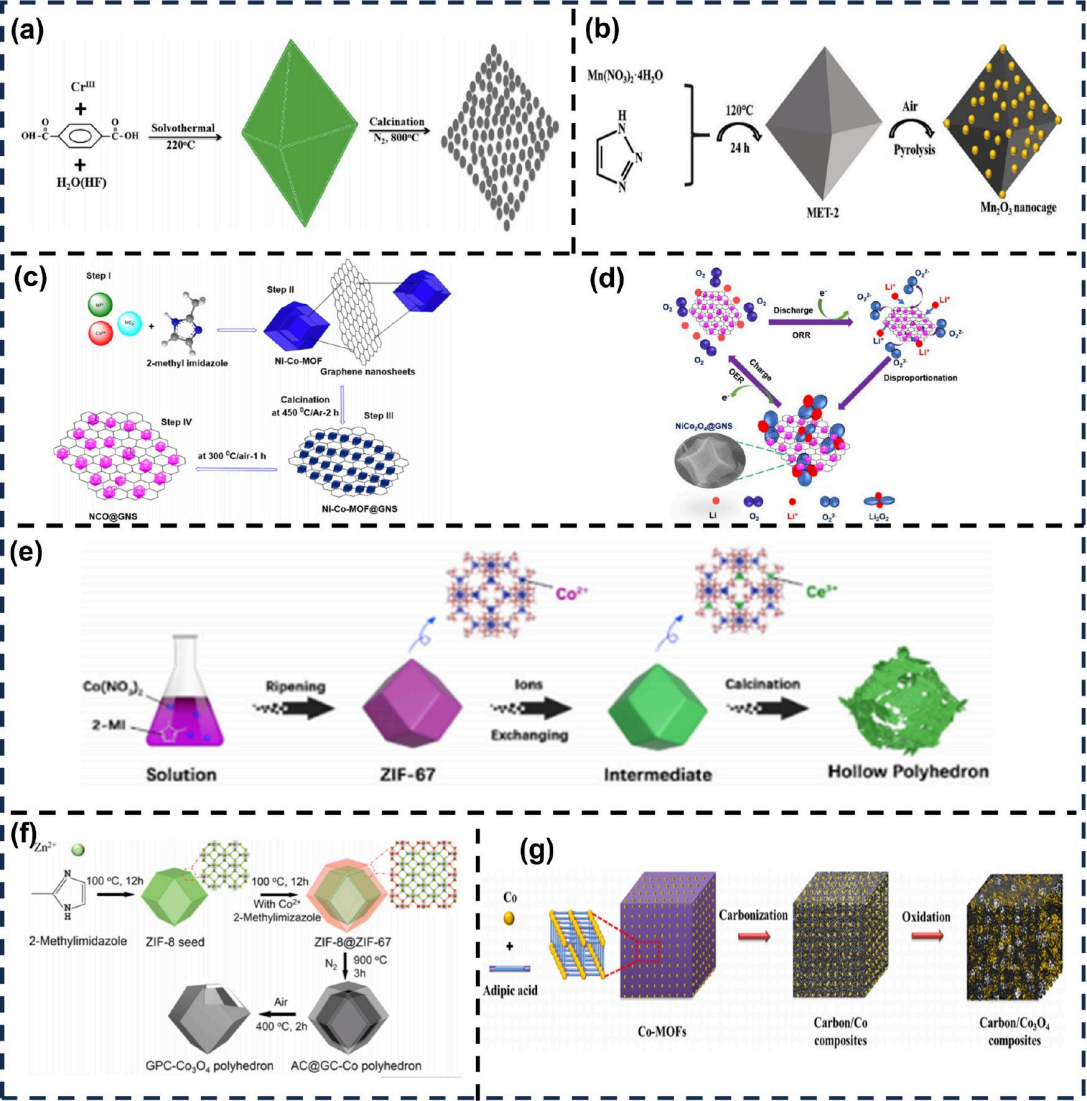
(a) The synthetic route of Cr_2_O_3_@OPC octahedral composites. Reproduced with permission from ref 161. Copyright 2016 Elsevier. (b) Schematic of the synthesis of Mn_2_O_3_ nanocages. Reproduced with permission from ref 113. Copyright 2023 Wiley-VCH. (c–d) Schematic representation of the synthesis of NCO@GNS composite bifunctional catalysts. Reproduced with permission from ref 165. Copyright 2021 Elsevier. (e) Schematic illustration for the fabrication of hollow CeO_2_/Co_3_O_4_ polyhedrons. Reproduced with permission from ref 111. Copyright 2021 Elsevier. (f) Synthetic Scheme for the preparation of core–shell ZIF-8@ZIF-67 crystals, AC@GC-Co, and GPC-Co_3_O_4._ Reproduced with permission from ref 106. Copyright 2016 American Chemical Society. (g) Schematic illustration of the formation procedure of the carbon/Co_3_O_4_ composites via post two thermal treatments. Reproduced with permission from ref 164. Copyright 2017 Elsevier.

Rational design of strategies for the precise introduction of defects into catalyst lattices, the controlled tailoring of defect types and concentrations, and their homogeneous distribution within the active phase, has emerged as a highly effective approach to modulating electronic structures, optimizing the adsorption behavior of reaction intermediates, and enhancing redox reaction kinetics. Pioneering this approach, Zhao et al.^113^ developed oxygen-vacancy-rich Mn_2_O_3_ nanocages via precisely controlled pyrolysis of manganese-based 1,2,3-triazolate frameworks (MET-2) at 350 °C (Figure 15b). This architectural engineering preserves the octahedral morphology of the parent MOF while creating a 3D mesoporous hollow structure composed of interconnected Mn_2_O_3_ nanoparticles. The hierarchical porosity not only accommodates substantial Li_2_O_2_ deposition but also establishes continuous channels for rapid O_2_ diffusion and Li^+^ migration. Synergistic modulation between oxygen vacancies and Mn^2+^/Mn^3+^ redox couples substantially reduces activation barriers for ORR and OER processes while enhancing intermediate adsorption and desorption kinetics and suppressing parasitic reactions. Electrochemical characterization reveals extraordinary performance metrics: an ultrahigh discharge capacity of 21070.6 mAh g^–1^ at 500 mA g^–1^ with a minimal overpotential of 0.81 V, coupled with remarkable cyclability of 180 stable cycles under capacity-limited conditions at 600 mAh g^–1^. This MOF pyrolysis strategy ingeniously balances oxygen vacancy concentration with framework stability, overcoming the intrinsic limitations of conventional transition metal oxides in conductivity and active site availability. Subsequently, Jiang et al.^163^ fabricated hierarchical Co_3_O_4_ nanocages via pyrolysis of ZIF-67. The unique 3D mesoporous architecture of the material provides efficient pathways for O_2_ and Li^+^ diffusion and ample space for Li_2_O_2_ deposition, while the synergistic effect of oxygen vacancies and Co^2+^/Co^3+^ redox couples enhances catalytic activity toward both ORR and OER reactions. Experimental results demonstrate that this material achieves an ultrahigh discharge capacity of 15500 mAh g^–1^ at a current density of 0.1 A g^–1^, and maintains stable cycling for over 132 cycles under a limited capacity of 1000 mAh g^–1^ at 0.5 A g^–1^. Its superior performance is attributed to the abundant active sites provided by the high specific surface area, the mass transport pathways optimized by the mesoporous structure, and the reversible reaction kinetics promoted by oxygen vacancies. Additionally, EIS that Co_3_O_4_ nanocages exhibit the best impedance recovery during cycling, indicating effective decomposition of Li_2_O_2_ and suppressed accumulation of side products.

Beyond monometallic MOFs, bimetallic MOFs have emerged as versatile precursors for bimetallic oxides (e.g., spinel oxides), which serve as pivotal electrode materials for lithium-based and metal–air battery technologies. Advancing this frontier, Palani et al.^165^ engineered a bifunctional cathode (NCO@GNS, Figure 15c) through ultrasonication-assisted assembly of ZIF-67-derived 3D porous NiCo_2_O_4_ dodecahedral nanosheets (NCO) with 2D graphene nanoplates (GNS). The resultant 3*D*/2D heterostructure achieves synergistic coupling of NCO’s hierarchical porosity with GNS’s conductive networks, creating multiscale transport pathways that simultaneously enhance catalytic activity through oxygen vacancy/defect engineering while ensuring rapid ionic/electronic conduction and oxygen diffusion kinetics. Crucially, this architectural design effectively mitigates carbon corrosion induced by Li_2_O_2_ deposition, a persistent challenge in nonaqueous battery systems (Figure 15d). Electrochemical evaluations demonstrate unprecedented bifunctional performance: remarkably low ORR and OER overpotentials surpassing commercial Pt/C benchmarks and single-component counterparts, coupled with a high specific capacity of 7201 mAh g^–1^ and exceptional cyclability of 200 stable cycles under 500 mAh g^–1^ capacity-limiting conditions. The durability of the hybrid system stems from dual protection mechanisms—the surface passivation effect of graphene synergizes with the robust framework of NCO to suppress electrolyte decomposition while maintaining structural integrity throughout prolonged cycling. This heterointerface engineering strategy establishes new design principles for next-generation cathode materials, balancing catalytic efficiency with electrochemical reversibility in oxygen-based energy storage architectures.

In contrast to the *in situ* construction of bimetallic MOFs, the ion-exchange approach toward bimetallic MOFs also serves as a feasible route to produce precursors for binary metal oxide composites.^181,182^ Zhang et al.^111^ developed hollow CeO_2_/Co_3_O_4_ polyhedra via a MOF-template strategy, using ZIF-67 as a precursor to introduce Ce^3+^ through ion exchange followed by high-temperature calcination (Figure 15e). Composed of nanoscale CeO_2_ and Co_3_O_4_ composite oxides, the material features hollow polyhedral structures with an average size of ∼ 200 nm, assembled from nanosheets and exhibiting uniform distribution of Ce, Co, and O elements to form unique core–shell heterointerfaces. The hollow porous architecture provides a high specific surface area and uniform mesopore distribution, facilitating oxygen diffusion, electrolyte infiltration, and storage of the discharge product Li_2_O_2_. The high oxygen ionic conductivity and reversible Ce^3+^/Ce^4+^ redox behavior of CeO_2_ endow it with an “oxygen pump” function, accelerating the nucleation and decomposition of Li_2_O_2_. Meanwhile, the bifunctional catalytic activity of Co_3_O_4_ toward ORR and OER works synergistically with CeO_2_ to significantly lower reaction overpotentials (discharge plateau ∼ 2.7 V, charge plateau ∼ 4.1 V) and optimize interfacial charge transfer. Experimental results show that this cathode delivers a high specific capacity of 9443 mAh g^–1^ at 100 mA g^–1^, maintains excellent rate performance with 7126 and 5100 mAh g^–1^ at 200 and 500 mA g^–1^, respectively, and achieves stable cycling of 140 and 90 cycles under fixed capacities of 600 and 1000 mAh g^–1^—far surpassing pure Co_3_O_4_ cathodes.

Beyond conventional MOF oxidation approaches, postoxidation strategies of carbonized MOFs have emerged as a sophisticated methodology for fabricating metal oxide composites with enhanced electronic conductivity through strategic carbon preservation. This approach ingeniously combines the merits of carbonaceous conductivity with catalytic metal oxide functionalities while simultaneously enabling selective removal of amorphous carbon domains to enhance graphitic content. A paradigm-shifting demonstration by Tang et al.^106^ developed cage-like graphitic porous carbon-Co_3_O_4_ (GPC-Co_3_O_4_) polyhedrons through sequential thermal processing of ZIF-8@ZIF-67 core–shell precursors (Figure 15f). The synthesis protocol involves initial high-temperature carbonization under nitrogen to create amorphous carbon@graphitic carbon–cobalt (AC@GC-Co) composites with distinct core–shell architecture—featuring cobalt-catalyzed graphitic carbon shells enveloping metallic cobalt nanoparticles. Subsequent air calcination leverages differential thermal stability between carbon phases, selectively eliminating amorphous core carbon while oxidizing residual cobalt into uniformly dispersed Co_3_O_4_ nanoparticles within preserved graphitic porous carbon frameworks. The resultant architecture exhibits three synergistic advantages: high surface area with hierarchical mesoporosity, internal cavity structures for Li_2_O_2_ accommodation, and conductive graphitic networks interwoven with catalytic Co_3_O_4_ domains. Electrochemical evaluations validate the design rationale, with GPC-Co_3_O_4_ cathodes delivering a substantial discharge capacity of 1575 mAh g^–1^ at 125 mA g^–1^ and exceptional capacity retention over 50 cycles under 500 mAh g^–1^ capacity-limiting conditions. This performance triad stems from graphitic carbon-enhanced electron transport, mesopore-facilitated mass transfer, and abundant Co^3+^/Co^2+^ active sites synergistically accelerating ORR and OER kinetics. In the following year, Song et al.^164^ developed a porous carbon/Co_3_O_4_ composite via a MOF-template strategy combined with postheat treatment (Figure 15g). The authors first synthesized a cobalt-based MOF using cobalt nitrate and adipic acid as precursors, followed by carbonization of the as-prepared MOF and subsequent oxidation in air at 280 °C. The mesopores within the carbon matrix provide ample storage space for Li_2_O_2_ and facilitate oxygen diffusion and electrolyte penetration, enabling the material to deliver a high discharge capacity of 9850 mAh g^–1^ at 100 mA g^–1^. SEM and XRD confirm the reversible deposition and decomposition of Li_2_O_2_ during charge and discharge processes, demonstrating that the carbon/Co_3_O_4_ composite effectively mitigates electrode passivation. Although these catalysts are not directly prepared by oxidizing MOF precursors and thus may not be strictly classified as MOF-derived metal oxides, this synthesis strategy efficiently generates highly dispersed metal oxide active sites to optimize their exposure and catalytic activity.

Studies have revealed that MOF precursors can be converted into metal oxides with inherited morphology and chemical composition without requiring heat treatment. Yin et al.^183^ synthesized a Prussian blue analogue (PBA, KCo­[Fe­(CN)_6_]) and utilized it to produce Co_3_O_4_ as a cathode catalyst for LOBs. In contrast to high-temperature processing, the MOF precursor reacts with NaOH at a lower temperature of 60 °C via an ion-exchange reaction: KCo­[Fe­(CN)_6_] + 2OH^–^ → Co­(OH)_2_ + [Fe­(CN)_6_]^3–^ + K^+^. The resulting Co_3_O_4_ catalyst not only preserves the cubic shape of the PBA precursor but also gradually develops porosity due to the ion-exchange reaction. Leveraging the common advantages of MOF-derived catalysts highlighted in previous examples, this Co_3_O_4_ catalyst exhibits an excellent discharge capacity of 4032 mAh g^–1^ at a current density of 0.08 mA cm^–2^ and stable performance over 170 cycles. More importantly, a key highlight of this work is the significantly reduced energy consumption in synthesizing the MOF-derived catalyst compared to conventional high-temperature treatments, representing an important consideration for future cost-effective catalyst production.

#### 4.1.4 MOF-Derived Metal Sulfides

Transition metal sulfides (TMSs) have garnered significant attention as advanced electrocatalysts, leveraging their distinctive layered architectures to provide abundant catalytically active sites that synergistically ameliorate ORR and OER kinetics while substantially reducing electrochemical polarization. Given the exceptional versatility in fabricating cathode materials with uniformly dispersed active sites and hierarchical architectures, MOFs hold great promise as desirable sacrificial templates for the preparation of metal sulfide cathodes for LOBs through heat treatment and sulfidation. Compared with conventional metal sulfides, MOF-derived metal sulfides possess abundant accessible active sites, facile mass and charge transport, and superior structural robustness, effectively boosting the ORR and OER kinetics, mitigating electrode pulverization, and prolonging operational lifespan. A paradigm-shifting demonstration by Dou et al.^168^ engineered cobalt sulfide-carbon porous nanocages (Co_9_S_8_@CPNs) through meticulous structural design (Figure 16a). The synthesis protocol involves cobalt ion coordination with 2-methylimidazole to form ZIF-67 precursors, followed by sequential thiourea-assisted sulfidation and high-temperature pyrolysis under an inert atmosphere. The resultant architecture features uniformly dispersed Co_9_S_8_ nanoparticles embedded within 3D carbon scaffolds, creating open nanocage structures with hierarchical porosity and surface areas exceeding conventional transition metal oxides. This configuration enables dual catalytic enhancement: Co^3+^/Co^2+^ redox-active centers synergize with conductive carbon networks to accelerate oxygen electrocatalysis, while the interpenetrating mesoporous channels facilitate rapid O_2_ and Li^+^ diffusion and provide interfacial space for Li_2_O_2_ accommodation. First-principles calculations reveal a strong LiO_2_ adsorption energy of – 5.192 eV on Co_9_S_8_ surfaces, which weakens O–O bonding and catalyzes Li_2_O_2_ nucleation kinetics through electron redistribution mechanisms (Figure 16b). This structural engineering breakthrough establishes new design principles for sulfur-based cathode catalysts that concurrently address conductivity limitations and interfacial stability challenges in metal–air battery systems. Inspired by the aforementioned strategies, Zhan et al.^51^ developed a CoS_2_@NC cathode material exhibiting exceptional performance (Figure 16c). The synthesis process involves initial pyrolysis of ZIF-67 at high temperature to generate Co@CNTs, followed by sulfidation treatment under high-temperature conditions to fabricate a nitrogen-coordinated CoS_2_@NC yolk–shell polyhedral catalyst. This material features CoS_2_ nanoparticles as the core and an NC as the shell, forming a unique yolk–shell hierarchically porous architecture. The yolk–shell structure provides ample space to buffer the volume change of the discharge product Li_2_O_2_, while the hierarchically porous characteristics facilitate O_2_ and electrolyte penetration, enhancing mass transport efficiency. Nitrogen-coordinated Co–N bonds and open metal sites optimize the electronic structure, strengthening O_2_ adsorption and activation capabilities to enable efficient bifunctional catalysis (ORR and OER) and reduce overpotentials. Beyond single-metal cobalt-based sulfides, Liu et al.^169^ successfully synthesized a porous C/MoS_2_–CoMo_2_S_4_ composite comprising a carbon matrix, MoS_2_ nanosheets, and CoMo_2_S_4_ nanotubes via a strategy involving calcination of ZIF-67 with a C/MoS_2_ composite at 800 °C, followed by acid washing. In this material, the carbon skeleton suppresses agglomeration of MoS_2_ and enhances electrical conductivity, while CoMo_2_S_4_ provides abundant bimetallic active sites. Acid washing removes low-activity free cobalt particles, further exposing highly catalytic CoMo_2_S_4_. The synergistic effect between CoMo_2_S_4_ and MoS_2_, combined with the uniform distribution of single-atom cobalt, significantly boosts the catalytic efficiency of ORR and OER reactions. Meanwhile, the 3D porous network structure of the material not only establishes fast diffusion channels for Li^+^ and O_2_ but also provides ample space for storage and reversible decomposition of the discharge product Li_2_O_2_. Experimental results demonstrate that the acid-washed C/MoS_2_–CoMo_2_S_4_-800 electrode achieves 336 cycles of stable cycling at a current density of 500 mA g^–1^, with a high specific discharge capacity of 17551 mAh g^–1^ and an overpotential as low as 1.18 V.

**Figure 16:**
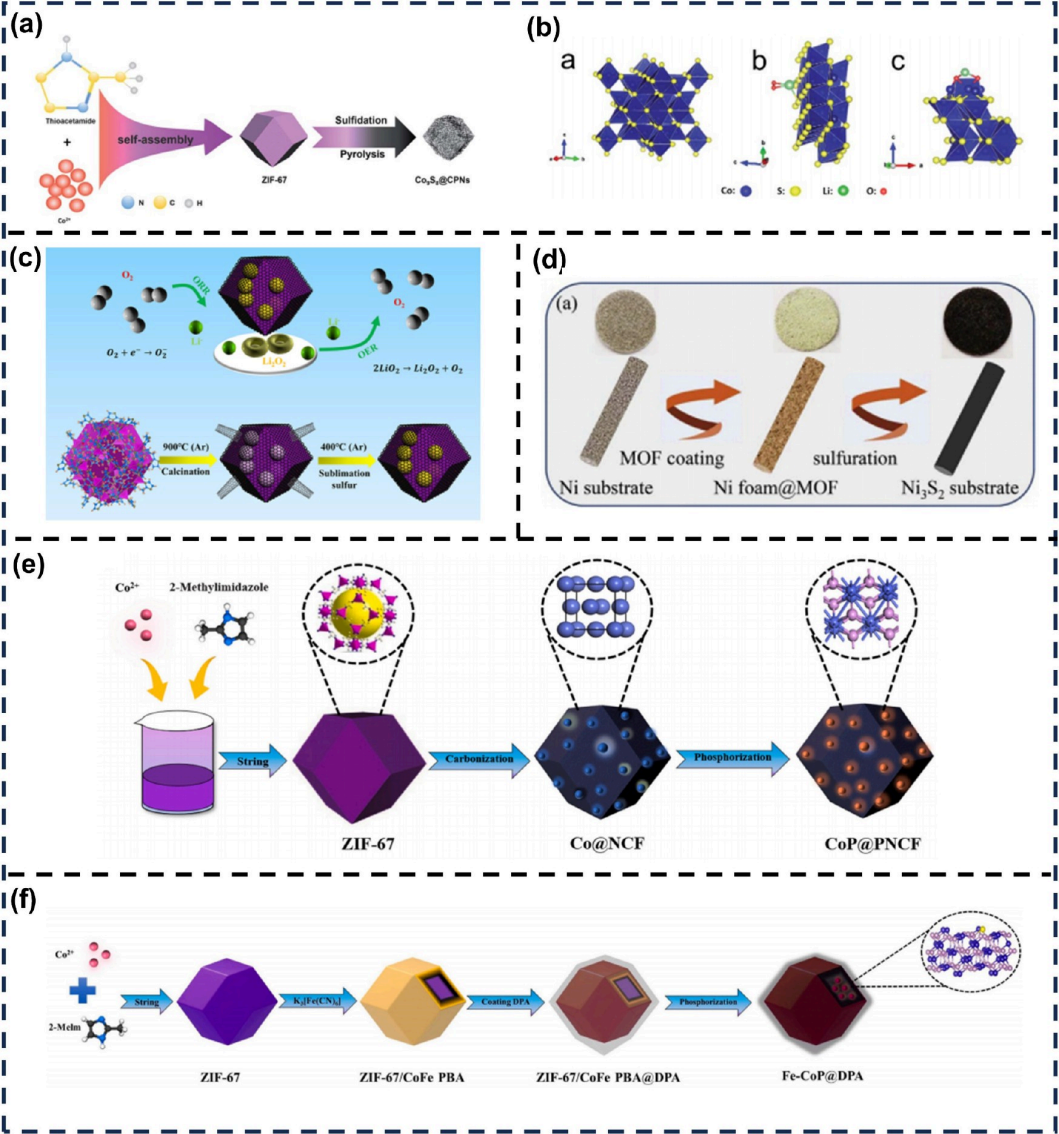
(a) Schematic illustration of the formation process and (b) DFT calculation results of Co_9_S_8_@CPNs. Reproduced with permission from ref 168. Copyright 2018 Royal Society of Chemistry. (c) Schematic illustration of the reaction mechanism using CoS_2_(CoS)@NC as the O_2_ electrodes of LOBs, and the preparation process of CoS_2_(CoS)@NC. Reproduced with permission from ref 51. Copyright 2021 American Chemical Society. (d) Schematic illustration of the synthesis process of the Ni_3_S_2_ substrate. Reproduced with permission from ref 171. Copyright 2020 Elsevier. (e) Schematic illustration of the fabrication of CoP@PNCF. Reproduced with permission from ref 172. Copyright 2023 Elsevier. (f) Schematic illustration of the fabrication of Fe-CoP@DPA catalyst. Reproduced with permission from ref 173. Copyright 2024 Elsevier.

The robust interfacial coupling between metal sulfides and substrate matrices significantly enhances charge transfer kinetics while minimizing interfacial impedance. The employment of MOFs as sacrificial templates also enables the rational fabrication of an integrated metal sulfide cathode for LOBs. Wang et al.^171^ developed a templated sulfurization strategy using Ni-MOF precursors to fabricate porous Ni_3_S_2_ architectures seamlessly integrated on 3D nickel foam substrates, serving dual functions as both current collector and catalytic layer (Figure 16d). This integrated 3D foam-type cathode demonstrated remarkable electrochemical performance improvements, achieving a 3-fold enhancement in discharge capacity and cycling stability compared to pristine Ni foam, attributed to its unique porous configuration and homogeneous regulation of surface electrochemical processes. Crucially, the discharge products exhibited well-defined lamellar architecture with extended radial dimensions, while the Ni_3_S_2_ catalytic layer effectively guided LiO_2_ formation pathways and suppressed charge overpotential. Experimental results demonstrate that the Ni_3_S_2_-based cell delivers an exceptional specific discharge capacity of 7478 mAh g^–1^ at 100 mA g^–1^ with a low overpotential of 1.36 V, and maintains a stable capacity of 500 mAh g^–1^ even under high current densities up to 2000 mA g^–1^. Furthermore, the cycling durability substantially outperforms conventional Ni substrates, sustaining 120 stable cycles at 300 mA g^–1^ with a capacity limitation of 600 mAh g^–1^. This performance enhancement originates from the optimized ion/electron transport pathways and reduced polarization effects enabled by the precisely regulated lamellar morphology of discharge products.

#### 4.1.5 MOF-Derived Metal Selenides

To date, transition metal oxides (TMOs) and TMSs have been developed as ORR and OER catalysts for LOBs due to their high activity, low cost, and tunable composition and morphology. However, the intrinsic electron-insulating nature of most TMOs limits their catalytic performance. Compared with oxygen and sulfur elements, selenium (Se) possesses a larger atomic radius, lower ionization energy, and abundant d-electrons, which facilitate charge transfer.^184,185^ Moreover, selenium atoms in selenides endow metal centers with enhanced electrophilicity, strengthening the affinity of selenides toward oxygen and hydroxyl groups.^186,187^ These features render transition metal selenides promising candidates for OER catalysis. Liang et al.^188^ developed a 3D self-supported Co-CoSe@NSeC/bioC cathode material via *in situ* growth of cobalt-based ZIF-67 on biomass (pomelo peel), followed by high-temperature selenization. The material consists of cobalt/cobalt selenide (Co/CoSe) heterojunction nanoparticles embedded in a nitrogen/selenium codoped carbon skeleton, where biomass-derived carbon provides a natural 3D porous architecture. The ZIF-67-derived Co/CoSe heterojunctions synergize with doped N and Se atoms to enhance catalytic activity: the 3D porous structure promotes rapid transport of oxygen and electrolyte while providing ample storage space for the discharge product Li_2_O_2_; the N, Se codoped carbon framework introduces abundant defect sites to optimize charge distribution and oxygen adsorption, whereas Co/CoSe heterojunctions accelerate ORR and OER kinetics through interfacial charge reconstruction. Experimental results show that this cathode delivers a high specific capacity of 9.1 mAh cm^–2^ at 0.05 mA cm^–2^ with a low charge–discharge overpotential of 0.73 V and maintains stable cycling for 78 cycles, significantly outperforming the unmodified Se-bioC material. The excellent performance of Co-CoSe@NSeC/bioC highlights its potential as a cathode material for LOBs.

#### 4.1.6 MOF-Derived Metal Phosphides

Extensive research has demonstrated that developing efficient bifunctional catalysts can effectively mitigate the large overpotential gap in LOBs and enhance their cycling efficiency.^189^ Many transition metal phosphides (TMP) exhibit high electrocatalytic activity due to their excellent electrical conductivity and diverse bonding configurations, making their coupling with heteroatom-doped carbon materials a promising direction for LOB applications. As a TMP catalyst with negatively charged phosphorus centers, CoP efficiently promotes the adsorption/desorption of oxygen intermediates, emerging as an electrode material with extensive research. However, challenges remain in optimizing synthesis techniques and unraveling the electrocatalytic mechanisms of CoP-based electrocatalysts. Zhang et al.^172^ addressed this by regulating the temperature, using ZIF-67 as a precursor to fabricate a bifunctional electrocatalyst with CoP nanoparticles anchored in phosphorus/nitrogen codoped carbon nanoframes (CoP@PNCFs) via high-temperature pyrolysis and treatments (Figure 16e). The material consists of uniformly dispersed CoP nanoparticles embedded in a defect-rich P/N codoped carbon skeleton, where high-content pyridinic and graphitic nitrogen optimize the electronic structure, while phosphorus-doped carbon synergizes with CoP active sites to significantly enhance conductivity and catalytic activity. When applied in LOBs, CoP@PNCF-700 (phosphidated at 700 °C) demonstrates exceptional performance: its high specific surface area (139.20 m^2^ g^–1^) and hierarchical porous structure facilitate uniform Li_2_O_2_ deposition and efficient decomposition, while bifunctional ORR and OER activity reduces charge and discharge overpotentials. Notably, the CoP@PNCF-700-based battery delivers a high specific discharge capacity of 9630.5 mAh g^–1^ at 100 mA g^–1^ and exhibits outstanding long-cycle stability with 187 cycles under a fixed capacity of 500 mAh g^–1^ at 200 mA g^–1^. Subsequently, Zhang et al.^173^ developed a bifunctional electrocatalyst with hollow core–shell architecture through a one-step phosphidation strategy, employing ZIF-67 precursors integrated with *in situ* formed CoFe Prussian blue analogues and polydopamine-derived carbon encapsulation (Figure 16f). The resulting Fe-doped CoP nanoparticles confined within phosphorus/nitrogen-co-doped carbon matrices (denoted as Fe-CoP@DPA) feature Fe-CoP nanocores enveloped by hollow carbon carriers. The incorporation of Fe synergistically modulates the electronic configuration of CoP while the polydopamine-derived coating preserves active sites, collectively establishing hierarchical porosity and enhanced surface area to optimize Li^+^/O_2_ diffusion kinetics and electron transport pathways. DFT calculations reveal that Fe doping induces an upward shift in the d-band center of Fe-CoP@DPA, thereby strengthening intermediate adsorption during catalytic processes. The LOB equipped with this architecturally refined Fe-CoP@DPA cathode demonstrates exceptional electrochemical performance, delivering an extended cycle life of 282 cycles with a fixed capacity of 500 mAh g^–1^ at 200 mA g^–1^, coupled with an ultrahigh specific discharge capacity of 18208.5 mAh g^–1^ at 100 mA g^–1^. Mechanistic analysis indicates these superior properties originate from the hollow structure’s effectiveness in mitigating Li_2_O_2_ accumulation while facilitating its reversible decomposition, complemented by the protective polydopamine-derived carbon shell that suppresses parasitic reactions and stabilizes active sites. In addition to the metal-doped phosphide catalysts, MOFs have also been employed as templates to fabricate bimetallic phosphide catalysts with hollow architectures to achieve high surface area and superior structural robustness. In a recent advancement, Zhang et al.^174^ research team engineered a hollow nanostructured NiCo bimetallic phosphide catalyst (NiCoP@PNCF) for cathode applications through a polydopamine-mediated coating strategy. The synergistic interplay between heteroatom-doped carbon matrices and NiCoP nanoparticles endows the hollow-structured NiCoP@PNCF cathode with abundant exposed active sites, which substantially accelerates ion transport efficiency and enhances bifunctional reaction kinetics. Remarkably, DFT calculations provide fundamental insights into the underlying mechanisms governing the improved electrical conductivity and electrochemical activity. Capitalizing on these structural and compositional merits, the LOBs incorporating NiCoP@PNCF catalysts demonstrate outstanding electrochemical performance, delivering a high specific discharge capacity of 13070.2 mAh g^–1^ at 200 mA g^–1^ coupled with extraordinary cycling stability of 242 cycles at a fixed specific capacity of 500 mAh g^–1^. This work provides valuable insights for designing advanced phosphide-based electrocatalysts and paves the way for developing high-efficiency cathode materials in metal air battery systems.

### 4.2 Other Applications

Lithium metal anodes have emerged as a focal point in LOBs research owing to their exceptional energy density, driven by the inherent properties of metallic lithium. Boasting an ultralow electrochemical potential of – 3.04 V relative to the reversible hydrogen electrode and an extraordinary theoretical specific capacity of 3860 mAh g^–1^, lithium metal represents a highly promising anode material.^190^ Nevertheless, its practical deployment is impeded by critical challenges such as uncontrolled lithium dendrite growth and severe volume expansion stemming from inhomogeneous lithium deposition, both of which raise substantial safety concerns. Recent studies underscore the efficacy of 3D conductive frameworks in mitigating localized current density during lithium plating. Consequently, achieving uniform lithium distribution and optimizing nucleation kinetics are pivotal for curbing dendritic growth. Addressing this, Zhao et al.^191^ engineered zinc–cobalt codoped nitrogen-enriched porous carbon nanocubes (Zn/Co–N@PCN) via pyrolysis of bimetallic Zn/Co zeolitic imidazolate frameworks. Carbonization at 800 °C under argon yielded a 3D porous carbon matrix, where Zn evaporation generated meso/macropores, while residual Co nanoparticles coupled with nitrogen species from ligand decomposition formed lithiophilic active sites. The Zn/Co–N@PCN architecture exhibits a high specific surface area of 342.3 m^2^ g^–1^, hierarchical porosity, and uniformly dispersed Co/Zn–N moieties, synergistically enhancing electrical conductivity and structural integrity. As a lithium host, the porous framework alleviates electrode swelling, while the Co/Zn–N sites reduce lithium nucleation overpotential to a mere 2 mV, enabling homogeneous deposition and dendrite suppression. Symmetric cells incorporating Zn/Co–N@PCN demonstrate a Coulombic efficiency of 99.0% over 600 cycles at 0.5 mA/cm^–2^, while Li/Li cells maintain stable operation for 200 h at 1 mA cm^–2^ with an ultralow overpotential of 60 mV. This work exemplifies a dual optimization of lithiophilicity and mechanical stability in carbon-based hosts through bimetallic coordination and pore engineering, offering a transformative design paradigm for high-energy-density LOBs with enhanced safety and cyclability. In recent years, MOF-based materials have made significant progress in protecting lithium anodes for LOBs. The main mechanisms include: using ordered pores and sieving effects as separator coatings or artificial SEI layers to homogenize Li^+^ flux and suppress dendrite growth; polar sites adsorbing impurities and harmful intermediates to reduce anode corrosion; good mechanical strength inhibiting volume expansion and reducing “dead Li”; and participating in the formation of stable, highly conductive SEI films to lower interfacial impedance and improve deposition/stripping reversibility.

### 4.3 Summary

MOF derivatives, engineered through directed transformation strategies, reconstruct the materials genome of key components in LOBs. The derivation process constructs composite systems comprising hierarchically porous carbon-based frameworks and metal compounds. These systems synergistically optimize the bicontinuous transport network for electrons and ions. The atomically dispersed active sites significantly reduce the energy barriers for oxygen electrocatalytic reactions and enable the reversible conversion of discharge products by modulating interfacial adsorption energies, thereby elevating the energy conversion efficiency to unprecedented levels. Through precise cross-scale structural regulation and innovative device integration, MOF derivatives are poised to empower LOBs to surpass the theoretical limits of energy density and cycle life, laying a robust material foundation for next-generation ultrahigh-specific-energy storage systems.

## 5 MOF Composites

The intrinsically low conductivity of most MOFs arises from their structural dependence on directional and saturated coordination bonds between metal nodes and organic ligands, which severely restrict electron delocalization and mobility within the framework. Additionally, the electronic and band structures of MOFs are inherently unfavorable for efficient electron hopping, while their porous architectures are often occupied by insulating guest molecules, further compromising bulk conductivity. To address these limitations, integrating MOFs with conductive functional matrices has emerged as a transformative strategy. Notable examples include hybridization with high-conductivity one-dimensional (1D) CNTs or carbon nanofibers (CNFs), 3D freestanding carbon cloth (CC), and emerging 2D materials such as MXenes.^192–194^ The key advantage of MOF composites lies in their synergy with conductive matrices, enabling a balanced integration of electron transport, ion diffusion, and catalytic activity. This makes them highly suitable for developing high-rate, high-energy-density, and practically applicable devices. These composite systems synergistically combine the structural versatility of MOFs with the superior electronic properties of conductive substrates, effectively overcoming conductivity bottlenecks. Recent research has thus prioritized the rational design of MOF-based composites, unlocking their potential as advanced electrocatalysts with enhanced charge transfer kinetics, catalytic efficiency, and long-term stability for next-generation energy technologies (Table 3).

**Table 3:** Summary of MOF Composites for LOBs

MOFs	Sample	Discharge capacities (mAh g^–1^)	Cycle number	refs
Mn-MOF-74	Mn-MOF-74@CNTs	2500 (125 mA g^–1^)	60 (125 mA g^–1^)	^195^
ZIF-8	MOF-C/CNT	10050 (50 mA g^–1^)	75 (200 mA g^–1^)	^48^
MOF(Fe/Co)	MOF(Fe/Co)-CNTs-600	5000 (100 mA g^–1^)	40 (100 mA g^–1^)	^196^
PAN/ZIF-8/ZIF-67	Co@N-CNF	9290.7 (50 mA g^–1^)	42 (100 mA g^–1^)	^197^
Mn-MOF	MnOC@CC	22838 (200 mA g^–1^)	53 (200 mA g^–1^)	^198^
Co-MOF	Co_3_O_4_@NiCo_2_O_4_/CC	10645 (100 mA g^–1^)	225 (100 mA g^–1^)	^47^
CMT	CMT@MXene	6849.16 (200 mA g^–1^)	247 (500 mA g^–1^)	^46^
Co-MOF	Co-MOF/MXene	34763 (1000 mA g^–1^)	278 (1000 mA g^–1^)	^199^

### 5.1 Composite with CNT or CNF

CNTs, characterized by their 1D hollow tubular structure with exquisite crystallinity, exhibit near-metallic electronic conductivity and exceptional mechanical robustness. These attributes enable CNTs to construct highly efficient electron-conducting networks, mitigate electrode volume expansion, and significantly enhance ORR and OER kinetics through strategic doping or catalyst loading.^200–202^ In contrast, CNFs feature porous fibrous architectures with abundant surface defects, where their 3D interwoven networks and high porosity optimize oxygen diffusion pathways and promote uniform Li_2_O_2_ deposition. Moreover, CNFs offer advantages such as cost-effectiveness, intrinsic flexibility, and facile functionalization via scalable techniques like electrospinning, making them highly compatible with large-scale electrode fabrication.^203,204^ As two distinct carbon allotropes, both CNTs and CNFs are widely recognized for their outstanding mechanical strength, superior conductivity, and exceptional chemical and electrochemical stability, positioning them as pivotal materials for advancing LOBs technologies.^205,206^ Zhang et al.^195^ developed a composite catalyst, Mn-MOF-74@CNTs, through an additive-mediated synthesis strategy, wherein Mn-MOF-74 nanoparticles were *in situ* grown on CNTs (Figure 17a). This architecture leverages CNTs as a conductive scaffold to support highly porous Mn-MOF-74 nanoparticles, synergistically combining the catalytic activity of open metal sites (Mn^2+^/Mn^3+^) in MOFs with the 3D conductive network of CNTs. In LOBs, this composite material demonstrates a unique chemical catalytic mechanism under humid oxygen environments containing 200 ppm water vapor. It effectively converts conventional discharge product Li_2_O_2_ into less reactive LiOH, thereby suppressing parasitic reactions such as electrolyte decomposition and carbon corrosion while significantly enhancing cycling stability. Specifically, the Mn^2+^/Mn^3+^ sites within Mn-MOF-74 catalyze the reaction between Li_2_O_2_ and H_2_O to form LiOH, concurrently accelerating H_2_O_2_ decomposition to maintain reaction equilibrium. Simultaneously, the high conductivity of CNTs reduces electrode impedance, while their mechanical robustness buffers volume fluctuations during cycling. By tailoring the chemical pathways of discharge products and integrating MOF catalysis with carbon-based conductive networks, this work provides a robust cathode design strategy for LOBs operating in high-humidity environments, paving the way for practical applications.

**Figure 17:**
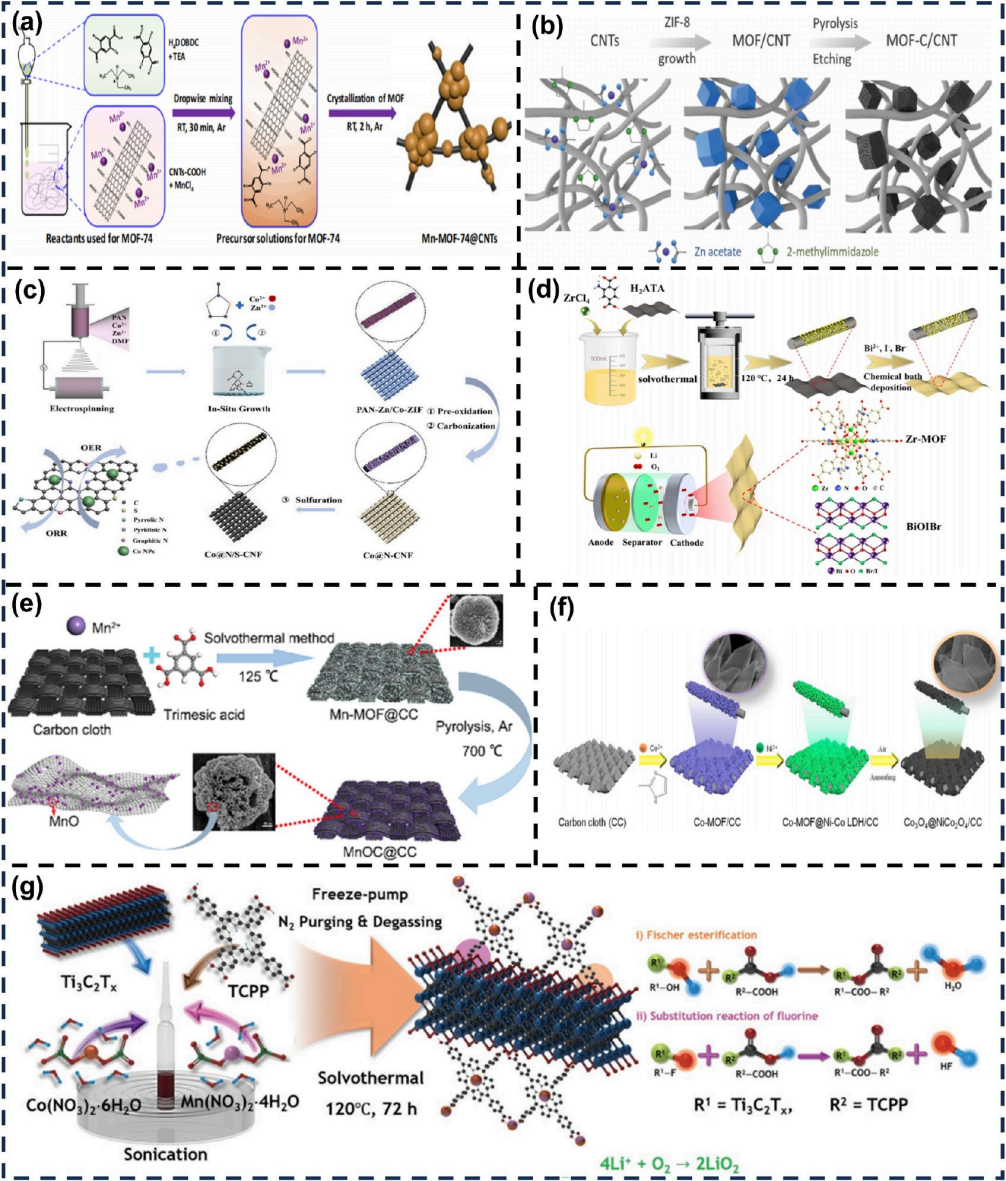
(a) Schematic of the synthesis protocol of Mn-MOF-74@CNTs hybrid. Reproduced with permission from ref 195. Copyright 2019 Elsevier. (b) Schematic diagram illustrating the synthesis process of dual-phasic MOF-C/CNT nanoarchitecture. Reproduced with permission from ref 48. Copyright 2019 Wiley-VCH. (c) Schematic illustration of the synthesis of Co@N/S-CNF. Reproduced with permission from ref 197. Copyright 2022 Elsevier. (d) Schematic diagram of the preparation of Zr-MOF/BiOIBr/CC. Reproduced with permission from ref 209. Available under a CC-BY 3.0 license. Copyright 2024 Royal Society of Chemistry Tao et al. (e) Illustration of the overall preparation process of the MnOC@CC cathode. Reproduced with permission from ref 198. Copyright 2023 American Chemical Society. (f) Schematic diagram of the process for preparing the Co_3_O_4_@NiCo_2_O_4_/CC cathode. Reproduced with permission from ref 47. Copyright 2020 Elsevier. (g) Synthetic routes of CMT@MXene with proposed chemical structures, freeze–thaw cycles to remove oxygen elements and other dissolved gases, and solvothermal reaction for the growth of CMT MOF via (i) Fischer esterification and (ii) substitution reaction of fluorine between CMT and MXene. Reproduced with permission from ref 46. Copyright 2023 Wiley-VCH.

To prevent self-aggregation of catalytic nanoparticles and further enhance electron transfer, integrating CNT or CNF conductive matrices with MOF derivatives has emerged as a promising strategy. Pham et al.^48^ engineered a dual-phase carbon nanostructure (MOF-C/CNT) through a hydrothermal synthesis coupled with carbonization and chemical etching, wherein ZIF-8 nanoparticles were *in situ* grown on CNTs (Figure 17b). This architecture integrates MOF-derived porous carbon (MOF-C) with conductive CNTs, forming an interwoven network. The MOF-C component, derived from carbonization, retains a high specific surface area and large pore volume, providing abundant active sites for oxygen redox reactions and efficient Li_2_O_2_ storage. Simultaneously, the CNTs construct a 3D conductive framework that facilitates rapid electron transfer and oxygen diffusion while suppressing particle agglomeration of MOF-C through their interlaced structure, thereby enhancing mechanical robustness. This synergistic design not only addresses the limitations of standalone MOF-derived carbons but also establishes a cathode material with exceptional electrochemical performance and structural durability for advanced LOBs. Wang et al.^196^ prepared a bifunctional electrocatalyst with FeCo alloy core@NC shell nanoparticles anchored on CNTs via pyrolysis of a FeCo bimetallic MOF­(MOF­(Fe/Co))-CNT composite. The material features FeCo alloy cores encapsulated by NC shells, which are robustly bonded to CNTs to form a 3D conductive network. The synergistic effect of the FeCo alloy optimizes the adsorption energy of oxygen species, while the NC shells provide abundant active sites and enhance electron conduction. Meanwhile, CNTs suppress agglomeration of alloy nanoparticles and create channels for electrolyte diffusion and electron transport. When applied in LOBs, this catalyst exhibits superior bifunctional ORR and OER activity in alkaline electrolytes with a low overpotential difference (Δ*E*) of 0.79 V, approaching the performance of noble metal catalysts. As a battery cathode, it delivers initial discharge/charge capacities exceeding 5000 mAh g^–1^, maintains 40 cycles of stable cycling under a capacity limit of 500 mAh g^–1^, and features a narrow charge and discharge voltage gap of 1.55 V, outperforming most non-noble metal catalysts. These advantages arise from the synergistic catalysis of FeCo alloy and NC, the stabilizing effect of CNTs on active sites, and the hierarchical pores facilitating rapid transport of oxygen and electrolyte. Guo et al.^197^ developed a cathode catalyst with cobalt nanoparticles embedded in nitrogen/sulfur codoped porous carbon nanofibers (Co@N/S-CNF) via an electrospinning strategy combined with bimetallic ZIF-8/ZIF-67 precursors (Figure 17c). The synthesis process involves initial *in situ* growth of ZIF-8/ZIF-67 core–shell structures on PAN/Zn/Co nanofibers, followed by preoxidation and carbonization to obtain nitrogen-doped CNFs anchored with cobalt nanoparticles (Co@N-CNF). Sulfur is then introduced through a thioacetamide hydrothermal reaction and calcination, resulting in a composite structure of 1D porous N/S codoped CNFs supporting cobalt nanoparticles. In this material, cobalt nanoparticles are encapsulated by graphitized carbon layers and uniformly distributed on the fiber surface. N/S codoping introduces structural defects and active sites via electronegativity and size effects, while the mesoporous structure of the fibers facilitates oxygen and electrolyte diffusion. Compared with single-doped materials, N/S codoping significantly enhances battery performance by improving oxygen adsorption and reversible product decomposition. This work provides a new strategy for developing low-cost, high-activity bifunctional catalysts for LOBs through MOF-derived synthesis and precise heteroatom regulation.

### 5.2 Composite with Carbon Cloth

CC, a 3D porous architecture fabricated through carbon fiber weaving or nonwoven processing, demonstrates exceptional structural merits including hierarchical porosity, interwoven conductive networks, substantial specific surface area, and remarkable mechanical flexibility. Capitalizing on these advantageous characteristics, researchers have successfully integrated MOFs into carbon matrices to construct composite cathodes for LOBs.^207,208^ Tao et al.^209^ developed an innovative heterostructured catalyst through sequential growth of Zr-MOF octahedral particles and bismuth oxyhalide (BiOIBr) nanosheets on acid-treated CC substrates via solvothermal and chemical bath deposition strategies (Figure 17d). This hierarchical architecture synergistically combines the conductive carbon framework with Zr-MOF-derived nitrogen-doped active sites and BiOIBr-enhanced visible-light absorption sites. The established built-in electric field at the heterojunction interface effectively promotes spatial separation and directional transport of photogenerated charge carriers. Consequently, the photoassisted LOB employing this Zr-MOF/BiOIBr/CC cathode achieves remarkable electrochemical performance, exhibiting a discharge potential of 3.05 V, ultralow charge potential of 3.20 V, and exceptional energy efficiency reaching 95.3%, along with extended cyclability over 255 cycles. Notably, the interfacial electric field simultaneously mitigates passivation effects from insulating Li_2_O_2_ layers while regulating the growth morphology of discharge products toward film-like deposition rather than conventional sheet formation, thereby enhancing reaction reversibility. Furthermore, Huang et al.^198^ developed a free-standing MOF-derived honeycomb-like MnO/C composite anchored on carbon cloth (denoted as MnOC@CC) through an innovative strategy involving *in situ* growth of urchin-like Mn-MOFs followed by controlled carbonization (Figure 17e). This hierarchical architecture features uniformly distributed MnO–amorphous carbon hybrid units forming ordered hexagonal channels, which are firmly integrated with the conductive carbon matrix to establish 3D charge transport pathways. The unique honeycomb configuration not only facilitates rapid electrolyte penetration and directional transport of Li^+^ and O_2_ species but also enables the ordered arrangement of Mn–O catalytic centers. These active sites effectively regulate the discharge product evolution from highly reactive Li_2_O_2_ to more stable LiOH, generating flower-like LiOH nanosheets that enhance electrode–electrolyte interface stability while enabling efficient decomposition of discharge products during charging processes. Consequently, the MnOC@CC-based LOB delivers extraordinary electrochemical performance, achieving an exceptional discharge specific capacity of 22838 mAh g^–1^ at 200 mA g^–1^ and retaining 9119 mAh g^–1^ at an elevated current density of 800 mA g^–1^. Remarkably, the system demonstrates outstanding cyclability with 53 stable cycles at 500 mAh g^–1^ cutoff capacity, accompanied by low charge-transfer resistance and minimal byproduct formation. This innovative configuration synergistically combines structural advantages from the MOF-derived porous framework with bifunctional catalytic activity and stable conductive pathways, enabling reversible LiOH formation/decomposition and significantly enhancing energy density, rate capability, and cycling stability of LOBs.

Additionally, composite materials prepared by *in situ* growth of MOFs on CC have also been employed as precursors for constructing integrated cathodes with mixed-valence TMOs. Li et al.^47^ demonstrated a breakthrough in LOBs technology through the development of freestanding Co_3_O_4_@NiCo_2_O_4_/CC composite cathodes (Figure 17f) by employing an advanced MOF-templated synthesis strategy. The researchers fabricated sword-like nanoarray architectures via *in situ* growth of Co-MOF nanosheet arrays on CC substrates, followed by nickel nitrate impregnation and controlled pyrolysis under air flow. The resulting hierarchical structure features a core–shell configuration with inner Co_3_O_4_ layers seamlessly encapsulated by outer NiCo_2_O_4_ phases, forming 3D sword-like nanoarrays firmly anchored on the carbon matrix. This architecture integrates multiple advantageous characteristics, including interconnected porosity, abundant oxygen vacancies, and binder-free electrode configuration. The vertically aligned nanoarray morphology establishes open macroporous channels that facilitate efficient oxygen diffusion and electrolyte infiltration while providing sufficient space for Li_2_O_2_ deposition and decomposition. Simultaneously, the interconnected conductive network ensures rapid electron transfer and ion transport kinetics. Notably, the binder-free design eliminates parasitic reactions between conventional polymeric binders and electrolytes, while the engineered oxygen vacancies synergistically enhance catalytic activity through increased active site exposure and optimized surface reactivity. Electrochemical evaluations reveal extraordinary performance metrics: a record discharge specific capacity of 10645 mAh g^–1^ at 100 mA g^–1^, extended cyclability over 225 at a cutoff capacity of 500 mAh g^–1^, and remarkably low charge-transfer resistance compared to monometallic oxide counterparts. These achievements stem from the strategic integration of structural engineering and defect chemistry, offering new perspectives for designing high-performance LOBs.

### 5.3 Composite with MXenes

Beyond conventional carbon matrices such as CNT networks and CC, emerging 2D transition metal carbides (MXenes) have demonstrated remarkable potential in enhancing the functionality of MOFs.^210^ The recent emergence of MXenes as a novel class of 2D materials has attracted considerable scientific interest owing to their unique hexagonal close-packed lattice symmetry and exceptional metallic character. These materials exhibit unprecedented compositional tunability through solid solution formation, enabling precise electronic structure engineering critical for catalytic applications. The characteristic multiatomic layered architecture of MXenes facilitates rapid electron transfer to active sites via metallic conductivity, effectively lowering activation energy barriers and accelerating ion diffusion kinetics. Of particular interest is Ti_3_C_2_ MXene, whose unique combination of structural anisotropy and surface chemistry has established it as a premier cathode material candidate for LOBs.^20,211,212^ The synergistic integration of MXenes with MOFs creates heterointerfaces that optimize electron delocalization while preserving structural integrity, offering new pathways to overcome intrinsic limitations in conventional composite electrodes. Pioneering work by Nam et al.^46^ demonstrated a novel synthetic protocol for constructing bimetallic cobalt/manganese organic framework (CMT) architectures directly on Ti_3_C_2_T_*x*_ MXene substrates through solvent-assisted thermal assembly (Figure 17g). The innovative design leverages ligand-mediated interfacial engineering, where carboxyl groups from tetracarboxyphenyl porphyrin (TCPP) form robust chemical linkages with hydroxyl and fluorine terminations on MXene surfaces via Fischer esterification coupled with fluorine substitution mechanisms. This bottom-up assembly yields CMT@MXene hybrid electrocatalysts featuring metalloporphyrin frameworks chemically anchored to conductive MXene nanosheets. The atomic-level integration creates synergistic effects: cobalt/manganese nodes establish coordination bonds with TCPP ligands for optimized d-orbital electron configuration, while the MXene substrate ensures rapid charge transfer through its delocalized π-electrons and engineered oxygen vacancies. As a bifunctional oxygen catalyst, the composite demonstrates exceptional activity metrics with remarkably low overpotentials of 0.22 V for ORR and 0.97 V for OER. The system achieves a high specific capacity of 6849.16 mAh g^–1^ while maintaining outstanding cycling stability through 247 cycles at 500 mA g^–1^ with 1000 mAh g^–1^ capacity limitation. Notably, surface ligand functionalization significantly enhances MXene oxidation resistance, enabling unprecedented stability across acidic, alkaline, and organic electrolytes-a critical advancement addressing the long-standing challenge of MXene degradation in operational environments. This molecular engineering strategy successfully circumvents the inherent limitations of conventional MXenes and noble metal catalysts, offering a sustainable pathway for durable, high-performance energy conversion systems. Zhang et al.^199^ engineered a freestanding flexible Co-MOF/MXene heterostructured film as an advanced cathode catalyst for LOBs, demonstrating exceptional ORR catalytic efficiency. This hierarchically structured hybrid material features vertically aligned Co-MOF nanosheets intercalated between Ti_3_C_2_T_*x*_ MXene layers, creating 2D charge transport channels that effectively suppress MXene self-restacking while enabling rapid Li^+^ and O_2_ flux. The atomic-level interfacial coupling between metalloorganic frameworks and MXene substrates optimizes electron delocalization, significantly lowering the energy barrier for Li_2_O_2_ nucleation and decomposition. Remarkably, this design paradigm deviates from conventional slurry-based cathode manufacturing processes, representing a groundbreaking approach in next-generation battery architecture. When deployed in LOBs, the Co-MOF/MXene cathode delivers extraordinary electrochemical performance, achieving a record-breaking specific capacity of 34763 mAh g^–1^ at 1000 mA g^–1^ while maintaining 278 stable cycles under identical current density. The synergistic combination of framework porosity, MXene conductivity, and interfacial charge redistribution establishes new benchmarks for a high-energy-density metal-air battery.

### 5.4 Summary

MOFs and their derivatives have significantly enhanced LOB performance through precise hybridization with multidimensional functional substrates. 1D carbon-based matrices (CNTs, CNFs) construct efficient electron transport networks via their high conductivity and mechanical strength, while buffering volume strain. Their porous architectures optimize oxygen diffusion and discharge product deposition, with surface modifications (e.g., heteroatom doping) or MOF-derived catalytic sites synergistically reducing oxygen reaction energy barriers to enable low-overpotential charge–discharge operations. 3D self-supported CC integrates MOF active components through its high porosity and flexible framework; its binder-free structure avoids side reactions, and open channels facilitate mass and charge transport. Additionally, photo/electrocatalytic heterojunction designs can regulate discharge product morphology and decomposition pathways, improving cycling reversibility.^213,214^ The 2D MXenes, relying on ultrahigh conductivity and surface tunability, enhance MOF-substrate interface stability via coordination bonding, suppress interlayer stacking to accelerate ion and gas transport, and overcome the antioxidative degradation limitations of traditional materials. Overall, MOF-substrate composites have successfully broken through the intrinsic constraints of LOBs in energy density and cycle life through cross-scale structural engineering and functional synergy.^215–217^

## 6 Conclusions and Outlook

This comprehensive review critically examines recent theoretical and experimental advancements in MOFs for LOBs applications, systematically analyzing their evolving roles as multifunctional components in next-generation energy storage systems. MOFs demonstrate unparalleled advantages in structurally tailored hierarchical porosity, atomically dispersed catalytic centers, and synergistic integration with conductive matrices, collectively enabling enhanced mass and charge transfer kinetics, optimized redox activity, and superior cycling durability. Their versatile implementation spans critical battery components, including cathodes, anodes, separators, and solid-state electrolytes, where MOF-based architectures facilitate accelerated charge transfer, improved reaction thermodynamics, and significantly reduced polarization. The past decade has witnessed remarkable progress in MOF-based functional materials, with numerous structural variants—including pristine MOFs, their derivatives (Carbon-based matrices, metallic species, metal oxides/sulfides/selenides/phosphides), and hybrid composites (MOF/CNT, MOF/CNF, MOF/CC, MOF/MXene)—demonstrating exceptional potential as advanced key materials for LOBs. Our analysis begins with fundamental mechanistic insights into lithium–oxygen electrochemistry and associated challenges, followed by a critical evaluation of state-of-the-art MOF synthesis strategies. Particular emphasis is placed on structure–property correlations governing electrochemical performance, where optimized MOF configurations consistently deliver enhanced discharge capacity, remarkable rate capability, and extended cycle life. Despite the substantial progress achieved by MOF-based materials in LOBs, critical challenges remain to be addressed for further enhancing their electrochemical performance.

1) The rational design and scalable fabrication of MOF-based materials with exceptional stability, reliable regenerability, and cost-effectiveness remain critical challenges in sustainable energy applications. Conventional MOFs exhibit inherent limitations in environmental tolerance due to their labile coordination bonds, demonstrating pronounced sensitivity to pH fluctuations, solvent variations, and electric field exposure. Furthermore, MOF derivatives frequently undergo undesirable phase transitions or sulfidation during electrochemical cycling processes. These fundamental constraints necessitate comprehensive optimization of the chemical, thermal, and electrochemical stability of MOF-based materials as prerequisite criteria for practical implementation. Strategic enhancement of coordination strength in MOF architectures must be prioritized while systematically monitoring the structural integrity and compositional evolution of MOFs throughout operation cycles. From an industrial perspective, material recyclability and production economics emerge as equally vital parameters requiring urgent attention. Current synthesis methodologies for MOF-based functional materials predominantly remain confined to laboratory-scale demonstrations. This technological gap underscores the imperative need for developing innovative yet simplified preparation protocols that enable mass production of high-performance MOF functional materials with superior cycling durability and competitive manufacturing costs.
2) AI and ML are fundamentally transforming the research paradigm for MOFs. Utilizing generative models, including generative adversarial networks and variational autoencoders, for the inverse design of novel structures, and employing algorithms such as graph neural networks to conduct high-throughput screening of vast virtual MOF libraries, AI and ML enable the rapid identification of materials exhibiting optimal target properties, such as superior gas adsorption capacity or separation selectivity. This capability transcends the limitations of conventional trial-and-error methodologies. Furthermore, ML models accurately predict structure–property relationships, synthesis conditions, and material stability, thereby guiding rational design and experimental optimization to significantly accelerate the research and development cycle. Despite persistent challenges related to data quality, model interpretability, and the validation of synthetic feasibility, AI-driven closed-loop research frameworks (integrating design, synthesis, and characterization) alongside multiobjective optimization techniques are poised to continuously drive breakthroughs in MOF applications for energy storage, environmental remediation, and beyond, ushering in a new era of intelligent materials development.
3) A comprehensive mechanistic understanding of the dynamic behaviors for MOF-based catalysts at electrochemical interfaces represents the cornerstone for surpassing current performance limitations in LOBs. While advanced *in situ* characterization techniques, including X-ray absorption spectroscopy and operando Raman analysis, have partially unveiled the coordination evolution of MOF catalytic sites during oxygen redox processes, critical knowledge gaps persist regarding molecular-level interfacial phenomena. These unresolved challenges encompass charge transfer mechanisms at Li_2_O_2_/MOF interfaces and degradation pathways of organic linkers. Future research priorities should focus on developing multimodal *in situ* analysis platforms that synergistically combine synchrotron-based X-ray absorption near-edge structure computed tomography with cryogenic electron microscopy, enabling multiscale dynamic monitoring ranging from ångström-level electronic configurations to micrometer-scale electrode morphological transformations. Theoretical frameworks must evolve beyond static computational models to incorporate nonequilibrium kinetic simulations, where ab initio molecular dynamics could elucidate transport barriers and solvation sheath evolution of ionic species within MOF nanochannels. Concurrently, the field requires standardized testing protocols to decouple intrinsic catalytic contributions from composite electrode effects, coupled with AI-powered failure mode diagnostic tools for quantitative analysis of capacity fading mechanisms, including parasitic reaction accumulation, pore blockage, and metal dissolution. This integrated approach will establish closed-loop feedback for precision engineering of next-generation MOF-based oxygen electrodes.
4) MOFs have emerged as promising candidates for LOBs catalysts due to their inherent porous architectures and tunable frameworks. However, their practical implementation is significantly hindered by intrinsically low electrical conductivity that impedes efficient electron transfer kinetics. While recent advances in MOF composites and their derivatives demonstrate enhanced electronic conductivity, reduced tap density, and structural diversity, the intrinsic propensity toward self-agglomeration in these derivatives requires critical attention. To address these challenges, the strategic development of conductive MOF architectures that harmonize electron transport pathways with porous frameworks represents a promising avenue for constructing high-efficiency MOF-based catalytic systems.
5) In LOBs systems, current MOF-based catalysts remain constrained by suboptimal rate capabilities, elevated overpotentials, and limited cycling stability, primarily attributable to insufficient OER activity. To propel advancements in this field, a structure-guided engineering strategy holds significant promise for the targeted synthesis of MOF-based catalysts with tailored dual ORR and OER functionality. Notably, OER performance optimization could be achieved through the strategic incorporation of 3d transition metal species (e.g., Fe, Co, Ni, Mn) or noble metal centers (e.g., Ir, Ru) as catalytically active sites. Furthermore, precisely engineered defect configurations offer an alternative pathway to amplify active site density and enhance intrinsic catalytic activity, thereby addressing the fundamental limitations of conventional MOF architectures. Beyond the optimization of the cathode catalyst, extending the functional design of MOF materials to other critical cell components also presents tremendous potential. For separator modification, constructing MOF-based ion-selective coatings can exploit their well-defined pore structures as ion sieves. This effectively blocks the shuttling of polysulfides (or superoxide radicals) while promoting a uniform Li^+^ flux, thereby suppressing dendrite growth and mitigating anode corrosion. In the realm of solid-state electrolytes, incorporating MOFs as fillers within polymer matrices (e.g., PEO) or as hosts for single-ion conductors leverages their high surface area and Lewis acidic sites to significantly dissociate lithium salts, create rapid Li^+^ transport pathways at interfaces, and immobilize anions through interactions with the MOF framework, thus enhancing the Li^+^ transference number. Although these separator- and electrolyte-level designs do not directly participate in the ORR and OER, they fundamentally optimize the reaction microenvironment and stabilize the lithium anode interface. Collectively, they synergistically enhance the overall energy efficiency and cycle life of LOBs. Meanwhile, research on the application of MOF-based catalysts under realistic air atmosphere remains relatively limited. The core challenges include: impurities such as water vapor and CO_2_ in the air readily trigger side reactions, generating insulating byproducts like Li_2_CO_3_ and LiOH, which lead to electrode passivation; MOF materials are prone to structural collapse and deactivation of metal active sites in humid and CO_2_-containing environments; the low O_2_ concentration and complex mass transport impose higher demands on the pore structure and conductivity of the electrodes; moreover, the complicated reaction pathways and difficult control of intermediates pose severe challenges to the selectivity and bifunctional activity of catalysts, which is the main reason why most studies still use pure oxygen. Therefore, multidimensional modification of MOF-based materials is necessary to enhance the overall battery performance.
6) Future advancements of MOFs in LOBs will center on multiscale structural engineering and integrated functional innovation. Extensive studies have demonstrated that the topological tunability of MOFs enables atomic-level precision in orchestrating pore size distribution, surface chemistry, and metal node coordination environments, thereby offering a versatile platform for regulating Li_2_O_2_ nucleation and decomposition pathways. To transcend conventional synthesis paradigms, next-generation research should prioritize advanced strategies, including dynamic templating approaches and topology-guided assembly techniques to construct 3D conductive networks with gradient pore architectures. Illustratively, conformal deposition of ultrathin conductive coatings (e.g., graphene or MXene) via atomic layer deposition on MOF surfaces presents a viable solution for preserving their inherent high surface area while circumventing intrinsic conductivity limitations. Biomimetic mineralization strategies further allow molecular-level integration of MOFs with biomass-derived carbon matrices, generating hierarchical porous heterostructures that exhibit synergistic interplay between enhanced ion transport kinetics and mechanical robustness. Crucially, interface engineering within MOF-based composites emerges as a pivotal frontier—the construction of strongly coupled interfaces through covalent anchoring, π-π stacking, or electrostatic self-assembly between MOFs and 2D materials (such as black phosphorus or transition metal dichalcogenides) could effectively modulate charge redistribution dynamics and stabilize reactive intermediates like LiO_2_ species.
7) The key challenge for industrializing MOF-based catalysts is achieving long-term stability under realistic operating conditions while enabling low-cost, scalable production. Laboratory-tested catalysts often fail under ambient air, high current, or deep cycling due to structural collapse and side reactions. Therefore, prioritizing robust structural design and cost-effective manufacturing is essential for practical deployment. When scaling up from coin cells to practical pouch or cylindrical batteries, MOF-based LOBs face engineering challenges including poor mass transport and conductivity within thick electrodes, structural collapse, aggravated side reactions in ambient air, and poor batch-to-batch consistency in large-scale fabrication. Corresponding strategies include: constructing hierarchically porous electrodes with 3D conductive networks to alleviate polarization inhomogeneity and uneven product distribution in thick electrodes; employing binder-free self-supporting structures or carbon coating to enhance mechanical stability; hydrophobically modifying MOFs, together with low-hygroscopicity electrolytes and efficient gas diffusion layers, to suppress side reactions induced by moisture and CO_2_; and developing scalable processes such as continuous-flow synthesis, mild calendering, and online quality control to ensure batch consistency. Through such multilevel synergistic optimization spanning material, electrode, and battery scales, the practical application bottleneck of MOF-based lithium–oxygen batteries is expected to be overcome.

In summary, the continuous development and optimization of MOFs and their derivatives with tunable architectures and diversified compositions have unveiled unprecedented potential in energy storage applications. It is anticipated that MOF-based materials will play a pivotal role in advancing next-generation LOB systems, offering innovative pathways to achieve breakthrough advancements in energy density and cycling stability.
